# Supplementary material for: Diversity-Related, Student-Led National Medical Organizations: Leadership Opportunities for Learners
Source: MedEdPORTAL. 2024 Dec 27;20:11477. doi: 10.15766/mep_2374-8265.11477 (PMC11671812; doi:10.15766/mep_2374-8265.11477)
Supplement: Supplementary file 1 — Facilitator Guide.docxPre- and Postworkshop Survey.docxNMOs Presentation.pptxExample SNMA Strategic Plan.docxNMOs Activities Handout.docxDr. Freeman SNMA Testimonial.mp4Fae MSPA Testimonial.mov [file mep_2374-8265.11477-s001.zip › _Educational Summary Report_11477.pdf]

MedEdPORTAL® The AAMC Journal of Teaching and Learning Resources

Original Publication Open Access

Diversity-Related, Student-Led National Medical Organizations: Leadership Opportunities for Learners

Nicholas N. Brutus, MD*, Dennis J. Spencer, MD, PhD, Derek Huell, Yaritzy M. Astudillo, MD, Austen Ott, Joyce H. Lee, MS, Alec J. Calac, PhD, John P. Sánchez, MD, MPH

*Corresponding author: Nicholas.n.brutus@gmail.com

Abstract

Introduction: In light of the lack of diversity in academic medicine leadership, diversity-related, student-led national medical organizations (NMOs) provide a space for solace and reprieve among common peers while providing an opportunity to develop leadership competencies in a supportive environment. Despite the impact NMOs have had on cultivating generations of leaders in medicine, trainees may not identify opportunities for leadership development that are transferable to future careers in academic medicine. **Methods:** We designed and implemented a dynamic 60-minute workshop with an interactive PowerPoint presentation, author-owned video testimonials (from past student leaders of NMOs), two case presentations, and reflection exercises. We assessed learner self-perceived confidence via workshop surveys. The target audience of this module was medical trainees, including medical students, residents, and fellows. **Results:** Forty-three workshop attendees across three sites submitted partial or complete survey evaluations. Respondents included medical students (77%), with the remainder self-identified as either postbaccalaureate students, residents/fellows, academic faculty, or physicians. A comparison of pre- and postresponses showed a statistically significant increase in confidence in addressing each of the four educational objectives. Participants felt the case presentations offered relevant applicable examples. **Discussion:** For many trainees, the role that diversity-related NMOs play in developing leadership competencies may be unclear and not articulated in traditional medical curricula. In this module, we provide examples of how NMOs facilitate leadership development and may encourage our diverse trainees to eventually become academic faculty.

Keywords

National Medical Organizations, Leadership Development, Case-Based Learning, Mentoring/Coaching, Student Affairs, Diversity, Equity, Inclusion

Educational Objectives

By the end of this activity, learners will be able to:

1. Describe the role of diversity-related, student-led national medical organizations (NMOs) in developing future diverse leaders.
2. Describe engagement and leadership opportunities for trainees through diversity-related, student-led NMOs.
3. Review examples of successful trainee-driven activities and leadership competencies gained through diversity-related, student-led NMOs.
4. Highlight the experiences of and competencies gained by members of diversity-related, student-led NMOs.

Citation:
Brutus NN, Spencer DJ, Huell D, et al. Diversity-related, student-led national medical organizations: leadership opportunities for learners. *MedEdPORTAL*. 2024;20:11477.
https://doi.org/10.15766/mep_2374-8265.11477

Introduction

In recent decades, diversity and equity initiatives in academic medicine have been increasingly studied and are being recognized as institutional priorities.[1-3] Multiple studies have demonstrated that greater physician diversity and racial/ethnic concordance between physicians and patients result in greater likelihood of physicians providing care for underserved populations and overall improved patient outcomes.[4-6] Despite desirable outcomes that have motivated decades of workforce development initiatives, the medical student and physician population from racial and ethnic minority backgrounds fail to reflect the diversity of the general population, showing minimal improvement in representation over the last 40 years.[7,8]

Copyright © 2024 Brutus et al. This is an open-access publication distributed under the terms of the Creative Commons Attribution license.

This discrepancy in representation extends across academic medicine, including medical school teaching faculty and administrative leadership. Data from the AAMC illustrate only 6% of medical school faculty identify as Latina/o/x/e, Hispanic, or of Spanish origin+ (LHS+); 4% as Black or African American; >1% as American Indian or Alaska Native; and even less as Native Hawaiian or other Pacific Islander.[9,10] This contrasts significantly from U.S. Census data: 19% LHS+, 14% Black, and 1% American Indian/Alaska Native.[11] Furthermore, overall underrepresented in medicine representation among department chairs and deans has decreased over time relative to their groups' representation in the U.S. Census.[12]

Limited exposure to diverse leaders in academic medicine may undermine interest among diverse medical trainees to consider this career path.[13,14] Structural and historical factors have contributed to homogeneity in academic medicine. As described in this module, diversity-related, student-led national medical organizations (NMOs) were created, and have been sustained, to raise awareness and to create unique opportunities for diverse learners to thrive within medicine and academic medicine.[15-17] Accordingly, a survey of 94 North American academic health centers reported that only 65% offered a formal internal faculty leadership program,[18] thereby narrowing the path towards gaining the skills and mentorship required to develop into effective leaders. Therefore, an informed strategy for academic medicine, in the evolving landscape, is to develop vertical leadership pathways and leadership competencies for diverse trainees interested in academic medicine.

There are several NMOs committed to ensuring the successful training and advancement of diverse medical students. This includes the Student National Medical Association (SNMA)—first chapter 1964,[19] Latino Medical Student Association (LMSA)—first chapter 1972,[20] Association of Native American Medical Students (ANAMS)—first chapter 1975,[21] Asian Pacific American Medical Student Association (APAMSA)—first chapter 1995,[22] and the Medical Student Pride Alliance (MSPA)—first chapter 2018,[23] among others. Many of these NMOs had established chapters at medical schools prior to the creation of medical school offices for diversity, equity, and inclusion (DEI) and have continued to play an important role in supporting learners despite competing contemporary provocations, such as those calling for restructuring offices for DEI in states including Texas, Florida, and Iowa.[24] Each organization listed was formed to serve a specific population of medical students, but all share a common mission of supporting future physicians and improving care for patients belonging to their communities. These organizations, many led by medical students, allow for learners to develop as leaders and professionals at local, regional, and national forums.

NMOs can provide members with their first exposure to duties and responsibilities directly translatable to academic sectors. The experience gained through work in these organizations builds self-efficacy, leadership skills, and competencies that are needed for academic career advancement. These roles coupled with a supportive environment of peers with similar backgrounds can provide a nurturing platform for the development of medical trainees. Still, it remains critical to provide structured opportunities to reflect upon how their engagement with NMOs is directly linked to the core competencies of faculty development in academic medicine.[18,25]

Building the Next Generation of Academic Physicians (BNGAP) promotes the mission of academic career development for diverse medical trainees. BNGAP hosts national conferences/seminars and has many established local chapters to raise awareness of opportunities to engage with medical school offices and organizations towards the development of leadership competencies. The BNGAP Curriculum Committee, in partnering with NMOs, specifically sought to reach consensus on the steps needed to pursue careers in academic medicine. Several other educational workshops from the curriculum have been published in *MedEdPORTAL* and focus on trainee engagement with the offices for DEI,[26] student affairs,[27] admissions,[28] and education.[29] These workshops provide a foundation for trainees to become involved within each office at their respective institutions, providing a groundwork for local involvement. To convey the value of engagement and leadership opportunities provided by NMOs, this workshop was created to illuminate the distinct value of national opportunities in sculpting the early training of diverse medical students and trainees to become leaders in academic medicine.

Methods

Development

This workshop focused on NMOs' relevance to leadership competency development and was developed using Kern's six-step framework for curriculum development.[30] The Kern approach has proven to be a successful systematic method for other BNGAP modules using its stepwise approach for designing medical education curricula.[26-29] We first identified the problem and conducted a general needs assessment (step 1) by surveying trainees to ascertain their interests in academic medicine careers. We also sought direct feedback from BNGAP liaisons and student leaders from NMOs including SNMA, LMSA,

Copyright © 2024 Brutus et al. This is an open-access publication distributed under the terms of the Creative Commons Attribution license.

APAMSA, MSPA, and ANAMS on leadership development opportunities through direct feedback. As such, we actively engaged with both students and faculty from the outset of developing the design and implementation of this module. An NMO leader or appropriate representative consented to the use of their organization's logo and other materials in the module, as applicable. In the next step of Kern's framework (step 2), we sought to incorporate faculty leadership competencies, as previously described by Lucas and colleagues,[18] to identify core skills trainees develop through leadership roles within NMOs. The third step was identifying our goals and objectives, which were decided on by consensus among our co-authors. Bloom's taxonomy[31] was applied to assure measurable outcomes. This module utilized an interactive PowerPoint presentation, author-owned video testimonials highlighting former and current NMO student leaders, and case presentations. These educational strategies (step 4) have been used in prior published BNGAP modules.[26-29] Each component is meant to actively engage the audience through interactive conventions, such as reflection, problem-solving, and sharing of perspectives among participants. In execution of Kern's fifth step, implementation of this workshop was during BNGAP national conferences designed for trainees across the academic spectrum (medical students, residents, and fellows) who were interested in engaging with content pertinent to pursuing careers in academic medicine. To evaluate the effectiveness of our curricular innovation (Kern's sixth step), we distributed and then collected pre- and postworkshop surveys that provided valuable feedback on the workshop's content and design. The Rutgers Health Sciences Institutional Review Board Newark approved this project.

Faculty identified to deliver the content were chosen based on their prior experiences as student leaders in NMOs and/or current role as faculty advisors of NMOs. While a priori knowledge of NMOs is not necessarily a prerequisite to deliver this workshop, faculty presenters with this knowledge can serve as both presenter and a role model able to speak from their lived experience. The target audience (medical students, residents, and fellows) may or may not have any familiarity with NMOs, which acknowledges that much of the content may be completely new for some learners.

Implementation

Presenters are expected to review facilitator guide before implementing workshop (Appendix A). The 60-minute workshop begins with a pre-workshop survey (Appendix B), where participants are asked about their demographic information, knowledge in identifying leadership opportunities available through diversity-related, student-led NMOs, and their confidence in working with NMOs to drive institutional change. After completion of the survey, presenters deliver a PowerPoint presentation (Appendix C). The presentation begins with an introduction to NMOs, their structure, governance, and function. An example Strategic Planning document is included (Appendix D) as an example from SNMA (provided with permission). The next section proceeded to describe engagement and leadership opportunities for students in NMOs. Appendix E is a handout that is distributed during this section that explicitly illustrates how common activities performed by NMOs align with core leadership competencies. Participant engagement is further maximized with a series of reflection exercises, complemented with author-owned video testimonials (Appendices F & G). These exercises and videos produced by prior NMO leaders describe how their involvement in their respective organizations supported their upward leadership trajectory. Based on learner feedback, case-based exercises included in the workshop allow participants to work through true scenarios, using knowledge obtained through the module, encountered by student NMO members.

We used IBM SPSS Statistics version 28.0 to run statistical analyses of the quantitative data in this cause-and-effect study.

Results

This workshop was implemented during BNGAP conferences that took place at four academic campuses: University of Oklahoma College of Medicine (Oklahoma City, Oklahoma), John P. and Kathrine G. McGovern Medical School at UTHealth (Houston, Texas), Joan and Sanford I. Weill Medical College of Cornell University (New York, New York), and Roy J. and Lucille A. Carver College of Medicine (Iowa City, Iowa). A total of 43 trainees completed partial or full workshop evaluations. The workshop was facilitated by a total of five presenters (one pair and three single presenters), including an assistant dean for diversity and inclusion, an associate professor of internal medicine, a professor of emergency medicine, and two associate deans of student affairs.

Of the 43 participants, 35 (81%) identified as medical students, two (5%) identified as postbaccalaureate students, and two (5%) others identified as a fellow and resident. Participants hailed from six different states and the Dominican Republic.

Among the 43 respondents, 13 (30%) identified as Black or African American, 11 (26%) as Hispanic or Latino, 10 (23%) as White, seven (16%) as Asian, and three (7%) identified as American Indian or Alaska Native. Nineteen (44%) identified

Copyright © 2024 Brutus et al. This is an open-access publication distributed under the terms of the Creative Commons Attribution license.

as male and 20 (46%) as female. Three (7%) identified as gay, lesbian, or bisexual.

In assessing attendee background experience, prior to administering the workshop, participants were asked "How knowledgeable are you in identifying leadership opportunities for trainees to become engaged through the National Medical Organization?" In response, 20 (46%) attendees replied *not knowledgeable*, six (14%) replied *somewhat knowledgeable*, five (12%) replied *knowledgeable*, and one (2%) replied *very knowledgeable*.

Additionally, the efficacy of the workshop was determined by asking attendees "To what extent do you agree that the workshop learning objectives were met?" All attendees *agreed* or *strongly agreed* that the learning objectives were met.

On the pre- and postworkshop questionnaire assessment, attendees were asked to select their level of confidence (0 = *no confidence*, 4 = *complete confidence*) regarding two national medical organization-related objectives. Paired-sample Wilcoxon signed rank test was used to assess for a statistically significant difference in median. For the statement "Find a national medical organization aligned with your identity," the preworkshop mean and median values were 2.6 and 3.0, respectively, and postworkshop mean and median values were 3.8 and 4.0, respectively ($p = .01$). For the statement "Work with national medical organizations to drive institutional change," the preworkshop mean and median values were 1.7 and 2.0, respectively, and postworkshop mean and median values were 3.6 and 4.0, respectively ($p = .01$).

Participant comments and suggestions for the workshop were collected to identify positive aspects of the curriculum and how to further improve the module. Overall, the participants found the workshop to be informative, interactive, and felt the case presentations offered applicable examples. Several respondents commented on their ability to understand and apply the presented information: "It demonstrated clear pathways for making an impact through National Medical organizations."

In terms of how to improve the workshop, respondents recommended offering institution specific examples of leadership competency development through NMOs and offered examples: "outreach, MCAT prep, Hispanic heritage month/African American Month/pride events."

Discussion

Diversity-related, student-led NMOs have provided students with a community of peers, faculty, and, importantly, mentors to serve as role models to demystify the pathway to academia. For trainees, involvement in these organizations has also served as a critical leadership development experience, even if they may not consciously recognize it as such. This dissonance is supported by many of our respondents who indicated a lack of knowledge around identifying leadership opportunities for trainees in NMOs. As indicated by the feedback from participants, recognizing leadership competencies through common NMO experiences that are translatable to academic/administrative skills helps to build self-efficacy to pursue future faculty roles.

Reflecting on in-person participant feedback and qualitative responses to the model, the educational objectives were well met. The presentation was proved to benefit medical trainees, and, importantly, it provided a sense of validation to those already involved in NMO work and its future career implications. This was confirmed as there was also a statistically significant increase in self-perceived confidence across objectives. Based on feedback, the module was further developed to meet trainee needs over its multiple implementations. We designed this workshop to be delivered to an ideal audience size of 20-30 people per session. This midsize group has proven to be an ideal audience size, through other BNGAP curricula, to promote trainee engagement. The case presentations were built using the social cognitive theory framework that served as the scaffold for this workshop. It is important to note that the current remote climate for medical trainee teaching does pose challenges for discussion on sensitive topics. Utilization of breakout rooms and/or separate focus groups for further case discussion is advised for facilitators when conducting the workshop remotely. Despite this, most participants valued the relevance of the content presented, and it sparked conversation considering the intersectionality of skills acquired through engagement with NMOs.

Medical trainee engagement in diversity-related NMOs provides an opportunity to develop communication skills on relevant topics in a supportive environment, share best practices to facilitate diversity-related advocacy, and acquire any number of other leadership competencies. In this module, we provided examples of how these organizations are facilitators of leadership development, which can include the acts of working on a board, organizing pathway activities, and participating in mission-centered advocacy. A review of leadership competencies as described by Lucas and colleagues[14] was shared with participants to explicitly reinforce the overlap of these skills with the experiences and examples presented throughout the module. A potential area to improve delivery of this module would be to

Copyright © 2024 Brutus et al. This is an open-access publication distributed under the terms of the Creative Commons Attribution license.

have trainees review and relate leadership competencies to their own experiences prior to the presentation.

This presentation benefitted from facilitators representing different NMOs, having their own unique experiences with the organizations. Facilitators with first-hand experiences to draw from within these organizations helped to contextualize the material being presented, which was evident from participant feedback on how they truly "appreciated learning about the importance and function of national medical organizations." An example of this in action could be seen in educational objective 4, "Highlight the experiences of and competencies gained by members of diversity-related, student-led NMOs," where a cofacilitator was able to reflect on their specific role in the development and implementation of a specific initiative within SNMA. Based on feedback from participants, we recommend future facilitators modify case scenarios (Appendix C, slides 32-38) and/or discuss their own experiences in NMOs (Appendix C, slide 40) to further personalize the presentation to maximize engagement with the audience. This also provides another opportunity to explicitly name the relevant leadership competencies they were personally able to develop.

One noted limitation to the generalizability of this innovation is our small number of participants. As a sample of convenience, these participants were primarily medical students, residents, and fellows who were already attending a professional development seminar. Given this unique sampling, however, it may be expected that this might represent a more informed subset of medical trainees regarding their baseline understanding of leadership opportunities in NMOs. It is also important to consider that this module was a single 60-minute implementation to a group of medical trainees with no further follow-up on assessment of retained knowledge and/or confidence. Possible further evaluation of learners postmodule, in accordance with Bloom's taxonomy,[31] may involve assessing trainee future retention of knowledge and self-reported application of information learned. Future long-term assessment of whether trainees go on to seek academic positions after being introduced to the module would be the most direct assessment of module influence. Future modules may also benefit from data disaggregation of medical education level (first-year medical student, second-year medical student, first-year resident, second-year resident, etc.) for pre- and postworkshop analysis with larger sample populations. This may provide a greater opportunity for workshop development, relative to the variance in baseline knowledge, to create a lasting impact and improve workshop effectiveness.

A possible innovation for overcoming long-term attrition of confidence would be through dissemination of handouts (via paper or QR code) at the end of the presentation showing local and/or national opportunities with correlation to leadership competencies. By providing examples of NMO leadership development programs, or relevant activities, trainees can see practical engagement opportunities to further complement the material presented even if not present at their own institution. However, long-term impact of this module may be limited to access and exposure to opportunities at trainees' respective institutions.

Over the past 60 years, diversity-related, student-led NMOs have created a legacy of activism, community service, and advocacy. As a natural extension of trainee involvement in such organizations, they develop skills and experiences that prepare them to be leaders in their communities as well as in academic medicine. There is an opportunity to further support diverse trainee participation in these organizations as an intentional means towards developing leadership competencies to meet the need for the next generation of academic leaders equipped to address those pressing equity issues impacting medicine and society. This workshop serves to bring awareness to the important role for diversity-related NMOs but is only a start. Future qualitative studies aimed to better understand identity formation as a leader while serving their NMO could provide greater clarity on how to better support the development of diverse leaders through codified common themes of perceived facilitators and barriers. Additional curricula could then be developed to address additional needs.

Appendices

A. Facilitator Guide.docx
B. Pre- and Postworkshop Survey.docx
C. NMOs Presentation.pptx
D. Example SNMA Strategic Plan.docx
E. NMOs Activities Handout.docx
F. Dr. Freeman SNMA Testimonial.mp4
G. Fae MSPA Testimonial.mov

All appendices are peer reviewed as integral parts of the Original Publication.

Nicholas N. Brutus, MD: First-Year Resident, Department of Urology, Yale New Haven Hospital; ORCID: https://orcid.org/0000-0003-4785-5308

Copyright © 2024 Brutus et al. This is an open-access publication distributed under the terms of the Creative Commons Attribution license.

Dennis J. Spencer, MD, PhD: Instructor of Pediatrics and Faculty Advisor, Office of Recruitment and Multicultural Affairs, Harvard Medical School; Associate Program Director, Boston Combined Residency Program, Boston Children's Hospital

Derek Huell: Third-Year Medical Student, New York University Grossman School of Medicine

Yaritzy M. Astudillo, MD: Second-Year Clinical Fellow, Division of Nephrology, Department of Pediatrics, Ann & Robert H. Lurie Children's Hospital of Chicago

Austen Ott: Third-Year Medical Student, University of Minnesota Medical School

Joyce H. Lee, MS: Fourth-Year Medical Student, Medical College of Wisconsin; National President, Asian Pacific American Medical Student Association

Alec J. Calac, PhD: Third-Year Medical Student, UC San Diego School of Medicine; Past President, Association of Native American Medical Students

John P. Sánchez, MD, MPH: Dean, Universidad Central Del Caribe, School of Medicine; Executive Director, Latino Medical Student Association

Disclosures
None to report.

Funding/Support
None to report.

Ethical Approval
The Rutgers Health Sciences Institutional Review Board Newark approved this project.

References

1. Steinbrook R. Diversity in medicine. *N Engl J Med*. 1996;334(20):1327-1328. https://doi.org/10.1056/NEJM199605163342011
2. Cooper LA, Powe NR. *Disparities in Patient Experiences, Health Care Processes, and Outcomes: The Role of Patient–Provider Racial, Ethnic, and Language Concordance*. The Commonwealth Fund; 2004. Accessed November 4, 2024. https://www.commonwealthfund.org/sites/default/files/documents/___media_files_publications_fund_report_2004_jul_disparities_in_patient_experiences__health_care_processes__and_outcomes__the_role_of_patient_provide_cooper_disparities_in_patient_experiences_753_pdf.pdf
3. Saha S, Guiton G, Wimmers PF, Wilkerson L. Student body racial and ethnic composition and diversity-related outcomes in US medical schools. *JAMA*. 2008;300(10):1135-1145. https://doi.org/10.1001/jama.300.10.1135
4. Shen MJ, Peterson EB, Costas-Muñiz R, et al. The effects of race and racial concordance on patient-physician communication: a systematic review of the literature. *J Racial Ethn Health Disparities*. 2018;5(1):117-140. https://doi.org/10.1007/s40615-017-0350-4
5. Marrast LM, Zallman L, Woolhandler S, Bor DH, McCormick D. Minority physicians' role in the care of underserved patients: diversifying the physician workforce may be key in addressing health disparities. *JAMA Intern Med*. 2014;174(2):289-291. https://doi.org/10.1001/jamainternmed.2013.12756
6. Cooper LA, Roter DL, Johnson RL, Ford DE, Steinwachs DM, Powe NR. Patient-centered communication, ratings of care, and concordance of patient and physician race. *Ann Intern Med*. 2003;139(11):907-915. https://doi.org/10.7326/0003-4819-139-11-200312020-00009
7. Morris DB, Gruppuso PA, McGee HA, Murillo AL, Grover A, Adashi EY. Diversity of the national medical student body—four decades of inequities. *N Engl J Med*. 2021;384(17):1661-1668. https://doi.org/10.1056/NEJMsr2028487
8. Talamantes E, Henderson MC, Fancher TL, Mullan F. Closing the gap—making medical school admissions more equitable. *N Engl J Med*. 2019;380(9):803-805. https://doi.org/10.1056/NEJMp1808582
9. Guevara JP, Wade R, Aysola J. Racial and ethnic diversity at medical schools—why aren't we there yet? *N Engl J Med*. 2021;385(19):1732-1734. https://doi.org/10.1056/NEJMp2105578
10. Diversity in medicine: facts and figures 2019. Association of American Medical Colleges. Accessed November 26, 2024. https://www.aamc.org/data-reports/workforce/report/diversity-medicine-facts-and-figures-2019
11. QuickFacts. US Census Bureau. Accessed November 4, 2024. https://www.census.gov/quickfacts/fact/table/US/POP010220#POP010220 on 08/2024
12. Kamran SC, Winkfield KM, Reede JY, Vapiwala N. Intersectional analysis of US medical faculty diversity over four decades. *N Engl J Med*. 2022;386(14):1363-1371. https://doi.org/10.1056/NEJMsr2114909
13. Yanchus NJ, Carameli KA, Ramsel D, Osatuke K. How to make a job more than just a paycheck: understanding physician disengagement. *Health Care Manage Rev*. 2020;45(3):245-254. https://doi.org/10.1097/HMR.0000000000000218
14. Cosgrove S, Moore-Monroy M, Jenkins C, et al. Community health workers as an integral strategy in the REACH U.S. program to eliminate health inequities. *Health Promot Pract*. 2014:15(6);795-802. https://doi.org/10.1177/1524839914541442
15. Okike K, Phillips DP, Swart E, O'Connor MI. Orthopaedic faculty and resident sex diversity are associated with the orthopaedic residency application rate of female medical students. *J Bone Joint Surg Am*. 2019;101(12):e56. https://doi.org/10.2106/JBJS.18.00320
16. Bhutta N, Chang AC, Dettling LJ, Hsu JW, Hewitt J. Disparities in wealth by race and ethnicity in the 2019 Survey of Consumer Finances. Board of Governors of the Federal Reserve System.

Copyright © 2024 Brutus et al. This is an open-access publication distributed under the terms of the Creative Commons Attribution license.

Updated September 28, 2020. Accessed November 26, 2024. https://doi.org/10.17016/2380-7172.2797

17. Youngclaus J, Roskovensky L. An updated look at the economic diversity of U.S. medical students. *AAMC Analysis in Brief*. 2018; 18(5):1-3. https://www.aamc.org/media/9596/download?attachment

18. Lucas R, Goldman EF, Scott AR, Dandar V. Leadership development programs at academic health centers: results of a national survey. *Acad Med*. 2018;93(2):229-236. https://doi.org/10.1097/ACM.0000000000001813

19. Odom Black KL. Celebrating 40 years of service, advocacy, and leadership within the Student National Medical Association 2003-2004 programmatic agenda. *J Natl Med Assoc*. 2003;95(11):1024, 1028-1032.

20. Sánchez JP, Rodriguez D, eds. *Latino, Hispanic, or of Spanish Origin+ Identified Student Leaders in Medicine: Recognizing More Than 50 years of Presence, Activism, and Leadership*. Springer Nature; 2023. https://doi.org/10.1007/978-3-031-35020-7

21. Mission. Association of Native American Medical Students. Accessed November 27, 2024. https://www.anamstudents.org/about

22. Li BUK. Asian Pacific American medical students: beyond the “golden stethoscope.” *Asian Am Pac Isl J Health*. 1996;4(4):306-312.

23. Goetz TG, Zucker S, Keyes T, Gisondi M. Medical Student Pride Alliance: the first national LGBTQ+ medical student affinity organisation. *Med Educ*. 2020;54(5):471-472. https://doi.org/10.1111/medu.14112

24. Sailer JD. Comprehensive restructuring: diversity, equity, and inclusion at the University of Texas Austin. National Association of Scholars. January 19, 2023. Accessed November 4, 2024. https://www.nas.org/reports/comprehensive-restructuring/full-report

25. Lucas R, Kothari P, Adams C, Jones L, Williams VN, Sánchez JP. We are all leaders: introducing self-leadership concepts through the lens of improving diversity in the health care workforce. *MedEdPORTAL*. 2020;16:11011. https://doi.org/10.15766/mep_2374-8265.11011

26. Del Castillo N, Pischerio JP, Brewster C, Martinez D, Sánchez JP. Office of Diversity, Equity, and Inclusion: engagement and leadership opportunities for trainees. *MedEdPORTAL*. 2022;18: 11282. https://doi.org/10.15766/mep_2374-8265.11282

27. Nakae S, Haywood Y, Love LJ, Kothari P, Saldaña F, Sánchez JP. Office of student affairs: engagement and leadership opportunities for medical students, residents, and fellows. *MedEdPORTAL*. 2021;17:11093. https://doi.org/10.15766/mep_2374-8265.11093

28. Nakae S, Kothari P, Johnson K, Figueroa E, Sánchez JP. Office of Admissions: engagement and leadership opportunities for trainees. *MedEdPORTAL*. 2020;16:11018. https://doi.org/10.15766/mep_2374-8265.11018

29. Silva Díaz DH, Kothari P, Williams RL, et al. Office of medical education: opportunities for trainees to engage and lead in curricular innovation and reform. *MedEdPORTAL*. 2021;17: 11112. https://doi.org/10.15766/mep_2374-8265.11112

30. Thomas PA, Kern DE, Hughes MT, Chen BY, eds. *Curriculum Development for Medical Education: A Six-Step Approach*. Johns Hopkins University Press; 2016.

31. Adams NE. Bloom’s taxonomy of cognitive learning objectives. *J Med Libr Assoc*. 2015;103(3):152-153. https://doi.org/10.3163/1536-5050.103.3.010

Received: April 24, 2024
Accepted: September 16, 2024
Published: December 27, 2024

Copyright © 2024 Brutus et al. This is an open-access publication distributed under the terms of the Creative Commons Attribution license.
