# Supplementary material for: Diversity-Related, Student-Led National Medical Organizations: Leadership Opportunities for Learners
Source: MedEdPORTAL. 2024 Dec 27;20:11477. doi: 10.15766/mep_2374-8265.11477 (PMC11671812; doi:10.15766/mep_2374-8265.11477)
Supplement: Supplementary file 1 — Facilitator Guide.docxPre- and Postworkshop Survey.docxNMOs Presentation.pptxExample SNMA Strategic Plan.docxNMOs Activities Handout.docxDr. Freeman SNMA Testimonial.mp4Fae MSPA Testimonial.mov [file mep_2374-8265.11477-s001.zip › A. Facilitator Guide.docx]

*Please print or use this facilitate guide with the presentation of the PowerPoint. We recommend speakers review this document at least 24 hours before and add/modify slides to represent your institution if possible.*

Diversity-Related, Student-Led National Medical Organizations:

Leadership Development for Learners

**Facilitator Instructional Guide**

**Overall Goals**

The goal of this module is to help medical students gain awareness of the purpose, structure, and function of diversity-related, student-led national medical organizations and the opportunity to engage and develop leadership competencies through these organizations.

**Workshop Objectives**

1. Describe the role of diversity-related, student-led national medical organizations (NMO) in developing future diverse leaders;
2. Describe engagement and leadership opportunities for trainees through diversity-related, student-led NMO;
3. Review examples of successful trainee-driven activities and leadership competencies gained through diversity-related, student-led NMO;
4. Highlight the experiences of and competencies gained by members of diversity-related student-led NMO.

**Workshop Handouts and Materials**

1. Evaluation Forms
2. Case Scenarios
3. Discussion Guide
4. AV Equipment for PPT presentation
5. Pens

**Suggested Agenda and Timeline**

- Pre-Workshop Evaluation (Pre- Survey): 2 minutes
- Slide 1-30: 30 minutes
- Slides 31-38: Case discussions - 15 minutes
- Slides 39: Speaker Personal Experience - 3 minutes
- Slide 41: Summary - 3 minutes
- Slide 42: Questions and answers: 5 minutes
- Post-Workshop (Post- Survey) Evaluation: 2 minutes

**Slide Instructions**

**Slide 1:**

Add names with all professional degrees and associated institutions of facilitators to the slide.

All facilitators/moderators are encouraged to introduce themselves to the audience and discuss their respective roles at their institutions.

Facilitators may decide to disclose that they are following the guidelines and content of this peer-reviewed module.

**Slide 2**:

Facilitators should review learning objectives with participants.

**Slide 3:**

Session Overview: Facilitators should briefly discuss the session content with the participants.

Highlight that the presentation highlights some information on diversity-related, student-led national medical organizations but also interactive activities, such as reflections, videos, and case discussions to help learners gain awareness of the unique opportunities and benefits regarding leadership development through engagement with these organizations.

**Slide 4:**

Reflection: Facilitators should pose the following questions to 1-2 individuals. Everyone should take 1-2 minutes to address the questions.

*Let’s begin by reflecting on different leadership roles as well as personal and professional experiences with national medical organizations*.

This exercise is valuable to help participants appreciate that many of them have been and are engaged in NMOs. Some may have been engaged in college and have continued to be engaged during medical school and may have assumed escalating leadership roles. Personally, these organizations may have afforded an opportunity to network with concordant individuals or professionally allowed individuals to develop knowledge or skills to be a more competitive applicant to medical school or residency.

**Slide 5:**

As we highlight organizations that sculpt the leadership potential of medical students, it is important to note that this leadership training is provided by allowing students to directly run national medical organizations. Thus, this slide hosts several examples of diversity-related and general national medical organizations that are led by medical students.

It is important to note that the diversity related organizations included here are of a stand-alone nature, with their own governing documents, articles of establishment, and financial oversight. The American Medical Student Association and the American Physician Scientist Association operate similarly with complete student direction.

Contrastingly, the Student Osteopathic Medical Organization and the Organization of Student Representatives, are directly linked to a faculty-led national medical organization, which is denoted in parentheses. These organizations still provide critical leadership training to their medical organizations, but their governing documents, mission, and actions are limited to those that align with oversight from faculty-led organizations.

Additionally, this slide does not include any specialty-specific student-led organizations, which mainly manifest as medical student interest groups. These groups may often be organized at the national level, and all are overseen by faculty-led specialty-specific organizations. Thus, they do not exhibit the standalone nature of the student-led, diversity-related organizations listed here.

**Slide 6:**

This slide hosts a list of student-led, diversity-focused national medical organizations. While each organization serves a unique population of medical students and boasts a unique mission, they are similar in their exposure of medical students to leadership on a chapter, regional, or national level.

Particularly for students of diverse backgrounds, including many of whom belong to groups that are underrepresented in medicine, these organizations often serve as their initial exposure to being a leader within medicine. The initial attraction to joining and eventually leading these organizations may stem from multiple routes, including familiarity with these organizations prior to medical school and the comforting nature of joining individuals with a similar background or identity.

Regardless of the reasons that medical students join these organizations, they set the stage for a transformation of the student’s idea of the broad role of a physician in medicine and society. While all medical students enter with an understanding that physicians care for patients, these organizations offer insight into the role of physicians beyond patient interactions, from driving health policy change to leading hospital departments, the experiences offered to members and leaders of these organizations prepare them for more than an enjoyable medical school experience or successful residency application. Rather, these organizations train students to become physician leaders.

**Slide 7:**

Facilitators should comment on important common narratives that ring true across many of the diversity related NMO’s. Namely, many of these medical student organizations have a history centered on assuring their inclusion in medicine or academic medicine. At their core, NMO’s are guided by a defined vision and mission statement and follow a strategic plan set on agreed upon mission-aligned priorities. This all then informs their programming, which often includes some intentional activities around engaging with individuals in their pipeline as well as some service activity(s).

**Slide 8-10:**

These two slides highlight the history of LMSA, SNMA, and ANAMS. Slide 8 highlights a recently published open-access book on describes the activism of LHS+ medical students since the 1960s to present. The logos on the right showcase the evolution of LMSA’s logos which is described in the book.

Slide 9 highlights the timeline of SNMA’s development and growth.

Slide 10 highlights some of the Political Timeline for ANAMS’ establishment alongside AAIP

**Slide 11 - 13**

Now, we will transition into the structure and function of student-led national medical organizations.

There are several documents, including a constitution, policies and procedures manual, business plan, strategic plan, annual strategic agenda, and regional/national bylaws that guide the structure and function of these organizations that will be further explored.

Slide 12 shows sample mission statements from three NMO’s.

Slide 13 Similar to this example from LMSA, these organizations are organized hierarchically. Often, these organizations operate as local chapters at individual medical schools that report to a regional leadership board. These regional boards then report to a national leadership board.

**Slide 14:**

This slide exhibits many of the common governing documents that guide the structure and function of student-led national medical organizations. While there may be some variation between individual organizations, the documents listed here are found most frequently across all organizations.

The appendix includes a more detailed strategic plan for SNMA courtesy of Dennis Spencer MD, PhD, past SNMA CFO.

**Slide 15:**

This is an example of composition of members and leaders of the Student National Medical Association (SNMA), Latino Medical Student Association (LMSA), Association of Native American Medical Students (ANAMS), Medical Student Pride Alliance (MSPA), and Asian Pacific American Medical Student Association (APAMSA).

**Slide 16-17:**

These slides highlight the pipeline and pathways programs, and associated attributes offered by LMSA and SNMA.

**Slide 18:**

This slide provides some examples of how all the organizations are commonly engaged in community-based initiatives. Facilitators are encouraged to add in any local / national community activities with which they have participated (or led) with their NMO.

*Image References*

Tissue and Organ Donation Education Recruitment, <https://snma.org/page/communityservice>, accessed December 9th, 2023.

Sanchez JP and Rodriguez D. Latino, Hispanic, or of Spanish Origin+ Identified Students Leaders in Medicine. Recognizing More Than 50 Years of Presence, Activism and Leadership. Image by Springer Nature. Image is in the public domain. Creative Commons Attribution-NonCommercial-NoDerivatives 4.0 International License (http://creativecommons.org/licenses/by-nc-nd/4.0/).  Page 124.

The Linda Don Community Outreach Scholarship, <https://www.anamstudents.org/resources>, accessed December 9th, 2023

MSPA Prescribe it Forward Partnership <https://www.prescribeitforward.org/partners> accessed December 23rd, 2023

National Bone Marrow Outreach Initiative, <https://www.apamsa.org/bone-marrow-initiative/>, Accessed December 9th, 2023

**Slide 19:**

This slide provides some examples of how all the organizations are involved in position statements aligned with their mission.

Citations or weblink:

Affirmative Action (SNMA), https://cdn.ymaws.com/snma.org/resource/resmgr/hlpa/policy_statements/affirmative_action.pdf, accessed December 9th, 2023

Sanchez JP and Rodriguez D. Latino, Hispanic, or of Spanish Origin+ Identified Students Leaders in Medicine. Recognizing More Than 50 Years of Presence, Activism and Leadership. Image by Springer Nature. Image is in the public domain. Creative Commons Attribution-NonCommercial-NoDerivatives 4.0 International License (http://creativecommons.org/licenses/by-nc-nd/4.0/). Page

Sánchez JP, Sola O, Ramallo J, Sánchez NF, Dominguez K, Romero-Leggott V. Hispanic Medical Organizations Support for LGBT Health Issues. LGBT Health Volume 1, Number 3, 2014.

Violence Against APIA Communities, https://www.apamsa.org/rapid-response-statement-on-the-recent-violence-against-apia-communities/ (APAMSA), https://www.medpride.org/joint-statements (MSPA), access December 9th, 2023

**Slide 20:**

NMOs serve a vital role for Academic Health Centers. NMOs may represent a network of individuals that an Academic Health Center is interested in connecting with to ensure quality health care for their patients or build a diverse academic workforce to achieve educational or research excellence.

Through the pipeline activities of NMOs, NMOs may aid AHCs in achieving LCME or ACGME standards related to the recruitment of diverse candidates. For AHCs located in large Hispanic communities or Spanish speaking communities they may align with an organization like the Latino Medical Student Association to attract a larger pool of competitive applicants for medical school, residency or beyond.

NMOs serve a vital role for Academic Health Centers. NMOs may represent a network of individuals an Academic Health Center is interested in connecting with to ensure quality health care for their patients or build a diverse academic workforce to achieve educational or research excellence.

**Slide 21:**

Through the pipeline activities of NMOs, NMOs may aid AHCs in achieving LCME or ACGME standards related to the recruitment of diverse candidates.  For AHCs located with large Hispanic communities or Spanish speaking communities they may align with an organization like the Latino Medical Student Association to attract a larger pool of competitive applicants.

LCME Standard 3.3. 3.3 Diversity/Pipeline Programs and Partnerships  A medical school has effective policies and practices in place, and engages in ongoing, systematic, and focused recruitment and retention activities, to achieve mission-appropriate diversity outcomes among its students, faculty, senior administrative staff, and other relevant members of its academic community. These activities include the use of programs and/or partnerships aimed at achieving diversity among qualified applicants for medical school admission and the evaluation of program and partnership outcomes. (<http://lcme.org/publications/> Accessed on September 6, 2019)

ACGME Core Requirement - The program, in partnership with its Sponsoring Institution, must engage in practices that focus on mission-driven, ongoing, systematic recruitment and retention of a diverse and inclusive workforce of residents, fellows (if present), faculty members, senior administrative staff members, and other relevant members of its academic community.

https://www.acgme.org/Portals/0/PFAssets/ProgramRequirements/CPRResidencyImplementationTable.pdf  Accessed on September 6, 2019.

Soto-Greene M, Culbreath K, Guzman DE, **Sánchez JP**, Romero-Leggott V. Diversity and inclusion in the academic medicine workforce: encouraging medical students and residents to consider academic careers. MedEdPORTAL. Feb 2018;14:10689.

**Slide 22:**

Consider reading the following article which describes how SNMA assisted the University of Toledo in meeting its recruitment of African-American/Black candidates. Rumala, Bernice B., and Frederick D. Cason Jr. "Recruitment of underrepresented minority students to medical school: minority medical student organizations, an untapped resource." *Journal of the National Medical Association* 99.9 (2007): 1000.

The following article details APAMSAs efforts to promote personal and professional development opportunities for it’s students. Enas EA. Shifting Paradigms and Changing Horizons in Medicine: Challenges and Opportunities for the Asian Pacific American Medical Students Association (APAMSA). Asian Am Pac Isl J Health. 1996 Autumn;4(4):314-326.

The following article describes efforts by AMSA to advance primary care.  Hedgecock J1, Steyer TE.  The American Medical Student Association's contributions to advancing primary care. Acad Med. 2008 Nov;83(11):1057-9.

**Slide 23-24:**

These slides afford the opportunity for learners to watch and discuss the experiences of academic leaders describing the benefits of being engaged with diversity-related student -led organizations. Learners should be engaged to respond to the following questions - Based on the video clip:

How did engagement help with their academic, personal and/or professional development?

How did engagement help with their leadership development?

*(Optional) Facilitators can choose to add their own examples of leadership within NMOs. (for example, highlight a faculty member that serves in current NMO leadership)*

**Slide 25-26:**

These slides highlight formal leadership development opportunities that are offered through several diversity-related, student-led organizations. This is another example of a common characteristic across organizations and how the organizations are dedicated to advancing the leadership competencies of their members.

The following article details APAMSAs efforts to promote personal and professional development opportunities for its students. Enas EA. Shifting Paradigms and Changing Horizons in Medicine: Challenges and Opportunities for the Asian Pacific American Medical Students Association (APAMSA). Asian Am Pac Isl J Health. 1996 Autumn;4(4):314-326.

Sanchez JP and Rodriguez D. Latino, Hispanic, or of Spanish Origin+ Identified Students Leaders in Medicine. Recognizing More Than 50 Years of Presence, Activism and Leadership. Image by Springer Nature. Image is in the public domain. Creative Commons Attribution-NonCommercial-NoDerivatives 4.0 International License (http://creativecommons.org/licenses/by-nc-nd/4.0/).  Page 41. SALUD Summit. <https://national.lmsa.net/programming/salud2023/> Accessed on December 9, 2023

**Slide 27-30: Leadership Competencies**

**Slide 27:**

Transition slide. In this section learners will be introduced to 7 leadership competencies of importance for faculty to develop as leaders. This section lists the seven competencies and provides examples of how trainees can develop these competencies through engagement with diversity-related student-led organizations. The seven competencies stem from a ​​research paper by Lucas R, Goldman E, Scott A, Dandar V. Leadership Development Programs in Academic Medical Centers: Results of a National Survey. Academic Medicine 2018;93:229-236.

**Slide 28:**

This chart highlights common activities, supported by members of student-led national medical organizations, at times in conjunction with academic health centers. For example in Year 1-4, students might design, implement and/or evaluate institutional activities that align with the mission of the organization, such as heritage month activities.

**Slide 29:**

This chart highlights 7 core leadership competencies that are important to achieve to develop as an academic leader.

**Slide 30:**

This diagram highlights how trainees can develop these competencies through a variety of familiar activities.

**Slide 31: Case Discussion**

Transition Slide. This interactive activity can occur as one large group discussing two cases or splitting the group into two and each group focusing on one case.

**Slide 32-35: Case Scenario #1**

Facilitator or one of the learners should read case out loud (slide 32). Facilitator should ask for 1-2 volunteers to respond to each question (slide 33). Facilitator should highlight the following information when discussing questions with learners.

| Which organization would be a reasonable resource to address this issue? | SNMA, LMSA, and ANAMS as they represent the interest and development of underrepresented students entering medicine. |
| --- | --- |
| List some important outcomes to explore? | Increasing awareness of opportunities for being involved in research or professional development around skills to better prepare learners to be successful in becoming the next generation of physician researchers. |
| How do you gain buy-in from the organization and from whom? | Present how addressing this issue is mission critical in alignment with the organization’s priorities. For example, these org’s desire eliminate health disparities, and addressing the researcher pipeline has downstream ramification towards this desired end. |
| How do you gain buy-in from your medical school or external entities (NIH, Pharma, Biotech, HRSA, etc)? | Similarly as above, the NIH and many Philanthropic arms within “industry” has set for priorities to address health disparities and there is a direct strategy to expanding the diversity of the research program to help tackle these issues. |
| What leadership development competencies could you develop through this project and why? | Examples:  Working with/developing others: bringing together individuals with and across organizations to draft a resolution (or in this case, establishing / expand a novel Physician Researcher “initiative”).  Communication skills: explaining how this initiative aligns with organizational mission, vision, or strategic planning  Teambuilding: coalescing members to plan and evaluate this initiative.  Business skills: learns grant writing and expense management of a new project to address a chronic problem |

Big problems may benefit from the leverage that may be possible through partnering with Big organizations. First, determine if this question aligns at all with the organization’s mission. Can also decide whether a diversity-related NMO is preferred in this situation (as opposed to engaging initially with a non-diversity related NMO).

If it is aligned with the NMO’s mission, is this questions already being addressed as an existing strategic goal. If yes, then the student should take inventory of these resources and/or programs to help either optimize current approach vs helping to facilitate a change.

If there was nothing in place from the outset, then the student should go follow the rules of governance set by the NMO, identifying what committee to engage with to pitch this concern as impetus for a new program or initiative.

As part of the presidential agenda of then student leader Dr. Brandi Freeman’s agenda as National Presidential, the SNMA took on the tall task of addressing the growing need to increase the pool of URiM physician scientists by establishing the Physician Researcher Initiative. Two MD/PhD student leaders, Drs. Spencer and Franklin-Ford Ellis subsequently wrote and was awarded a multi-year NIH R13 grant as co/PI’s ultimately providing > $250K to fund workshops and travel awards for URiM participants in the organization’s Physician Researcher Initiative track.

PRI became part of the Executive Agenda in 2006 / 2007. Dr. Spencer helped implement in 2008 and eventually grew to a program that garnered NIH support through R13 grant mechanism

Image property of Dennis Spencer, image posted on December 9th, 2023.

**Slides 36-38: Case Scenario #2**

Facilitator or one of the learners should read case 2 out loud (slide 36). Facilitator should ask for 1-2 volunteers to respond to each question (slide 37). Facilitator should highlight the following information when discussing questions with learners.

| Which organization would be a reasonable resource to address this issue? | LMSA and MSPA. The mission of LMSA is to unite and empower current and future physicians through service, mentorship, and education to advocate for the improved health of the Hispanic & Latina/o/x community in the United States.  The mission of MSPA is to support the success of current and future LGBTQ+ medical students throughout their education and careers by fostering community and connecting members. |
| --- | --- |
| List some important outcomes to explore? | How does the resolution align with mission and strategic plan of the organization? Is their a sub-group or committee within the organization that might be most interested in the question? For LMSA, sub-groups include the LMSA-LGBTQ Caucus; Diversity Committee; and the SALUD Summit. |
| How do you gain buy-in from the organization and from whom ? | What organizational structures support developing resolutions or policies for members to vet and vote? As previously mentioned LMSA houses sub-groups including a LMSA-LGBTQ Caucus and Diversity Committee. In addition, LMSA hosts the SALUD Summit (formerly known as the LMSA Policy Summit), which serves as an opportunity for members to propose, vet, and vote on policies. The SALUD summit provides education on how to develop resolutions. |
| How do you gain buy-in from your medical school or external entities (NIH, Pharma, Biotech, HRSA, etc)? | Explore alignment with other organization’s mission statement or strategic plan. Explore committee that might have a similar identity. Explore if other organizations have a resolution or policy with similar content. For example, the National Institutes of Health Sexual & Gender Minority Research Office; HRSA National LGBTQIA+ Health Education Center; or GLMA Health Professionals Advancing LGBTQ+ Equality |
| What leadership development competencies could you develop through this project and why? | Examples:  Working with/developing others: bringing together individuals with and across organizations to draft a resolution.  Communication skills: explaining how a resolution aligns with organizational mission, vision, or strategic planning  Teambuilding: coalescing members to vote on a new resolution  Leadership: reflecting on the socio-political and organization’s culture to advance inclusion and equity for individuals who have a dual identity and associated unique health issues. |

Further information on this Sánchez JP, Sola O, Ramallo J, Sánchez NF, Dominguez K, Romero-Leggott V. Hispanic Medical Organizations Support for LGBT Health Issues. LGBT Health Volume 1, Number 3, 2014.

Sanchez JP and Rodriguez D. Latino, Hispanic, or of Spanish Origin+ Identified Students Leaders in Medicine. Recognizing More Than 50 Years of Presence, Activism and Leadership. Image by Springer Nature. Image is in the public domain. Creative Commons Attribution-NonCommercial-NoDerivatives 4.0 International License (<http://creativecommons.org/licenses/by-nc-nd/4.0/>).  Cover Page and Page 125 and 137.

**Slide 39 - Professional Journey Slide:**

Facilitator(s) should substitute content with their own personal/professional experiences with diversity-related, student-led NMOs (or their students) and discuss how that enabled them to develop the aforementioned leadership competencies and facilitated their career trajectory.

If the author’s journey slide is used, then discuss as follows:

**Slide 40:** *This is an example of one of the authors journey: Dennis J. Spencer, MD, PhD, Director, Equity Diversity and Inclusion, Division of Gastroenterology and Nutrition, Associate Program Director, Boston Combined Residency Program, Boston Children’s Hospital, Faculty Advisor, Office of Recruitment and Multicultural Affairs , Harvard Medical School*

Provides some points to review with learners.

**Slide 41:**

Summary slide: please review points with learners and provide your own insight/reflect on the presented material.

**Slide 42:**

Questions and answers.
