# Supplementary material for: Diversity-Related, Student-Led National Medical Organizations: Leadership Opportunities for Learners
Source: MedEdPORTAL. 2024 Dec 27;20:11477. doi: 10.15766/mep_2374-8265.11477 (PMC11671812; doi:10.15766/mep_2374-8265.11477)
Supplement: Supplementary file 1 — Facilitator Guide.docxPre- and Postworkshop Survey.docxNMOs Presentation.pptxExample SNMA Strategic Plan.docxNMOs Activities Handout.docxDr. Freeman SNMA Testimonial.mp4Fae MSPA Testimonial.mov [file mep_2374-8265.11477-s001.zip › B. Pre- and Postworkshop Survey.docx]

*The pre- and post- assessments are to be given at the beginning and end of the delivery of the module respectively. Please ensure that the pre-assessment is completed in its entirety before the start of the session.*

**Diversity-Related, Student-Led National Medical Organizations:
Leadership Development for Learners**

**Pre-Survey**

| How knowledgeable are you in identifying leadership opportunities for trainees to become engaged through Diversity-Related, Student-Led National Medical Organizations (circle one) | **Not Knowledgeable** | | **Somewhat Knowledgeable** | | **Knowledgeable** | | **Very knowledgeable** | |
| --- | --- | --- | --- | --- | --- | --- | --- | --- |
| How much CONFIDENCE do you have in your ability to… | **No Confidence**  **0** | **1** | | **2** | | **3** | | **Complete**  **Confidence**  **4** |
| Find a national medical organization aligned with your identity | 0 | 1 | | 2 | | 3 | | 4 |
| Work with national medical organizations to drive institutional change | 0 | 1 | | 2 | | 3 | | 4 |
| DEMOGRAPHICS: | | | | | | | | |
| In which STATE is your medical school or residency program located? _____________________ | | | | | | | | |
| Are you a (circle one):   1. Medical Student 2. Intern/Resident 3. Fellow 4. Other (please specify):_______________________________ | | | | | | | | |
| What is your race/ethnicity (circle all that apply)?   1. American Indian or Alaska Native 2. Native Hawaiian or Other Pacific Islander 3. Asian 4. Black or African-American 5. Latina/o/x/e, Hispanic or of Spanish Origin+ (LHS+) 6. White 7. Other (please specify): | | | | | | | | |
| How do you self-identify (circle one)?   1. Straight or Heterosexual 2. Gay or Lesbian 3. Bisexual 4. Other (please specify): ____________________ | | | | | | | | |
| How do you self-identify? Note: Respondents who self-identify as “Transgender female,” “Transgender male,” or “Transgender do not identify as exclusively male or female” are combined and displayed as “Transgender” (circle all that apply):   1. Male 2. Female 3. Transgender 4. Other: ________________________________ | | | | | | | | |

**Post-Survey**

| Unique Identifier |  | | | | | | | | |
| --- | --- | --- | --- | --- | --- | --- | --- | --- | --- |
| How much CONFIDENCE do you have in your ability to… | **No Confidence**  **0** | **1** | | | **2** | | **3** | | **Complete**  **Confidence**  **4** |
| Find a national medical organization aligned with your identity | 0 | 1 | | | 2 | | 3 | | 4 |
| Work with national medical organizations to drive institutional change | 0 | 1 | | | 2 | | 3 | | 4 |
| To what EXTENT do you agree that the workshop learning objectives were met | **Strongly agree** | | **Agree** | **Neither agree or disagree** | | **Disagree** | | **Strongly disagree** | |
| Describe the role of diversity-related, student-led national medical organizations in developing future diverse leaders | SA | | A | N | | D | | SD | |
| Describe engagement and leadership opportunities for trainees through diversity-related, student-led NMO; | SA | | A | N | | D | | SD | |
| Review examples of successful trainee-driven activities and leadership competencies gaine*d* through diversity-related student-led NMO | SA | | A | N | | D | | SD | |
| Highlight the experiences of and competencies gained by members of diversity-related student-led NMO | SA | | A | N | | D | | SD | |
| What did you like about this workshop? | | | | | | | | | |
| What suggestions do you have to improve this workshop? | | | | | | | | | |
