# Supplementary material for: Diversity-Related, Student-Led National Medical Organizations: Leadership Opportunities for Learners
Source: MedEdPORTAL. 2024 Dec 27;20:11477. doi: 10.15766/mep_2374-8265.11477 (PMC11671812; doi:10.15766/mep_2374-8265.11477)
Supplement: Supplementary file 1 — Facilitator Guide.docxPre- and Postworkshop Survey.docxNMOs Presentation.pptxExample SNMA Strategic Plan.docxNMOs Activities Handout.docxDr. Freeman SNMA Testimonial.mp4Fae MSPA Testimonial.mov [file mep_2374-8265.11477-s001.zip › C. NMOs Presentation.pptx]

## Slide 1
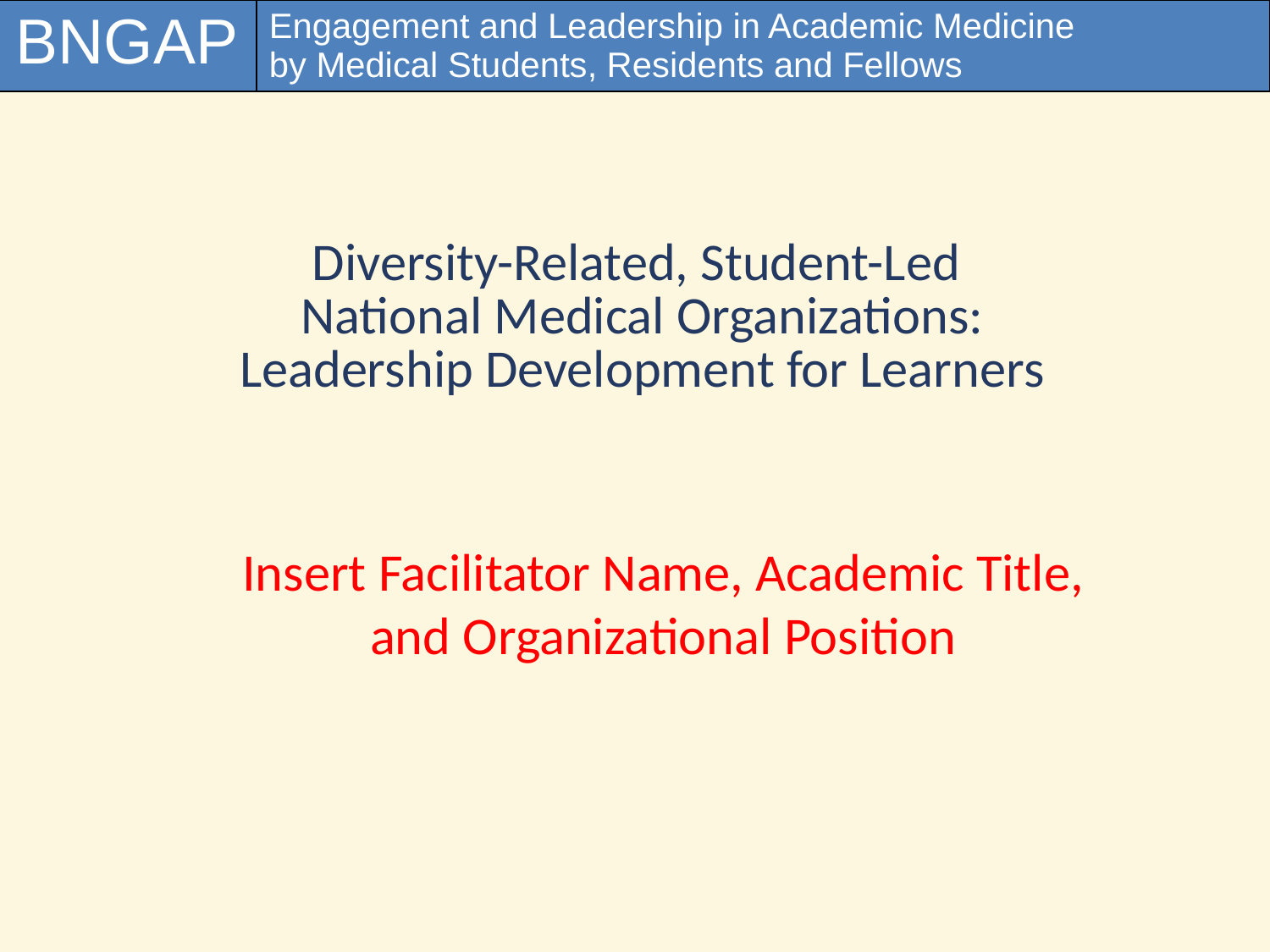

# Diversity-Related, Student-Led National Medical Organizations: Leadership Development for Learners
Insert Facilitator Name, Academic Title,
and Organizational Position

## Slide 2
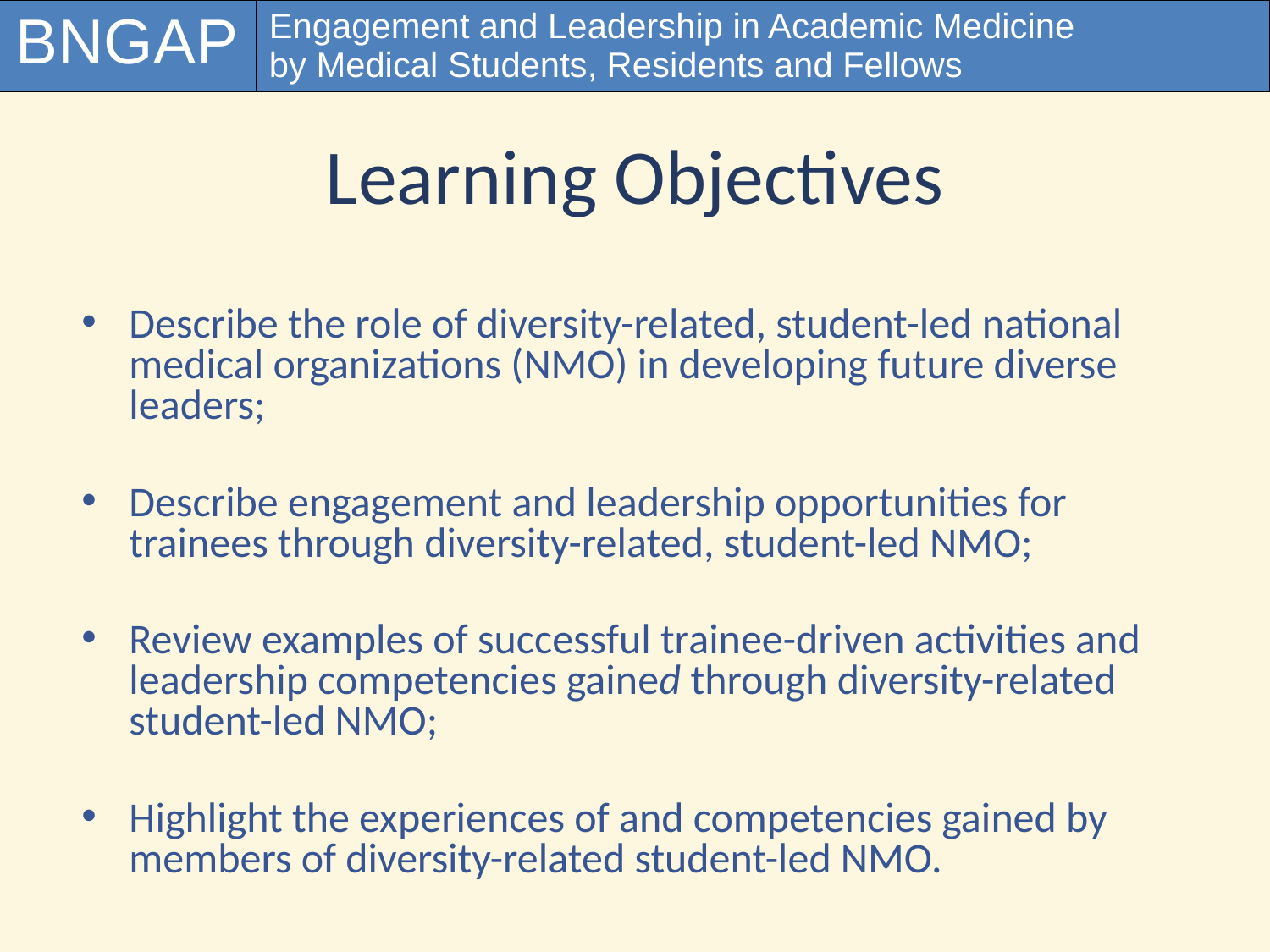

# Learning Objectives
Describe the role of diversity-related, student-led national medical organizations (NMO) in developing future diverse leaders;
Describe engagement and leadership opportunities for trainees through diversity-related, student-led NMO;
Review examples of successful trainee-driven activities and leadership competencies gained through diversity-related student-led NMO;
Highlight the experiences of and competencies gained by members of diversity-related student-led NMO.

## Slide 3
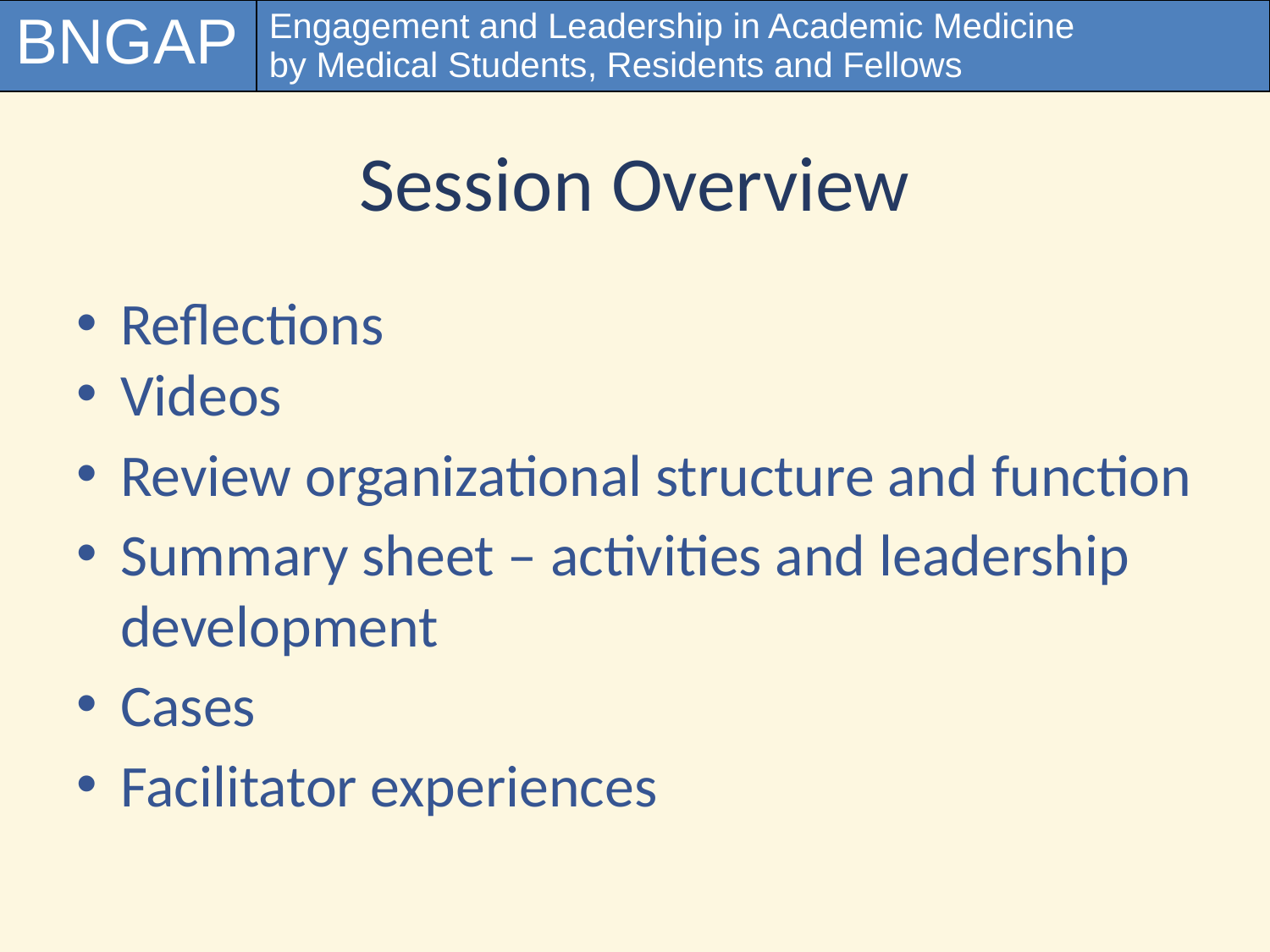

# Session Overview
Reflections
Videos
Review organizational structure and function
Summary sheet – activities and leadership development
Cases
Facilitator experiences

## Slide 4
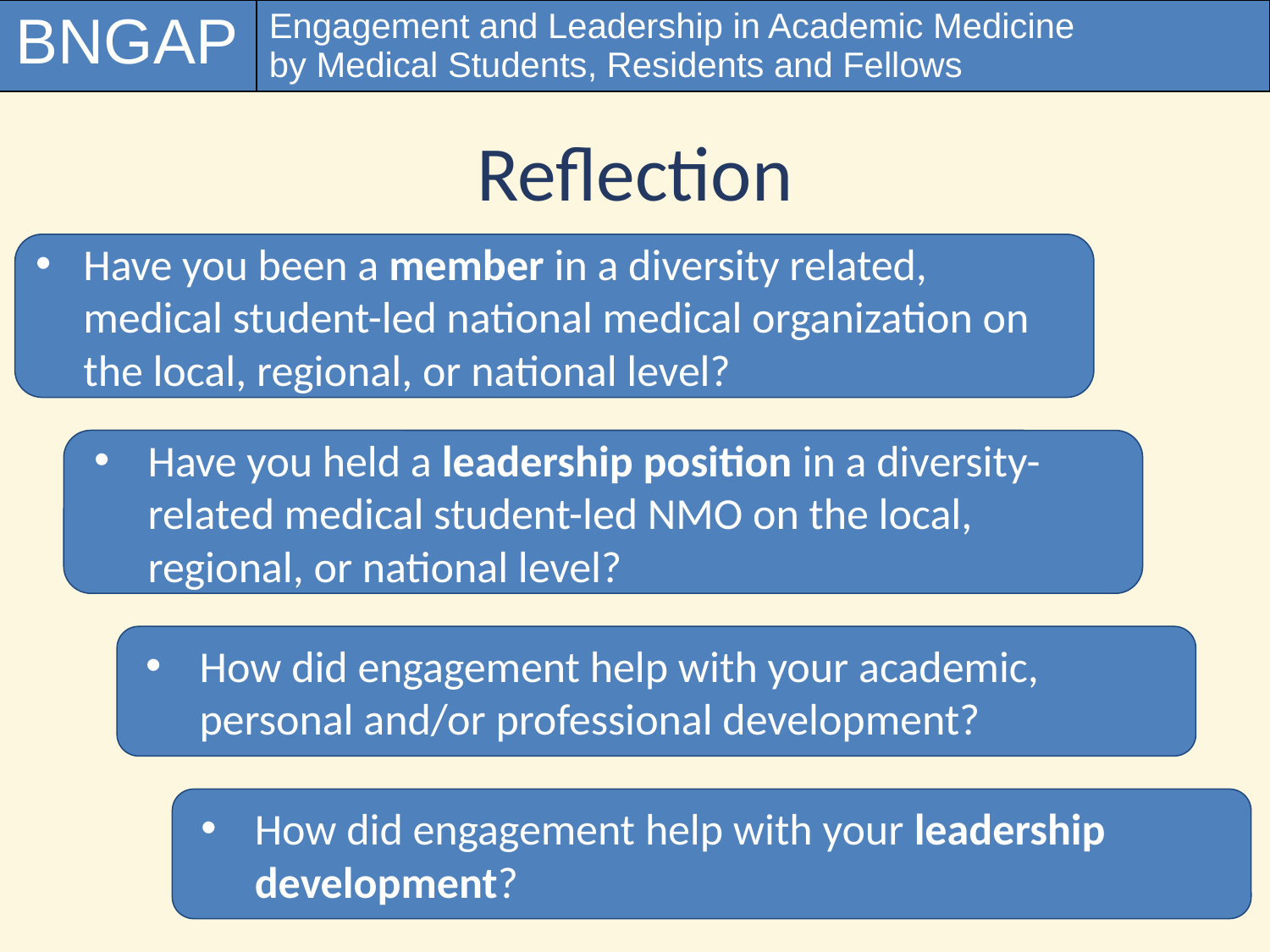

# Reflection
Have you been a member in a diversity related, medical student-led national medical organization on the local, regional, or national level?
Have you held a leadership position in a diversity- related medical student-led NMO on the local, regional, or national level?
How did engagement help with your academic, personal and/or professional development?
How did engagement help with your leadership development?

## Slide 5
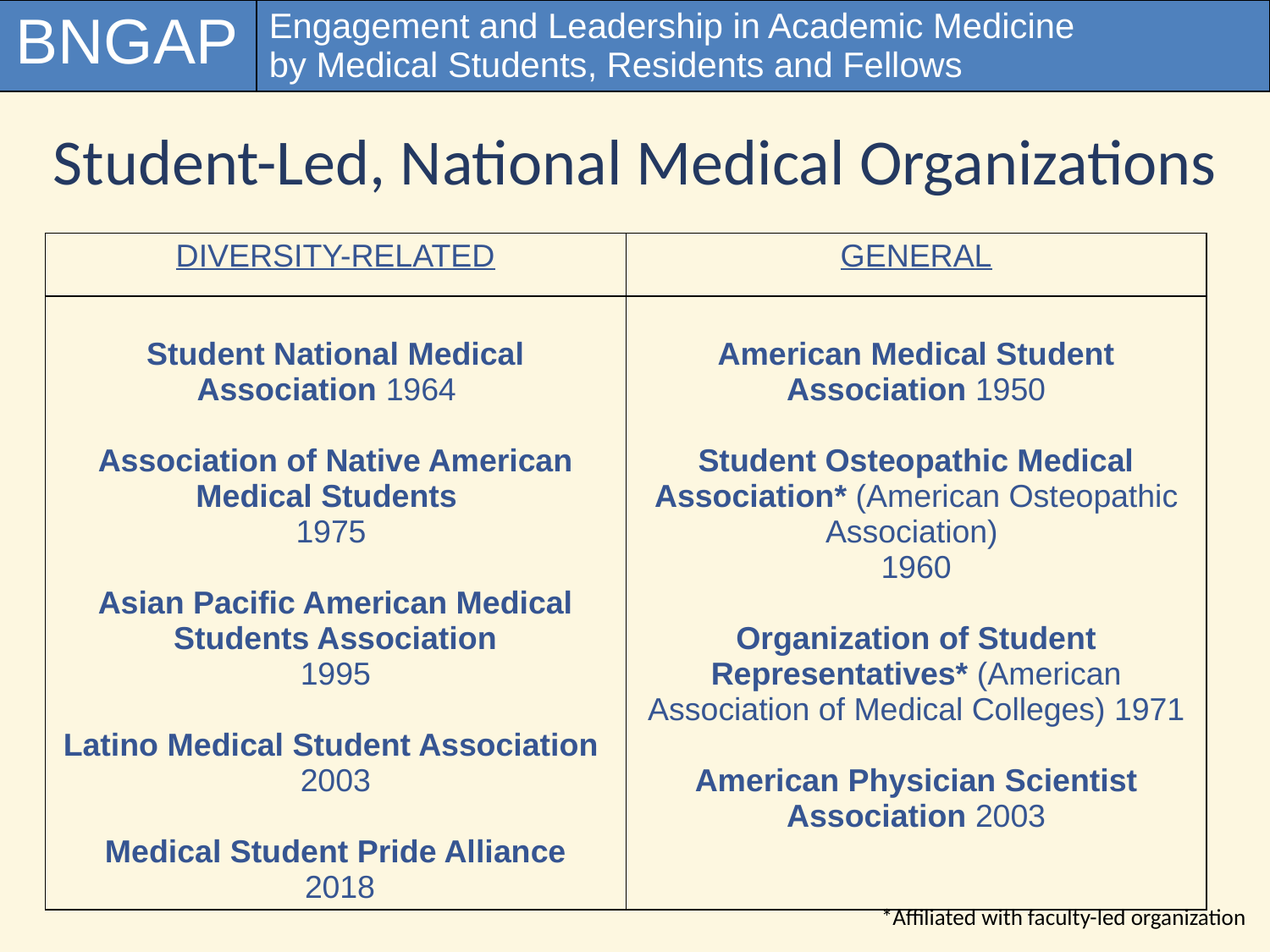

# Student-Led, National Medical Organizations
| DIVERSITY-RELATED | GENERAL |
| --- | --- |
| Student National Medical Association 1964 Association of Native American Medical Students 1975 Asian Pacific American Medical Students Association 1995 Latino Medical Student Association 2003 Medical Student Pride Alliance 2018 | American Medical Student Association 1950 Student Osteopathic Medical Association\* (American Osteopathic Association) 1960 Organization of Student Representatives\* (American Association of Medical Colleges) 1971 American Physician Scientist Association 2003 |
*Affiliated with faculty-led organization

## Slide 6
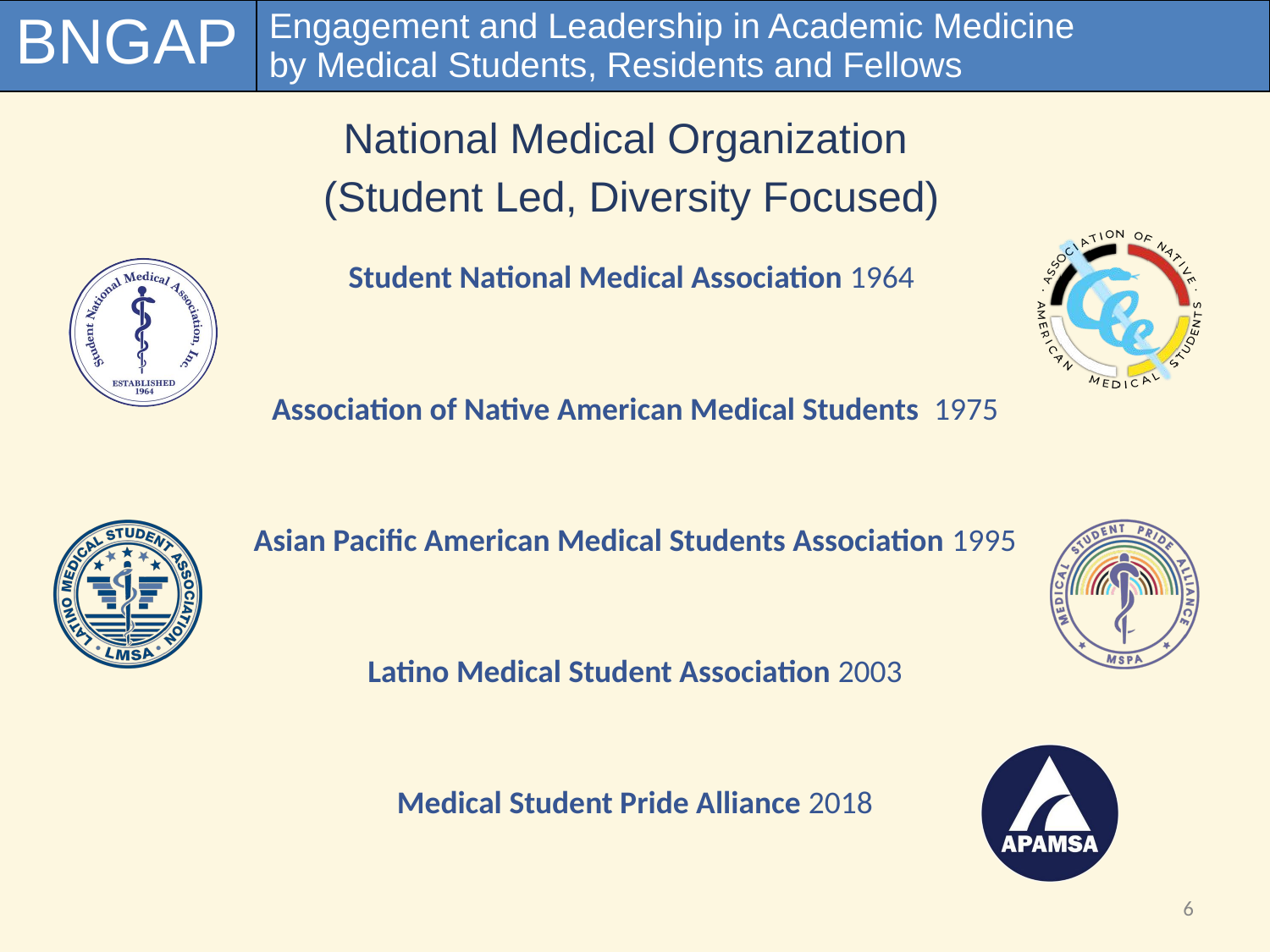

# National Medical Organization
(Student Led, Diversity Focused)
Student National Medical Association 1964
Association of Native American Medical Students 1975
Asian Pacific American Medical Students Association 1995
Latino Medical Student Association 2003
Medical Student Pride Alliance 2018
6

## Slide 7
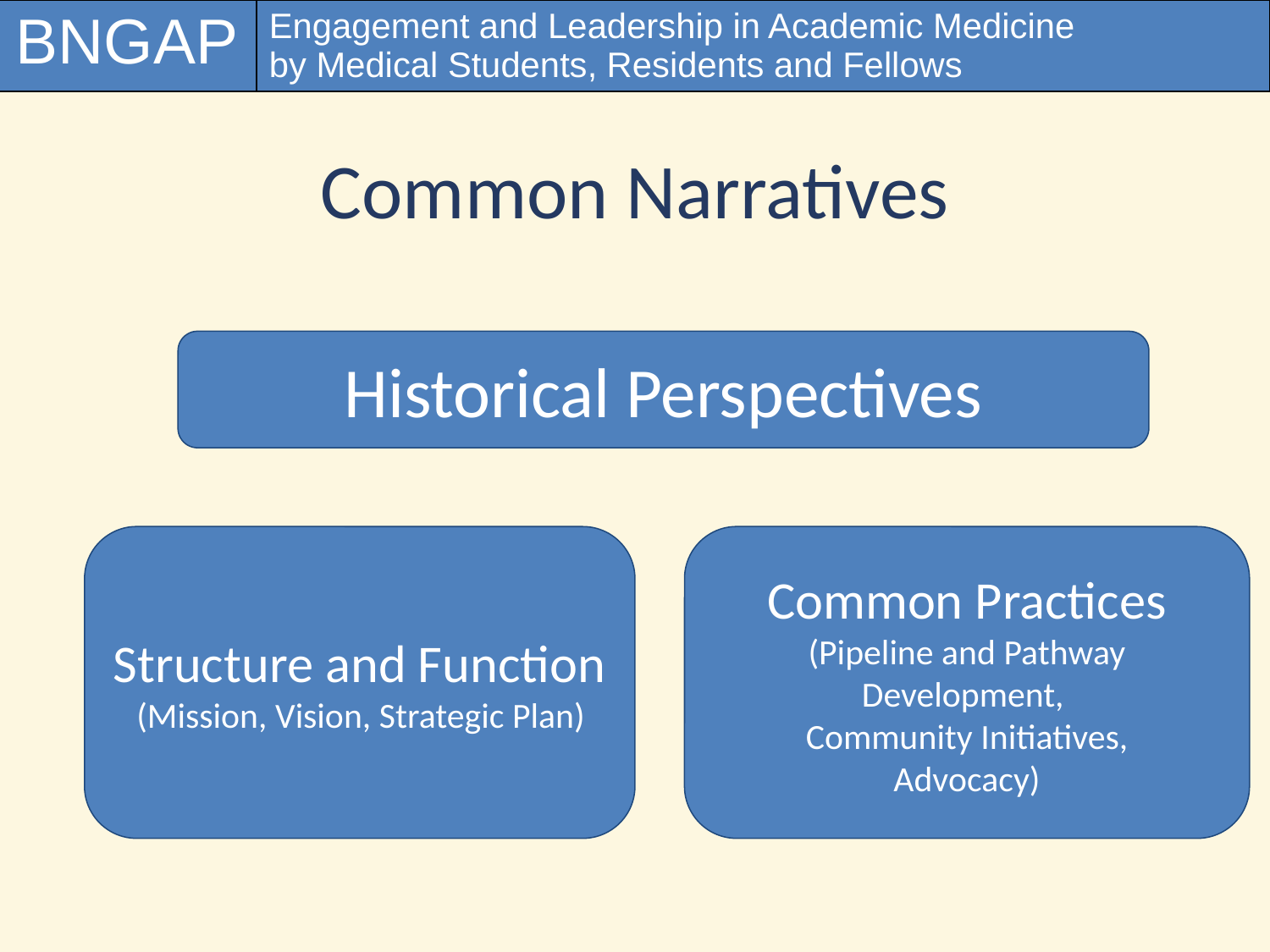

# Common Narratives
Historical Perspectives
Structure and Function
 (Mission, Vision, Strategic Plan)
Common Practices
(Pipeline and Pathway Development,
Community Initiatives,
Advocacy)

## Slide 8
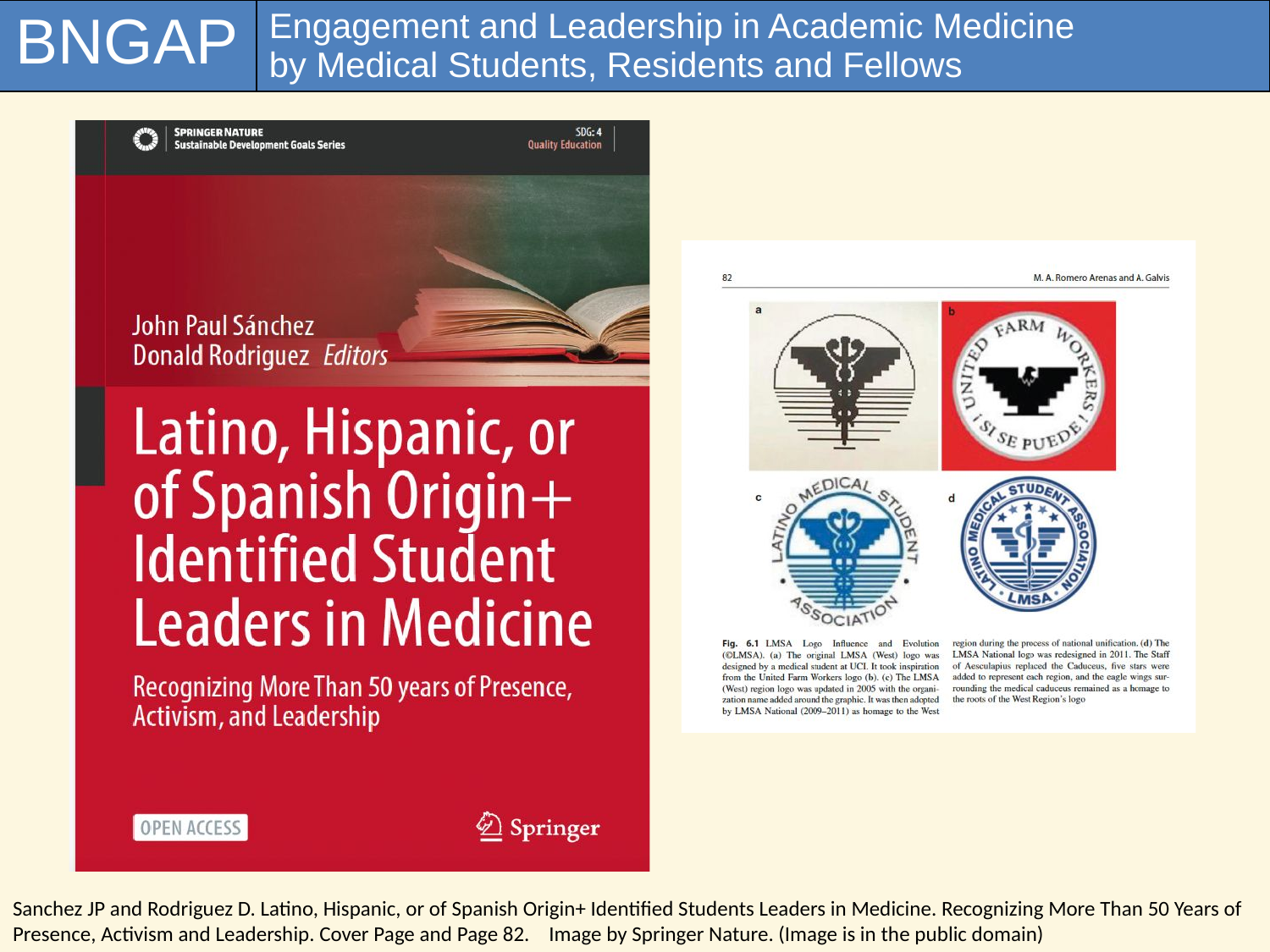

Sanchez JP and Rodriguez D. Latino, Hispanic, or of Spanish Origin+ Identified Students Leaders in Medicine. Recognizing More Than 50 Years of Presence, Activism and Leadership. Cover Page and Page 82. Image by Springer Nature. (Image is in the public domain)

## Slide 9
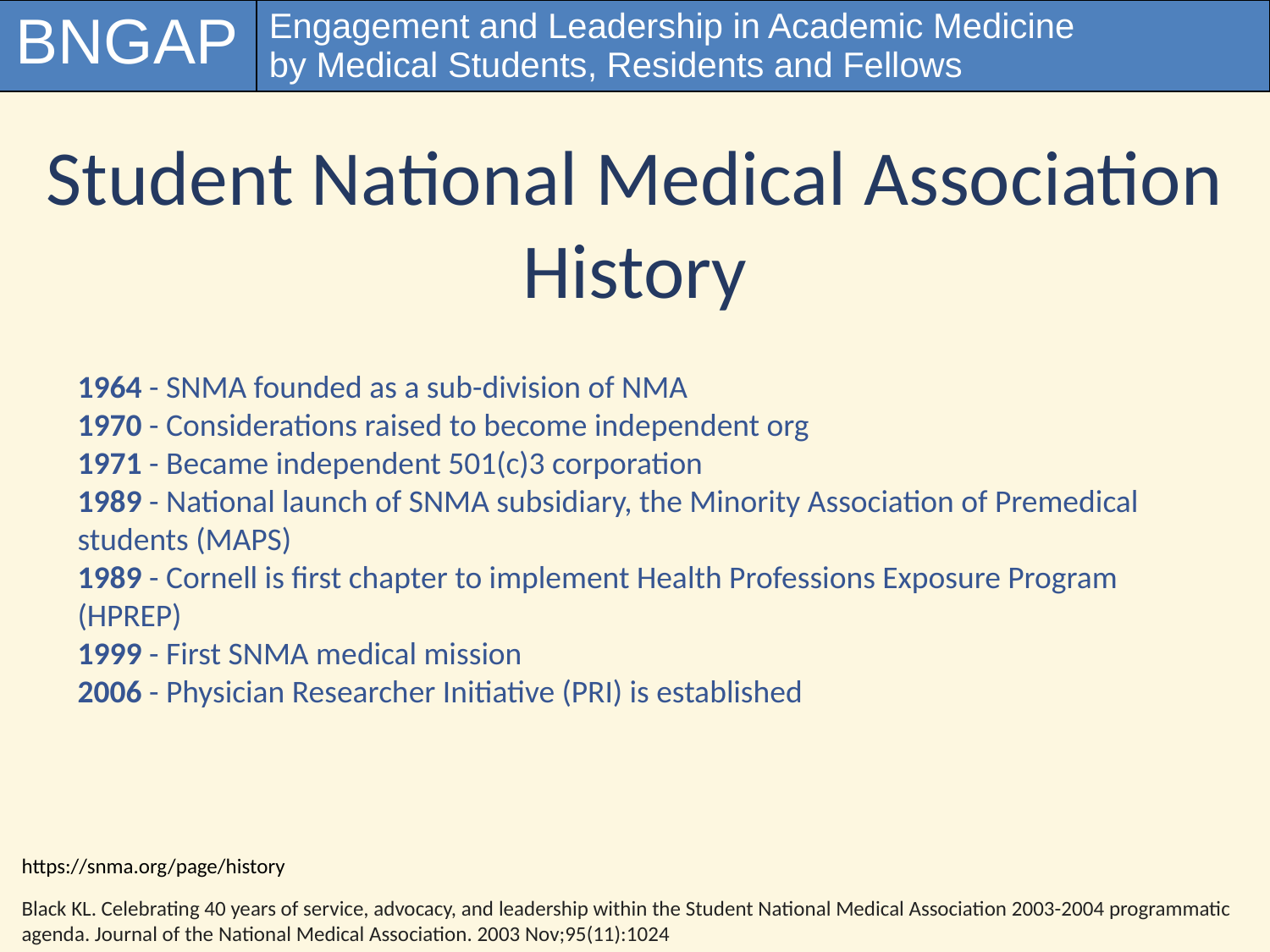

Student National Medical Association History
1964 - SNMA founded as a sub-division of NMA
1970 - Considerations raised to become independent org
1971 - Became independent 501(c)3 corporation
1989 - National launch of SNMA subsidiary, the Minority Association of Premedical students (MAPS)
1989 - Cornell is first chapter to implement Health Professions Exposure Program (HPREP)
1999 - First SNMA medical mission
2006 - Physician Researcher Initiative (PRI) is established
https://snma.org/page/history
Black KL. Celebrating 40 years of service, advocacy, and leadership within the Student National Medical Association 2003-2004 programmatic agenda. Journal of the National Medical Association. 2003 Nov;95(11):1024

## Slide 10
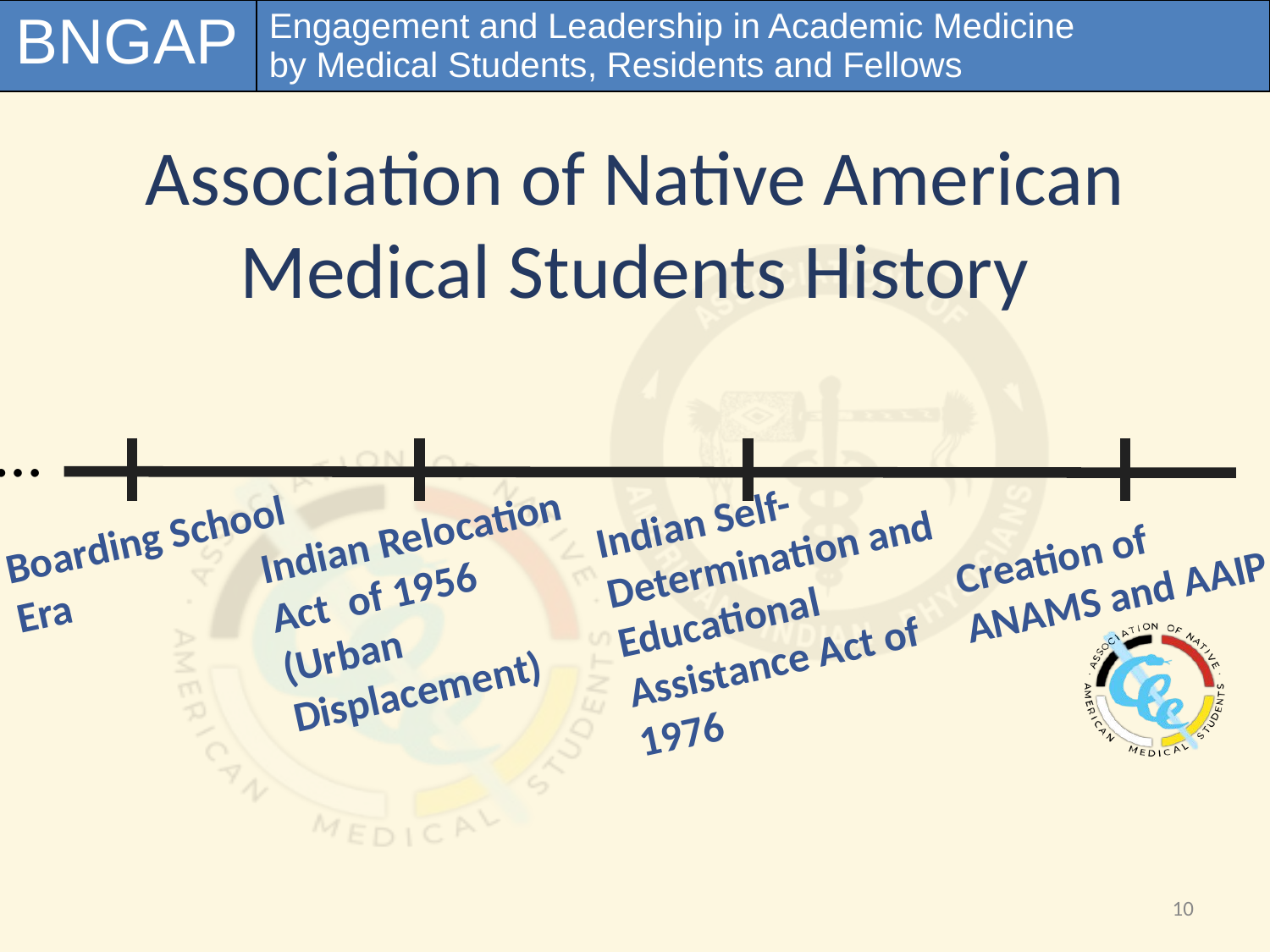

Association of Native American Medical Students History
…
Indian Self-Determination and Educational Assistance Act of 1976
Indian Relocation Act of 1956 (Urban Displacement)
Boarding School Era
Creation of ANAMS and AAIP
10

## Slide 11
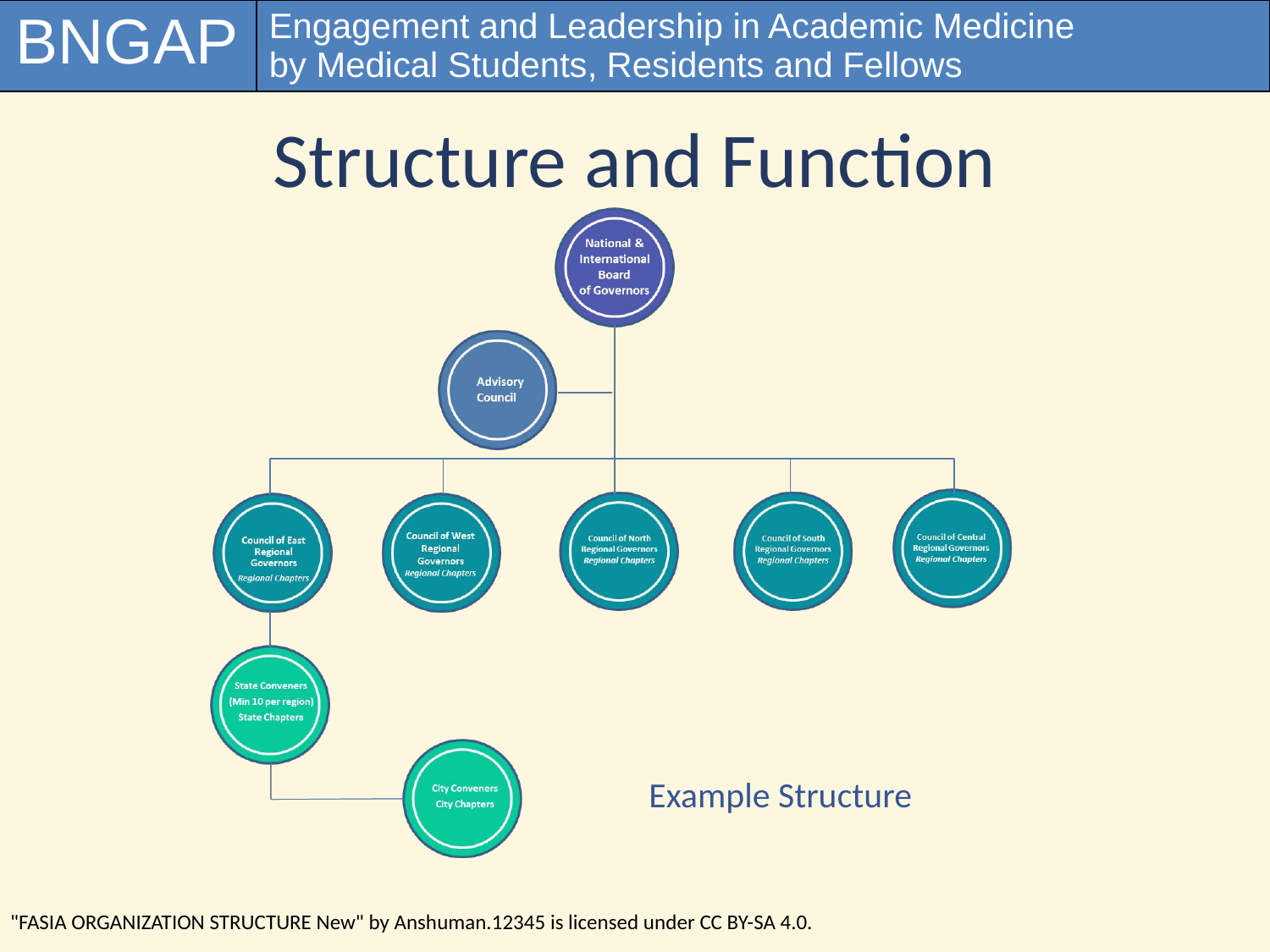

# Structure and Function
Example Structure
"FASIA ORGANIZATION STRUCTURE New" by Anshuman.12345 is licensed under CC BY-SA 4.0.

## Slide 12
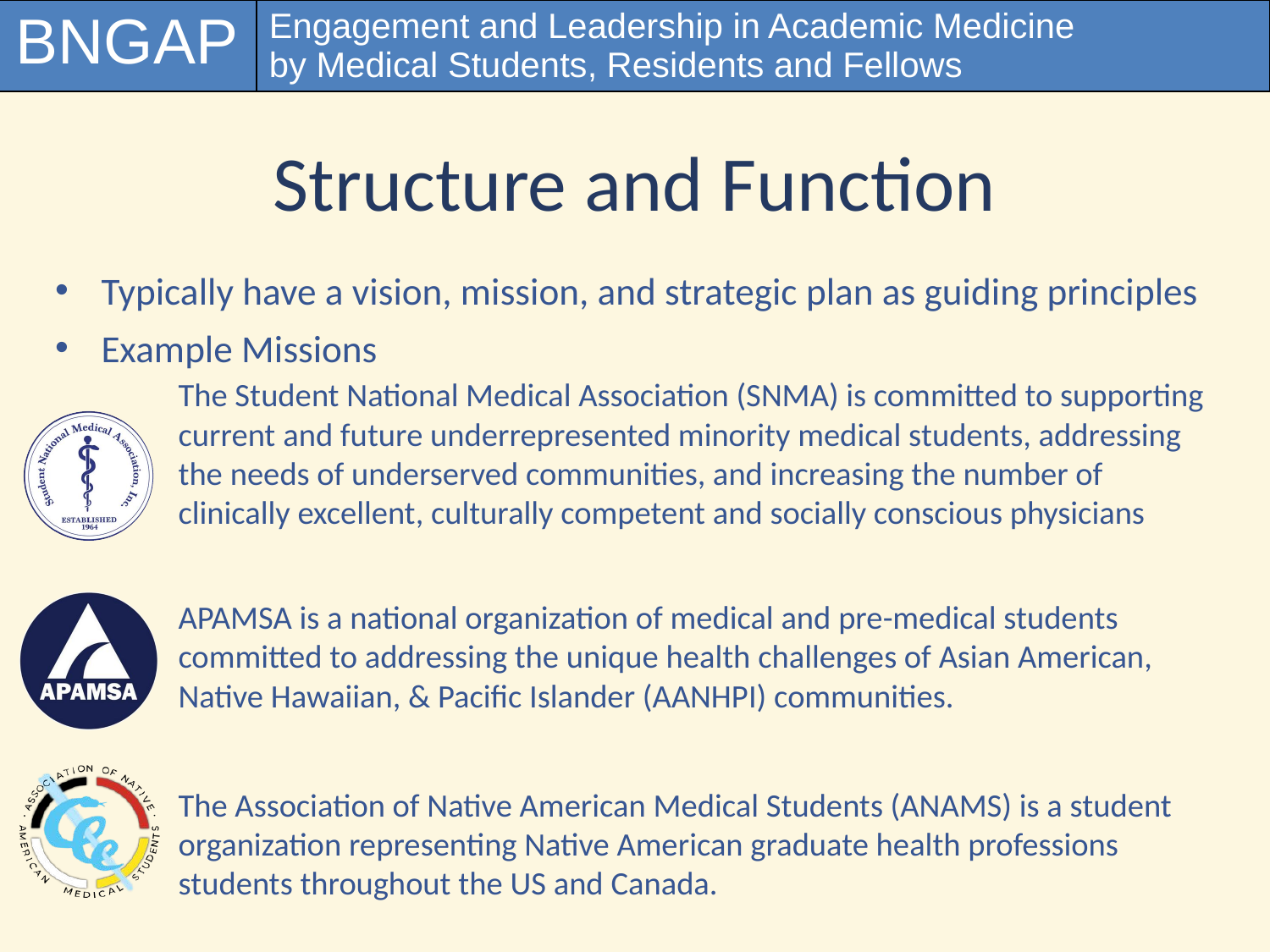

# Structure and Function
Typically have a vision, mission, and strategic plan as guiding principles
Example Missions
The Student National Medical Association (SNMA) is committed to supporting current and future underrepresented minority medical students, addressing the needs of underserved communities, and increasing the number of clinically excellent, culturally competent and socially conscious physicians
APAMSA is a national organization of medical and pre-medical students committed to addressing the unique health challenges of Asian American, Native Hawaiian, & Pacific Islander (AANHPI) communities.
The Association of Native American Medical Students (ANAMS) is a student organization representing Native American graduate health professions students throughout the US and Canada.

## Slide 13
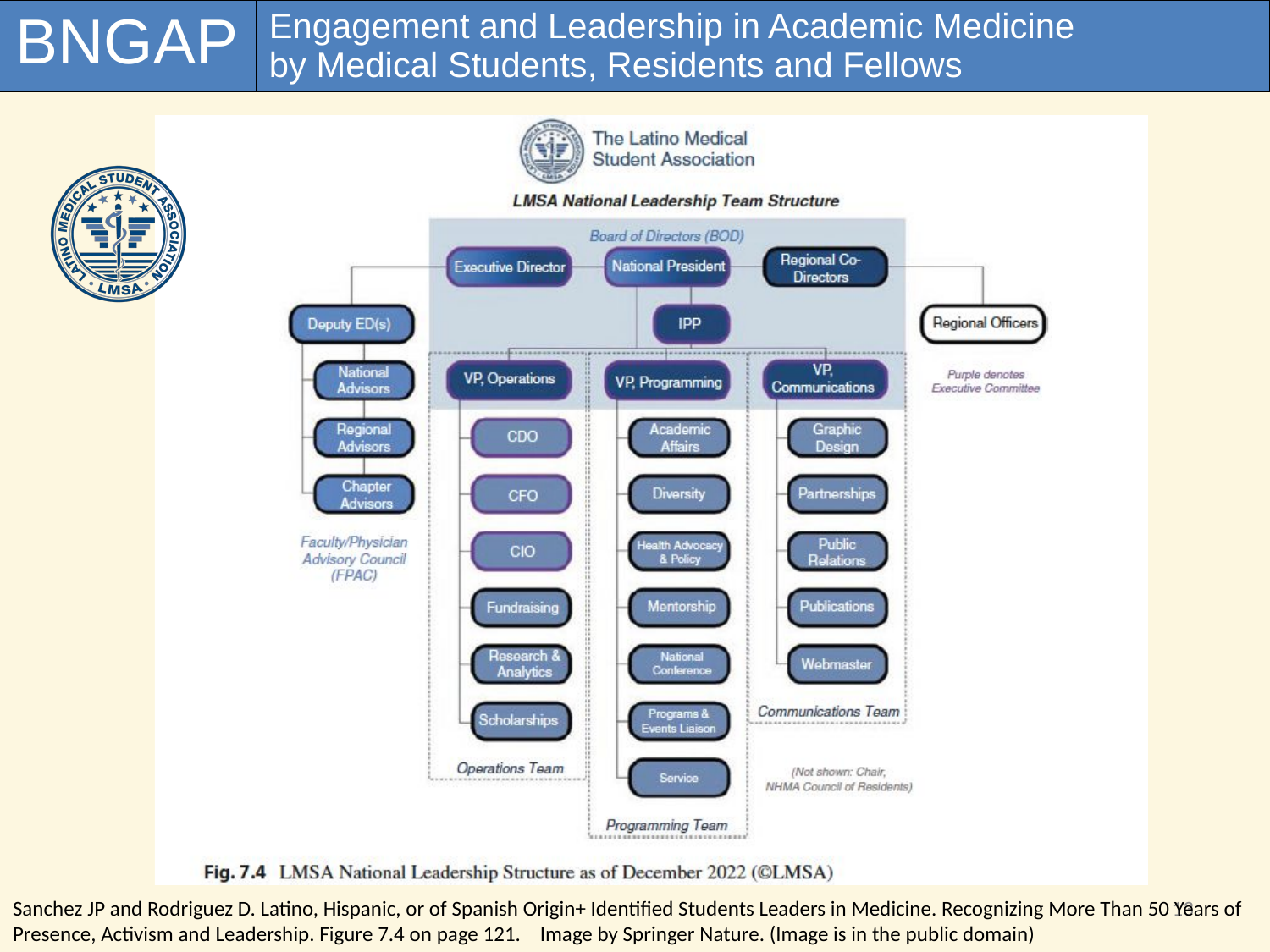

Sanchez JP and Rodriguez D. Latino, Hispanic, or of Spanish Origin+ Identified Students Leaders in Medicine. Recognizing More Than 50 Years of Presence, Activism and Leadership. Figure 7.4 on page 121. Image by Springer Nature. (Image is in the public domain)
13

## Slide 14
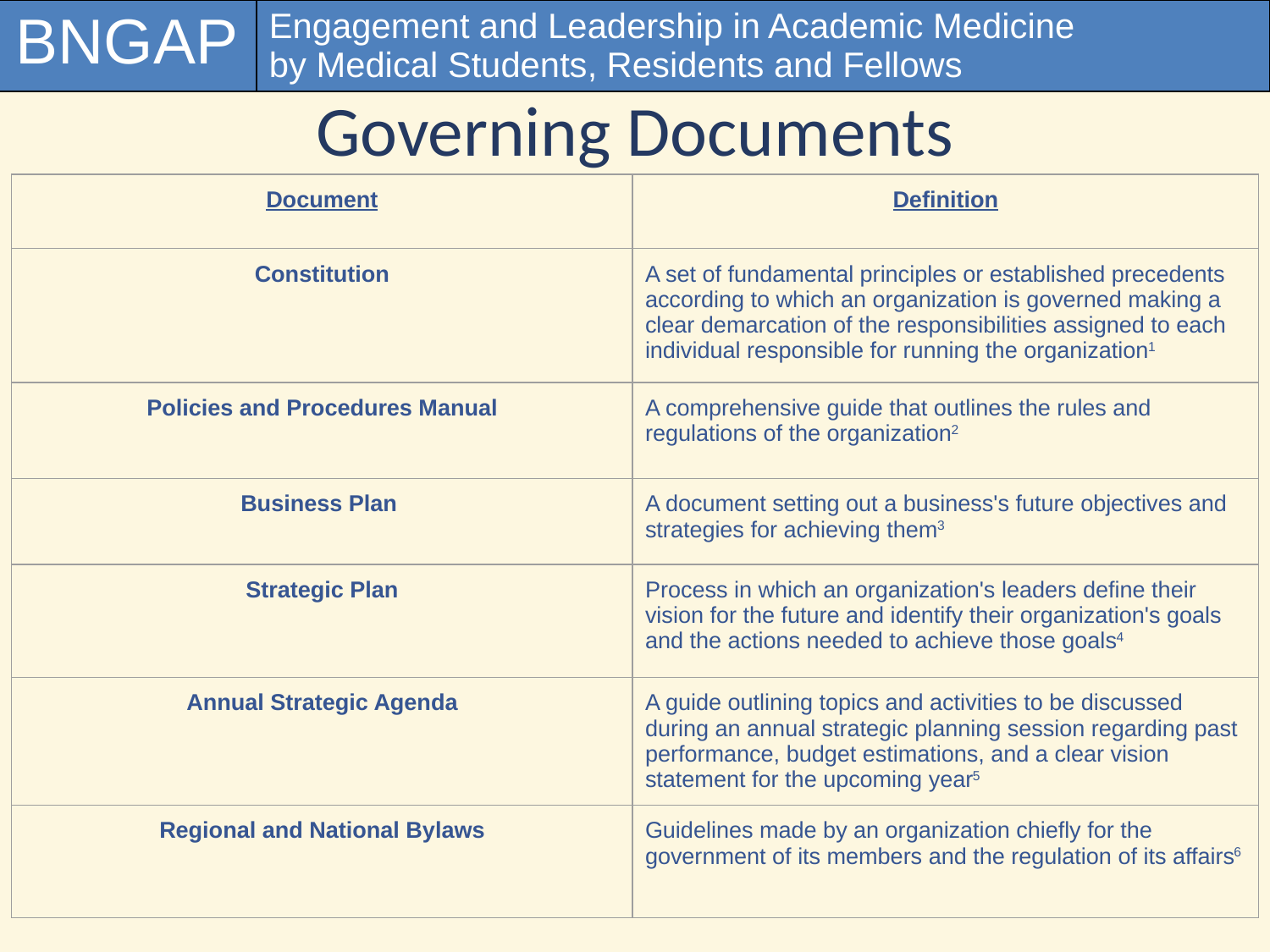

# Governing Documents
| Document | Definition |
| --- | --- |
| Constitution | A set of fundamental principles or established precedents according to which an organization is governed making a clear demarcation of the responsibilities assigned to each individual responsible for running the organization1 |
| Policies and Procedures Manual | A comprehensive guide that outlines the rules and regulations of the organization2 |
| Business Plan | A document setting out a business's future objectives and strategies for achieving them3 |
| Strategic Plan | Process in which an organization's leaders define their vision for the future and identify their organization's goals and the actions needed to achieve those goals4 |
| Annual Strategic Agenda | A guide outlining topics and activities to be discussed during an annual strategic planning session regarding past performance, budget estimations, and a clear vision statement for the upcoming year5 |
| Regional and National Bylaws | Guidelines made by an organization chiefly for the government of its members and the regulation of its affairs6 |

## Slide 15
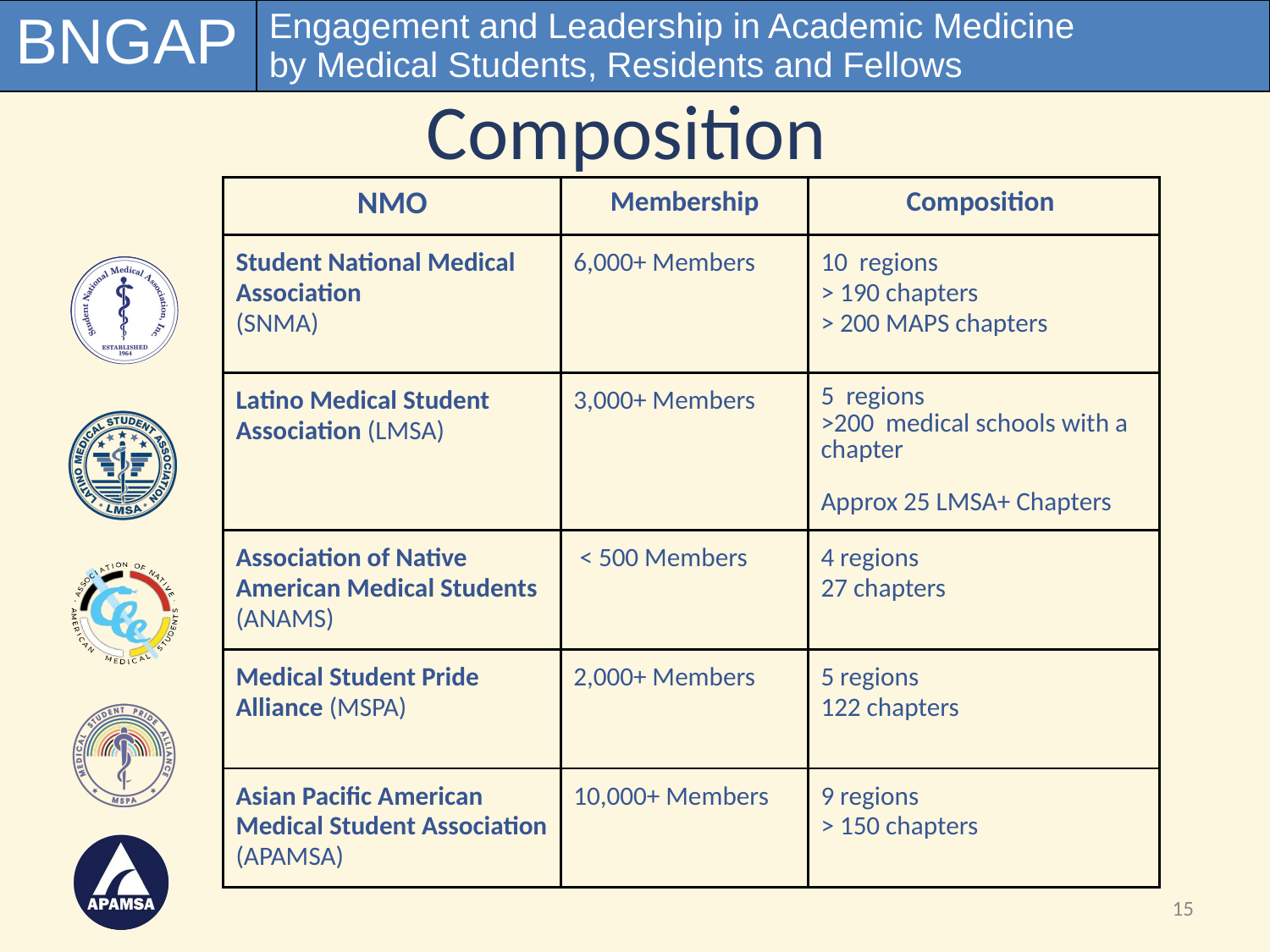

Composition
| NMO | Membership | Composition |
| --- | --- | --- |
| Student National Medical Association (SNMA) | 6,000+ Members | 10 regions > 190 chapters > 200 MAPS chapters |
| Latino Medical Student Association (LMSA) | 3,000+ Members | 5 regions >200 medical schools with a chapter Approx 25 LMSA+ Chapters |
| Association of Native American Medical Students (ANAMS) | < 500 Members | 4 regions 27 chapters |
| Medical Student Pride Alliance (MSPA) | 2,000+ Members | 5 regions 122 chapters |
| Asian Pacific American Medical Student Association (APAMSA) | 10,000+ Members | 9 regions > 150 chapters |
15

## Slide 16
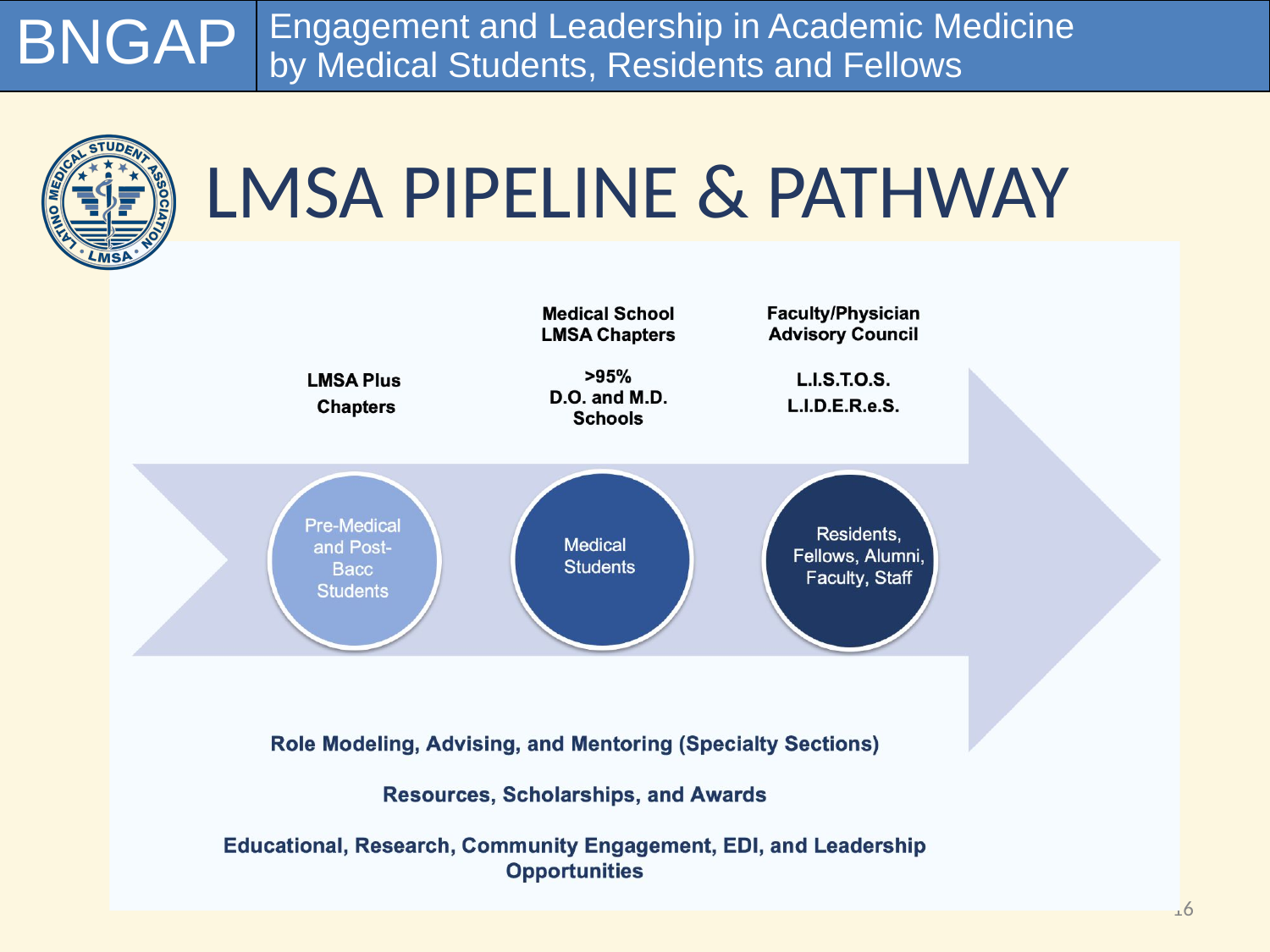

LMSA PIPELINE & PATHWAY
16

## Slide 17
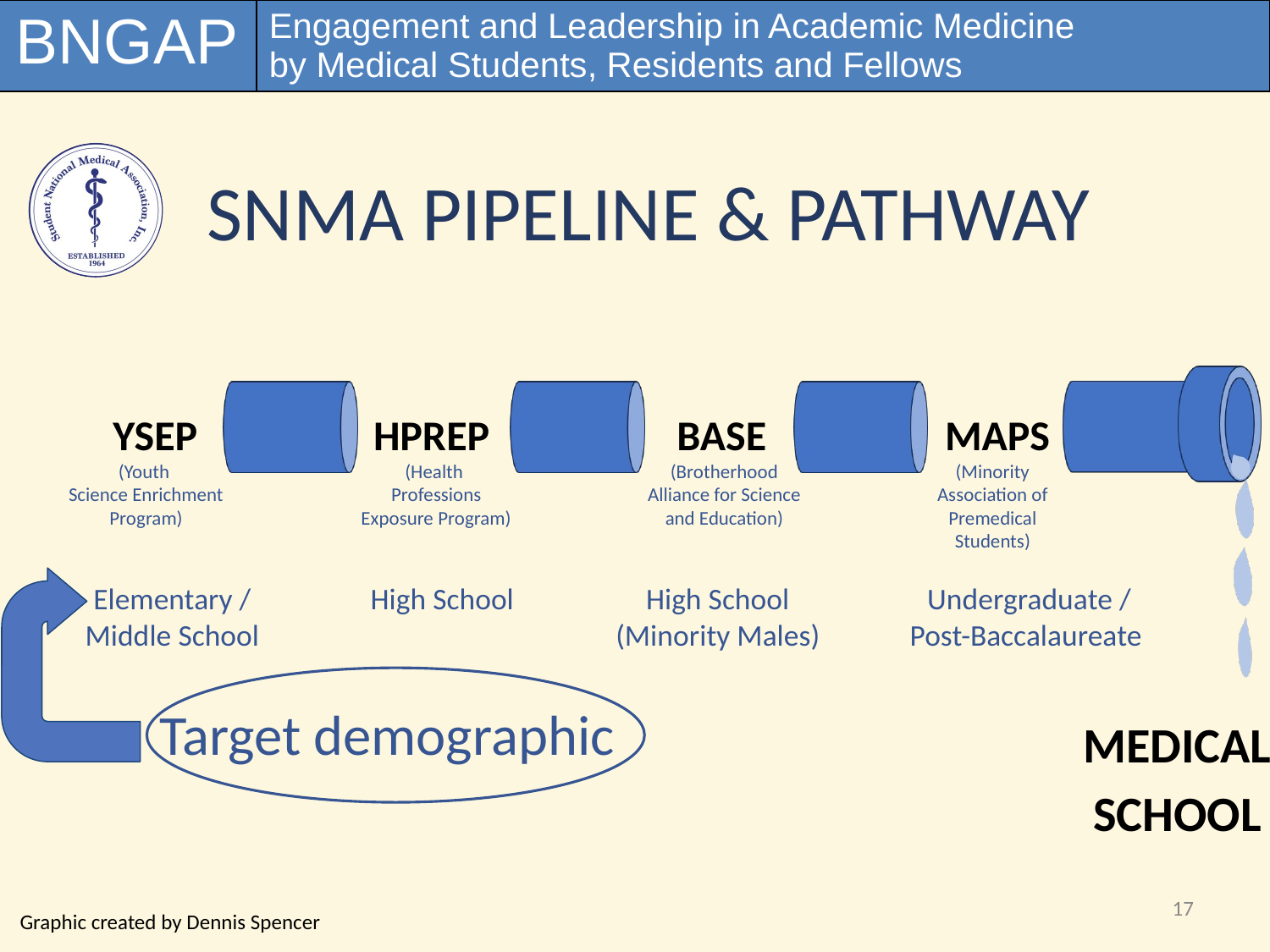

SNMA PIPELINE & PATHWAY
YSEP
HPREP
BASE
MAPS
(Health
Professions Exposure Program)
(Youth
Science Enrichment Program)
(Brotherhood Alliance for Science and Education)
(Minority Association of Premedical Students)
Elementary / Middle School
High School
High School (Minority Males)
Undergraduate / Post-Baccalaureate
Target demographic
MEDICAL SCHOOL
17
Graphic created by Dennis Spencer

## Slide 18
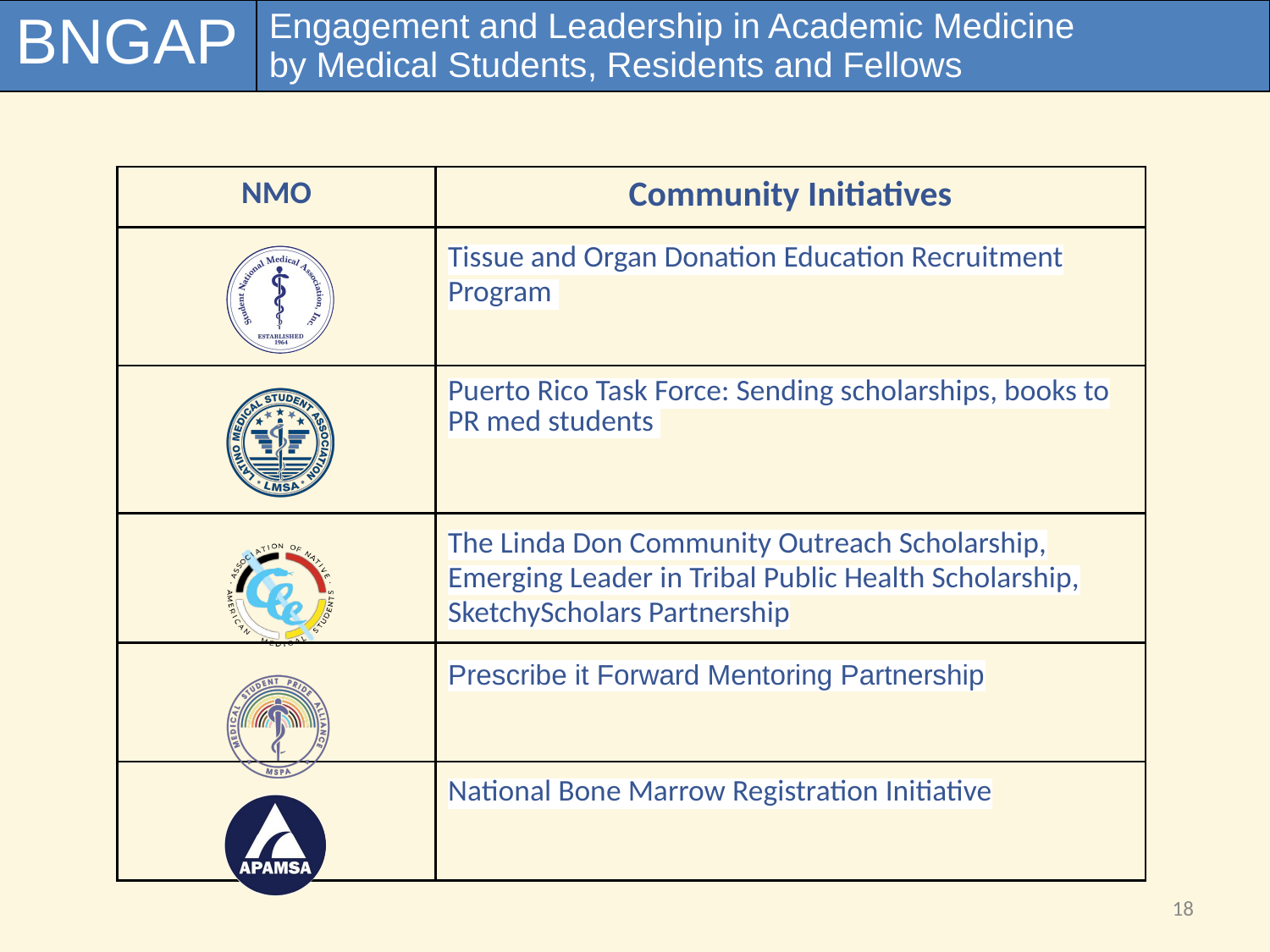

| NMO | Community Initiatives |
| --- | --- |
| | Tissue and Organ Donation Education Recruitment Program |
| | Puerto Rico Task Force: Sending scholarships, books to PR med students |
| | The Linda Don Community Outreach Scholarship, Emerging Leader in Tribal Public Health Scholarship, SketchyScholars Partnership |
| | Prescribe it Forward Mentoring Partnership |
| | National Bone Marrow Registration Initiative |
18

## Slide 19
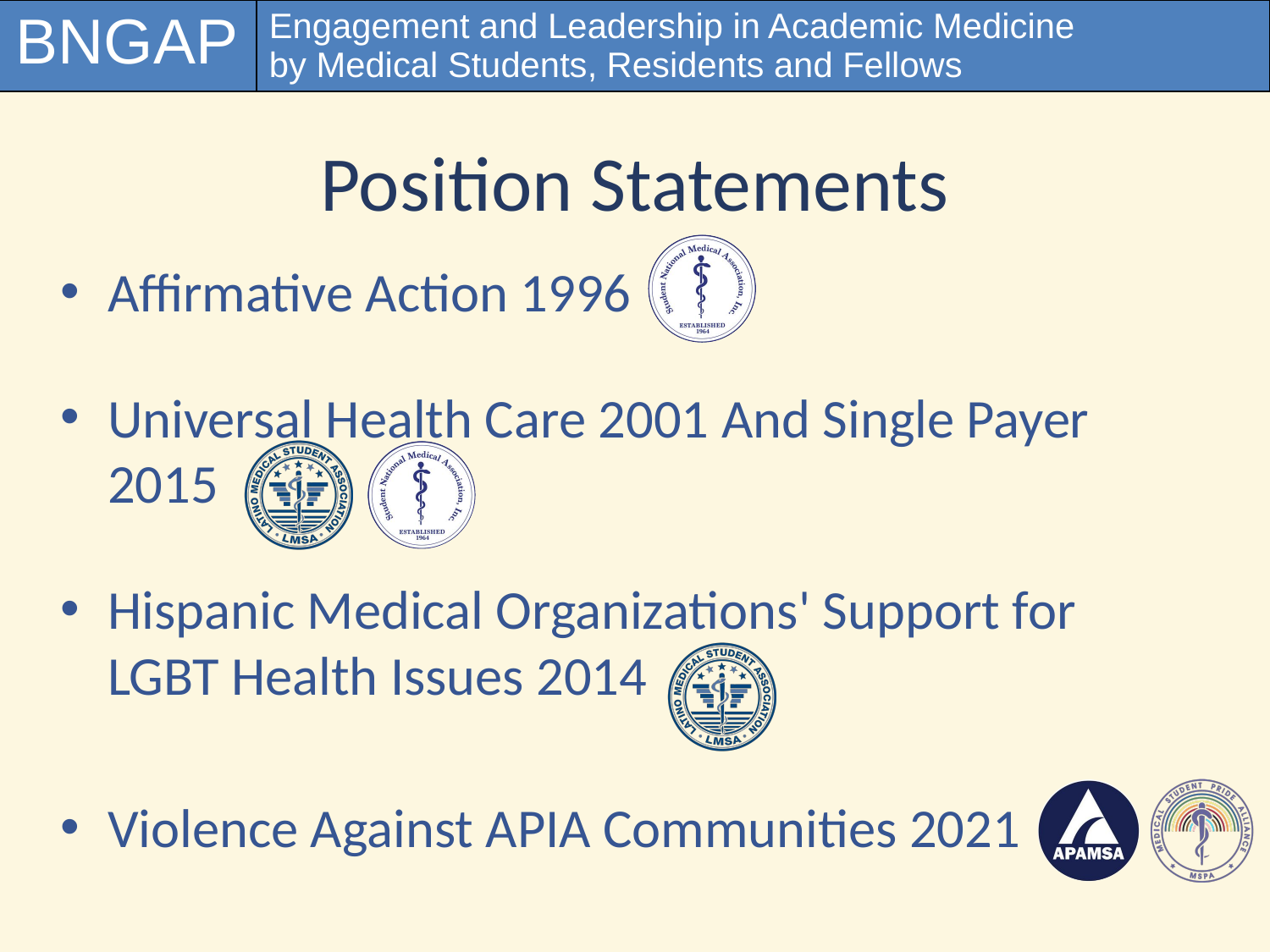

# Position Statements
Affirmative Action 1996
Universal Health Care 2001 And Single Payer 2015
Hispanic Medical Organizations' Support for LGBT Health Issues 2014
Violence Against APIA Communities 2021

## Slide 20
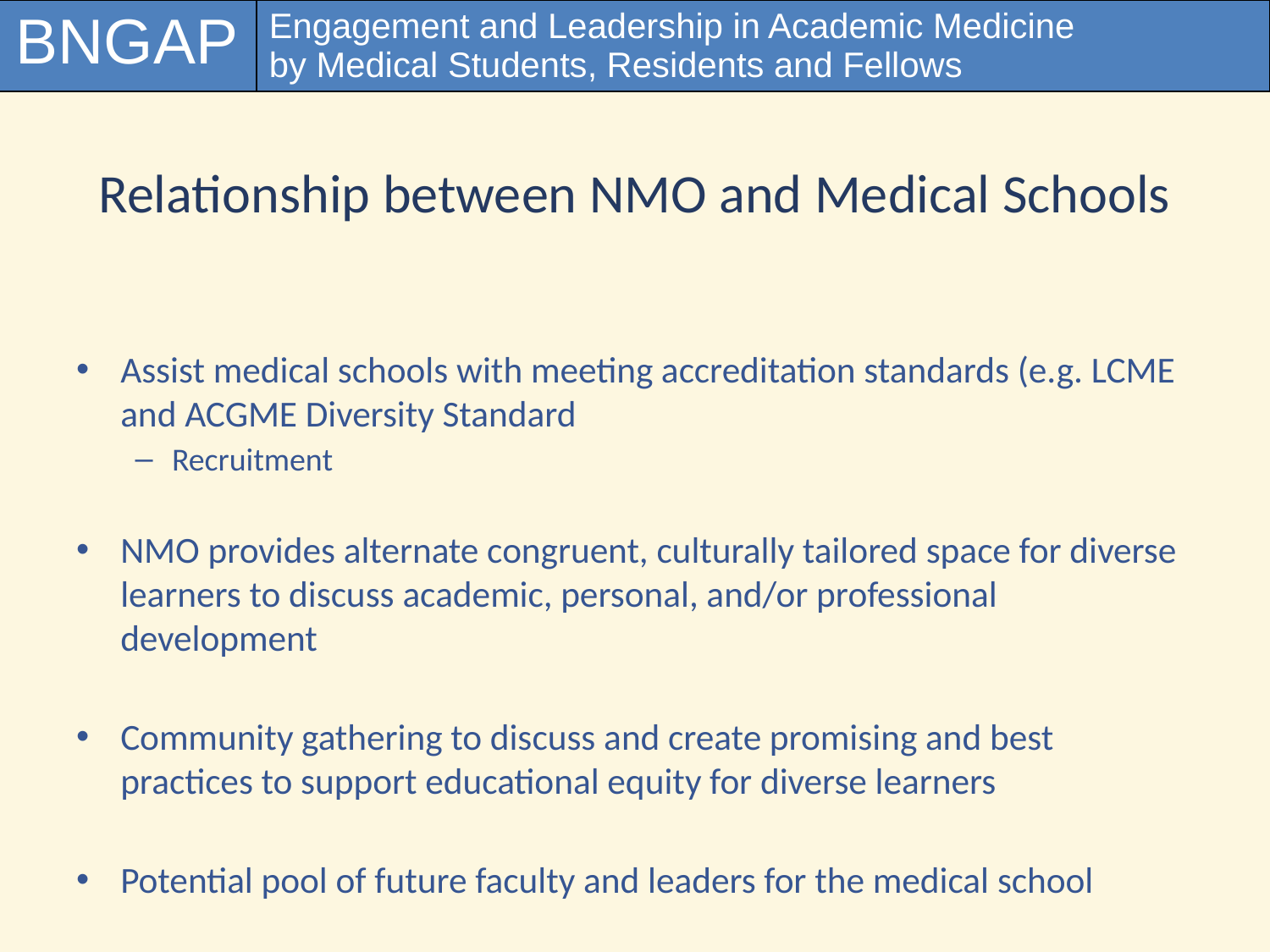

# Relationship between NMO and Medical Schools
Assist medical schools with meeting accreditation standards (e.g. LCME and ACGME Diversity Standard
Recruitment
NMO provides alternate congruent, culturally tailored space for diverse learners to discuss academic, personal, and/or professional development
Community gathering to discuss and create promising and best practices to support educational equity for diverse learners
Potential pool of future faculty and leaders for the medical school

## Slide 21
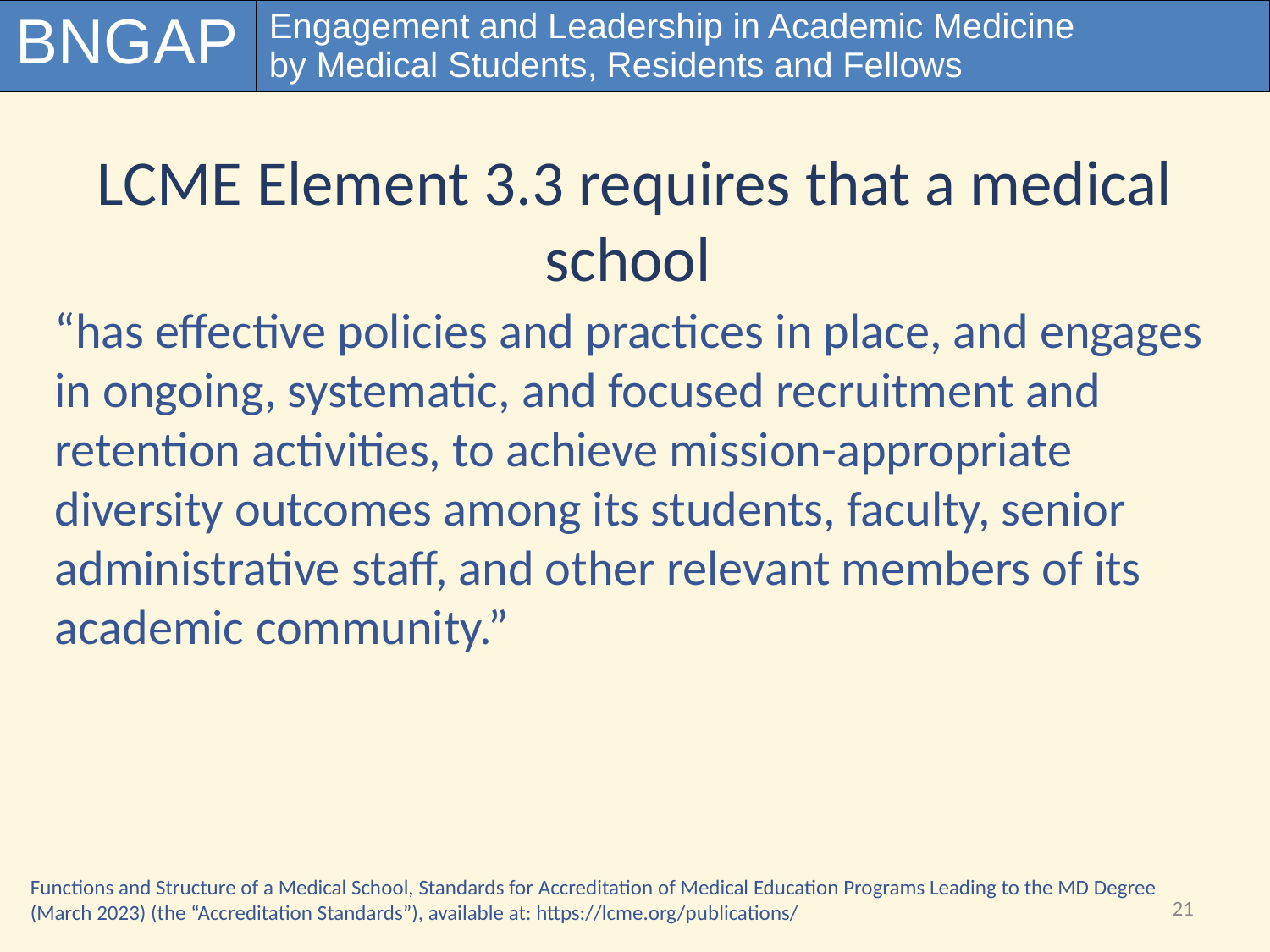

LCME Element 3.3 requires that a medical school
“has effective policies and practices in place, and engages in ongoing, systematic, and focused recruitment and retention activities, to achieve mission-appropriate diversity outcomes among its students, faculty, senior administrative staff, and other relevant members of its academic community.”
Functions and Structure of a Medical School, Standards for Accreditation of Medical Education Programs Leading to the MD Degree (March 2023) (the “Accreditation Standards”), available at: https://lcme.org/publications/
21

## Slide 22
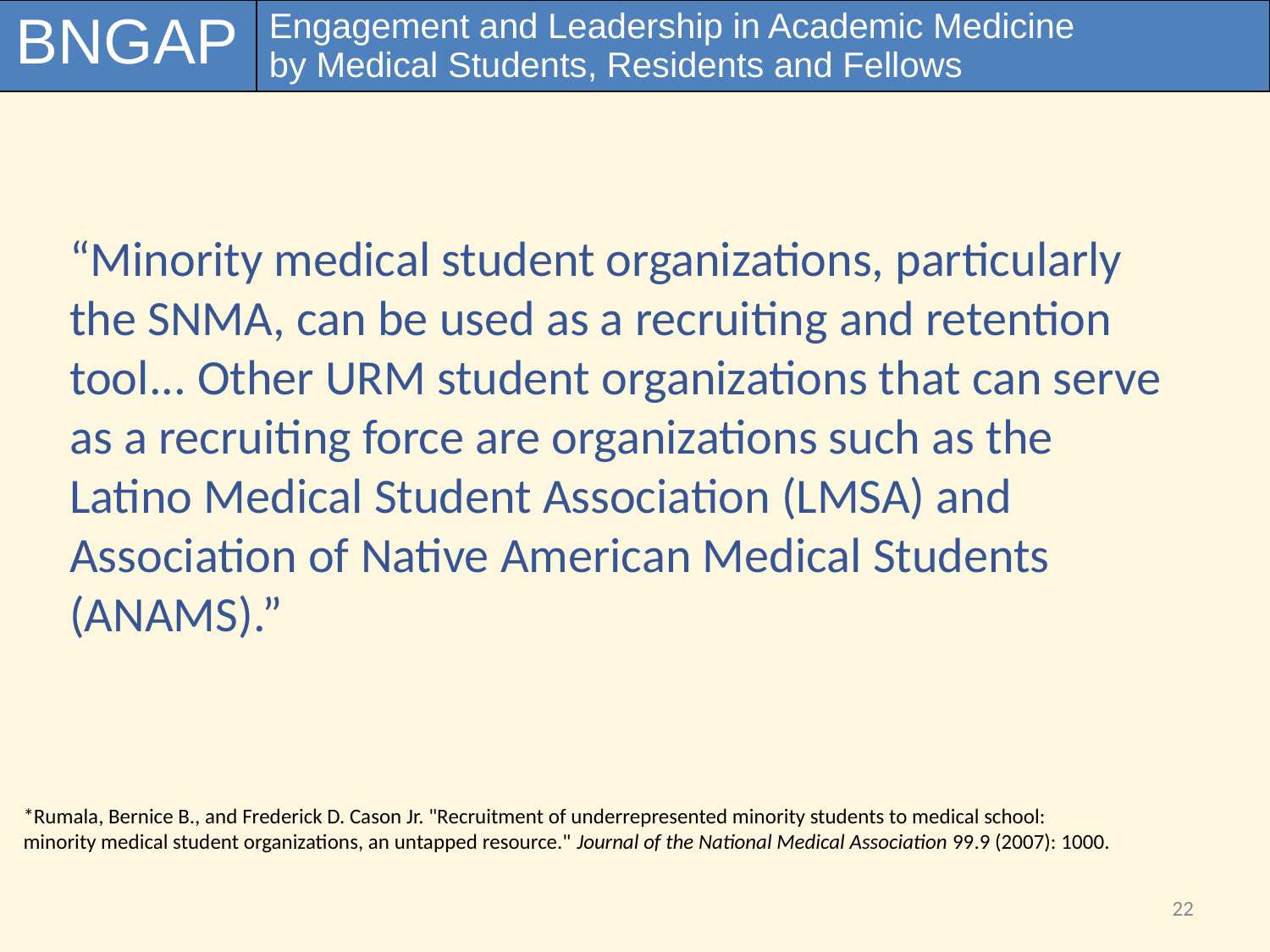

“Minority medical student organizations, particularly the SNMA, can be used as a recruiting and retention tool... Other URM student organizations that can serve as a recruiting force are organizations such as the Latino Medical Student Association (LMSA) and Association of Native American Medical Students (ANAMS).”
*Rumala, Bernice B., and Frederick D. Cason Jr. "Recruitment of underrepresented minority students to medical school: minority medical student organizations, an untapped resource." Journal of the National Medical Association 99.9 (2007): 1000.
22

## Slide 23
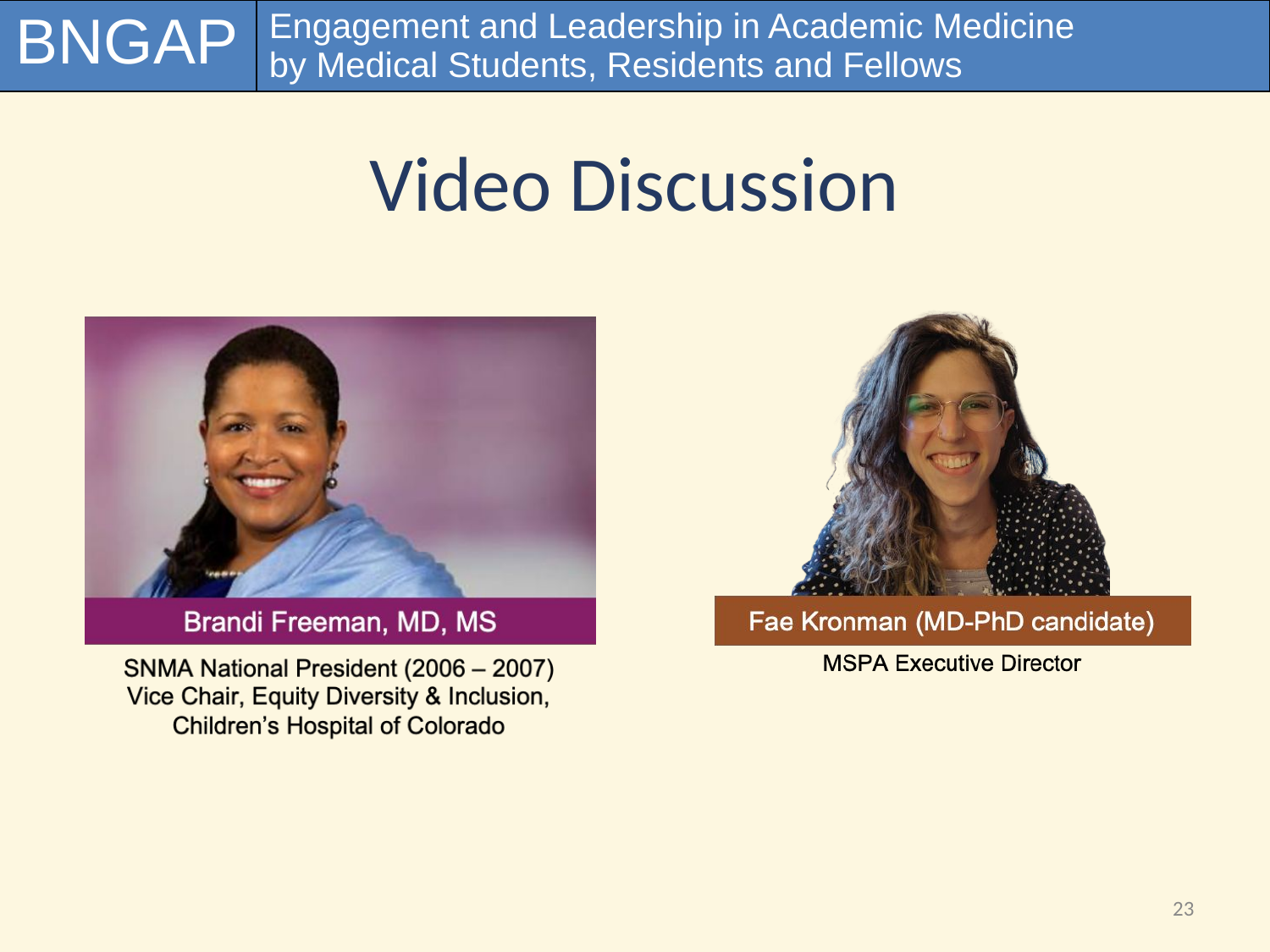

# Video Discussion
23

## Slide 24
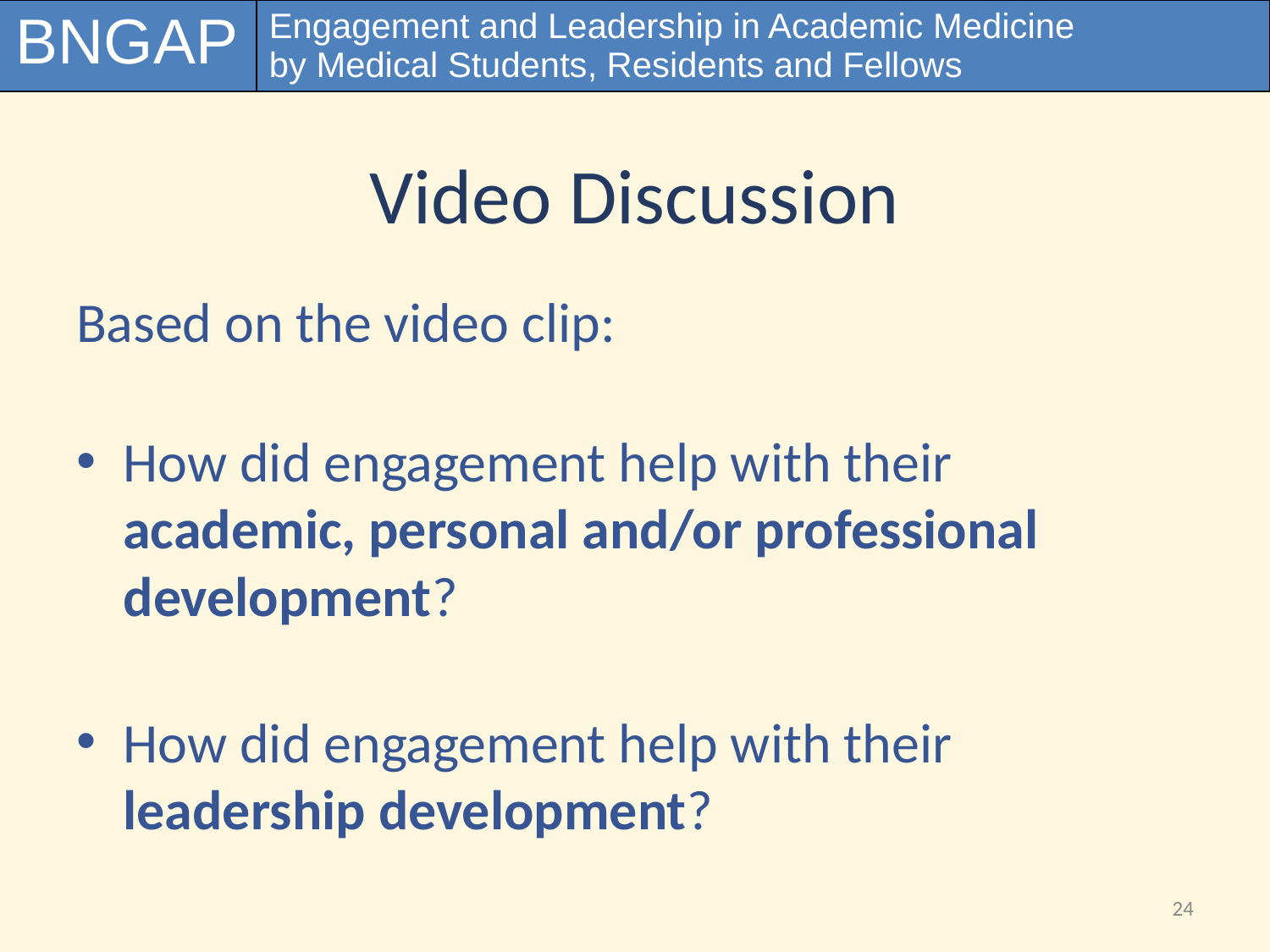

# Video Discussion
Based on the video clip:
How did engagement help with their academic, personal and/or professional development?
How did engagement help with their leadership development?
24

## Slide 25
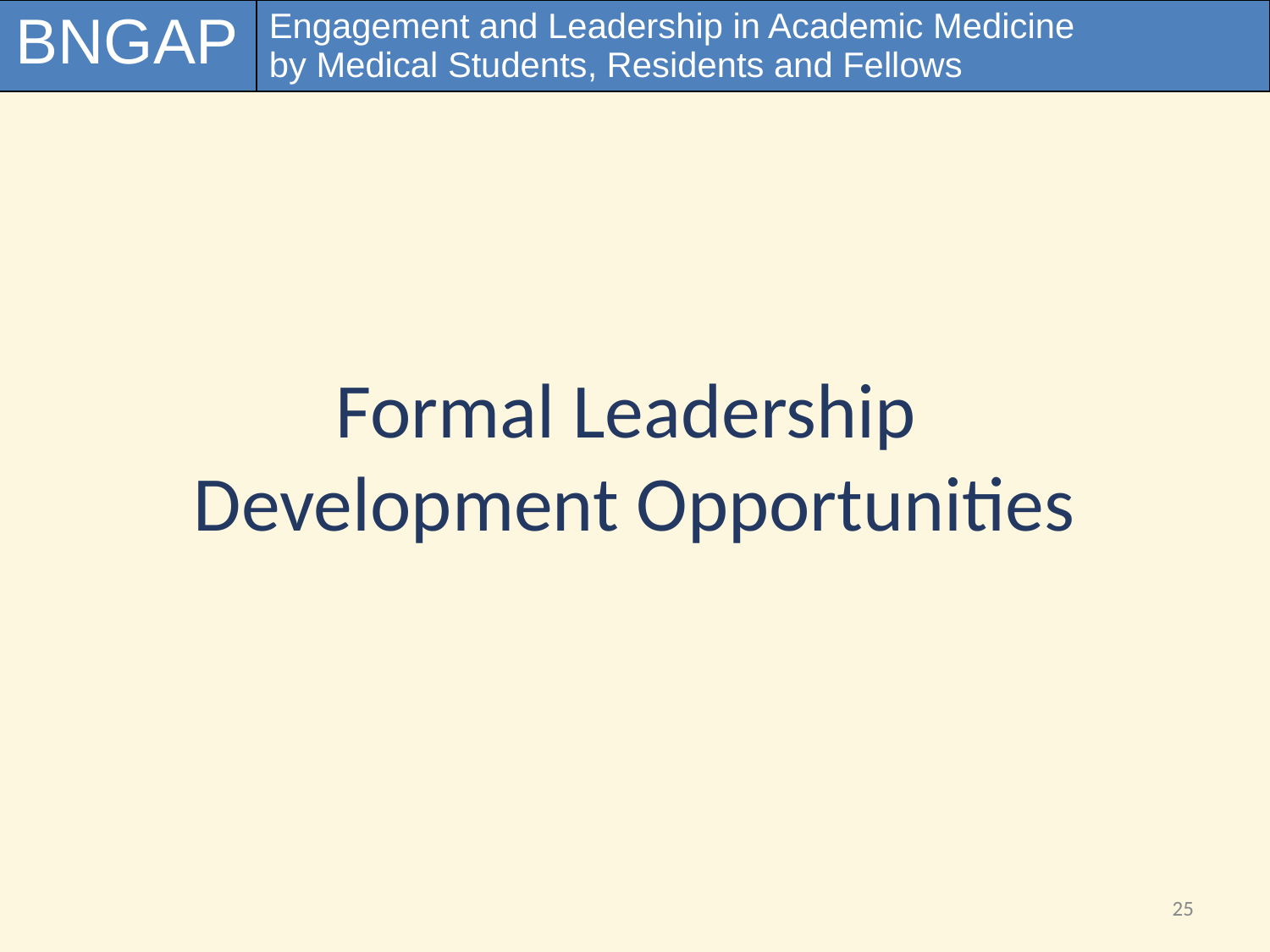

# Formal Leadership
Development Opportunities
25

## Slide 26
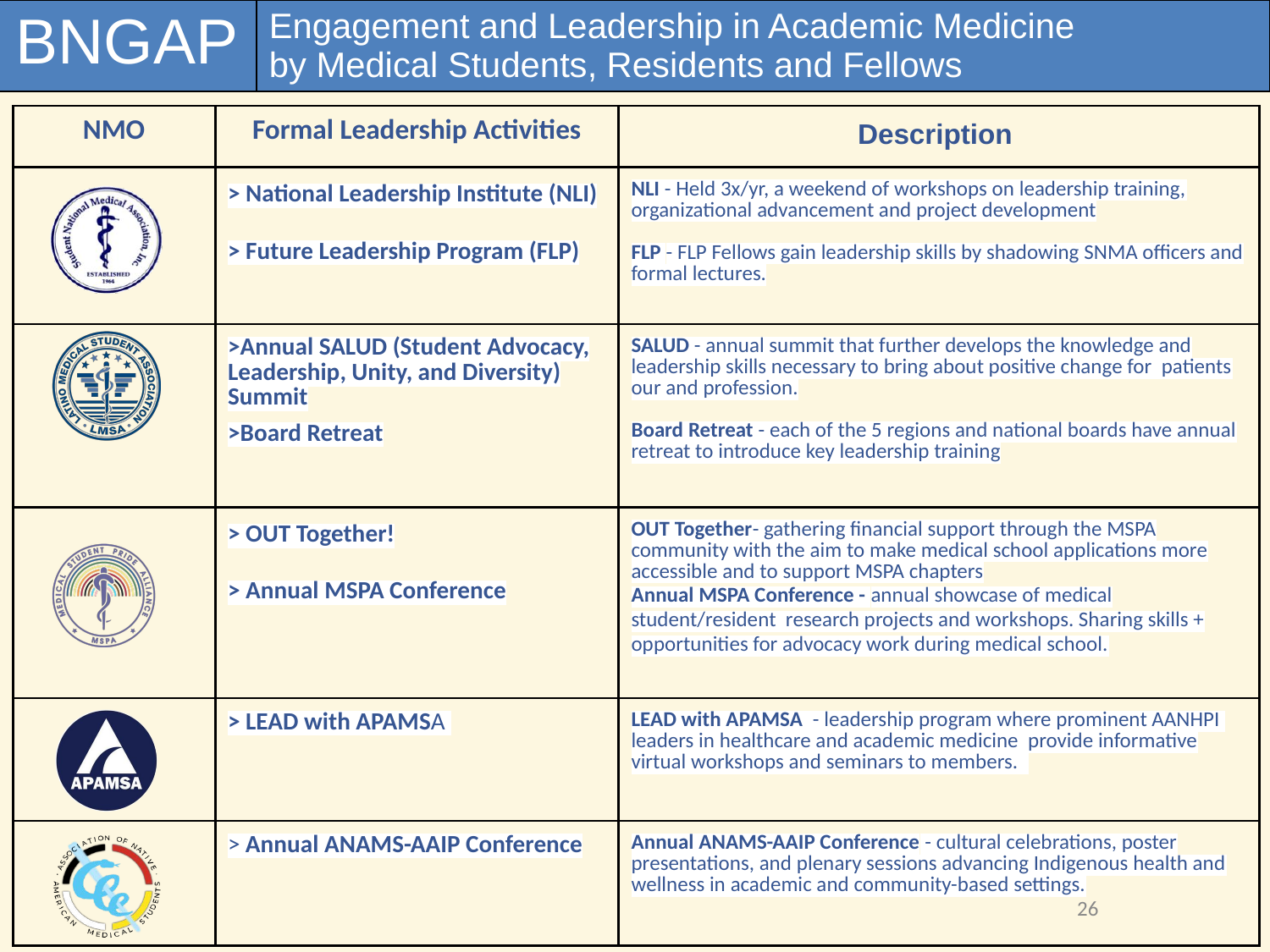

| NMO | Formal Leadership Activities | Description |
| --- | --- | --- |
| | > National Leadership Institute (NLI) > Future Leadership Program (FLP) | NLI - Held 3x/yr, a weekend of workshops on leadership training, organizational advancement and project development FLP - FLP Fellows gain leadership skills by shadowing SNMA officers and formal lectures. |
| | >Annual SALUD (Student Advocacy, Leadership, Unity, and Diversity) Summit >Board Retreat | SALUD - annual summit that further develops the knowledge and leadership skills necessary to bring about positive change for patients our and profession. Board Retreat - each of the 5 regions and national boards have annual retreat to introduce key leadership training |
| | > OUT Together! > Annual MSPA Conference | OUT Together- gathering financial support through the MSPA community with the aim to make medical school applications more accessible and to support MSPA chapters Annual MSPA Conference - annual showcase of medical student/resident research projects and workshops. Sharing skills + opportunities for advocacy work during medical school. |
| | > LEAD with APAMSA | LEAD with APAMSA - leadership program where prominent AANHPI leaders in healthcare and academic medicine provide informative virtual workshops and seminars to members. |
| | > Annual ANAMS-AAIP Conference | Annual ANAMS-AAIP Conference - cultural celebrations, poster presentations, and plenary sessions advancing Indigenous health and wellness in academic and community-based settings. |
26

## Slide 27
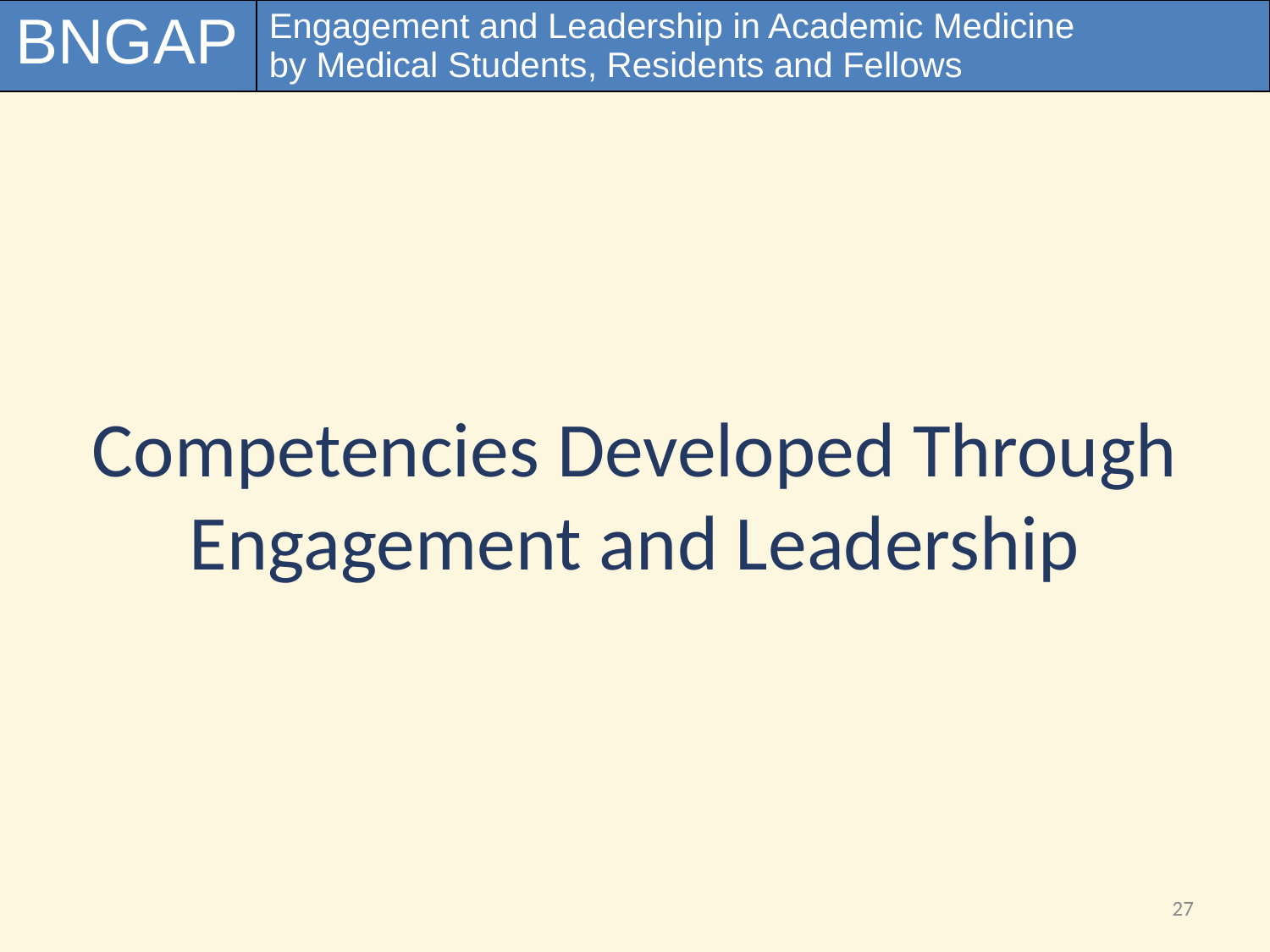

# Competencies Developed Through Engagement and Leadership
27

## Slide 28
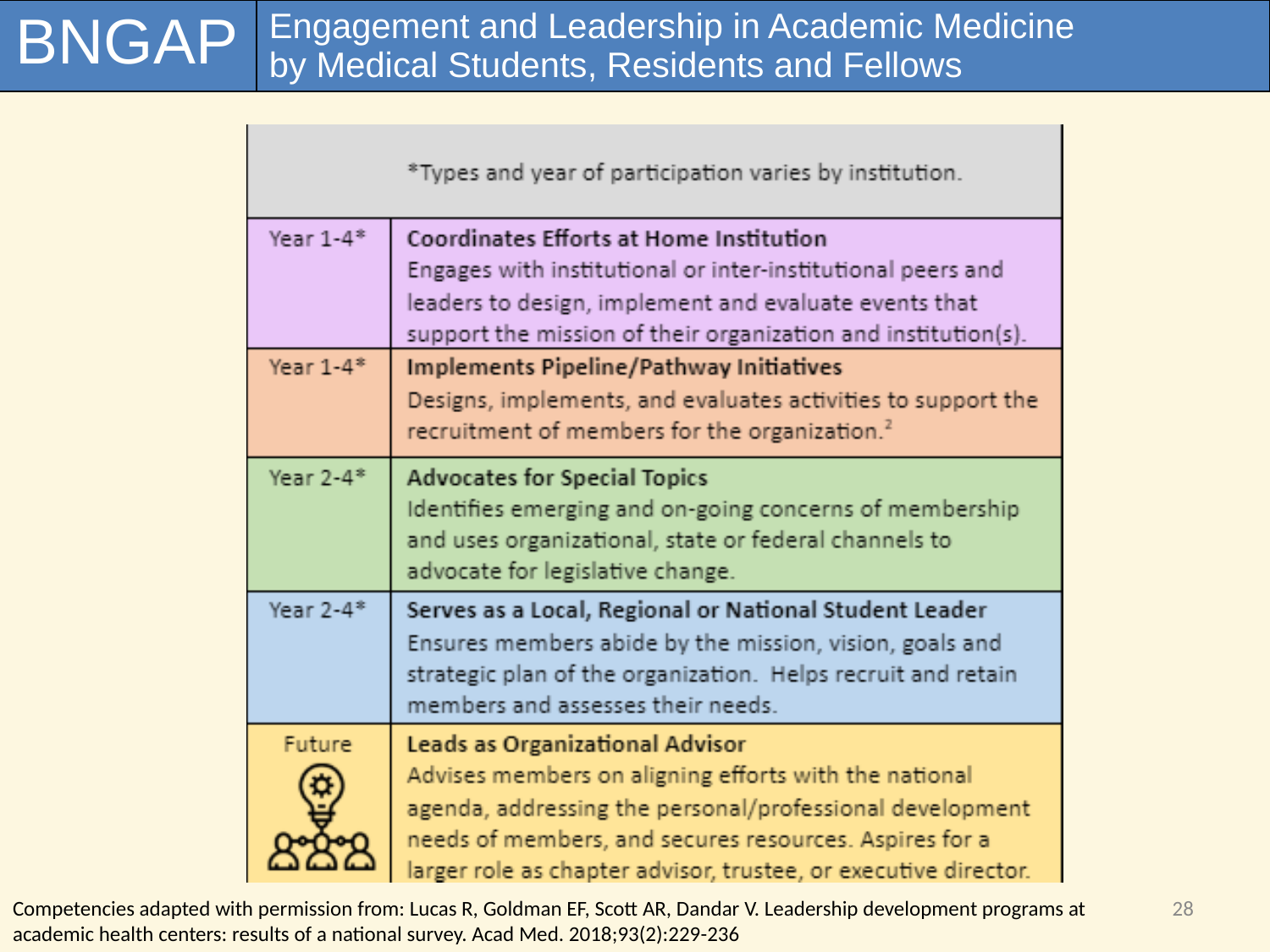

Competencies adapted with permission from: Lucas R, Goldman EF, Scott AR, Dandar V. Leadership development programs at academic health centers: results of a national survey. Acad Med. 2018;93(2):229-236
28

## Slide 29
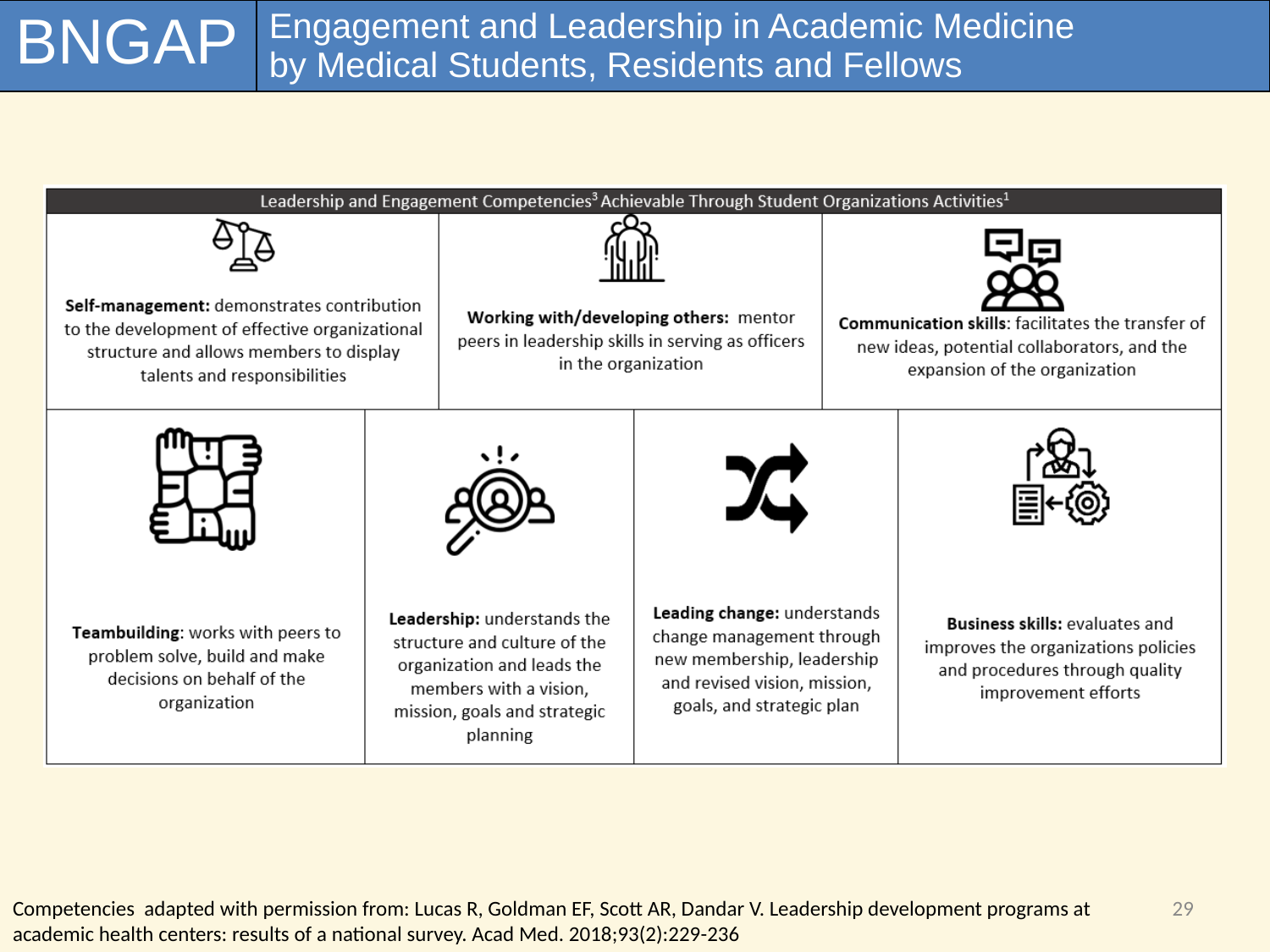

Competencies adapted with permission from: Lucas R, Goldman EF, Scott AR, Dandar V. Leadership development programs at academic health centers: results of a national survey. Acad Med. 2018;93(2):229-236
29

## Slide 30
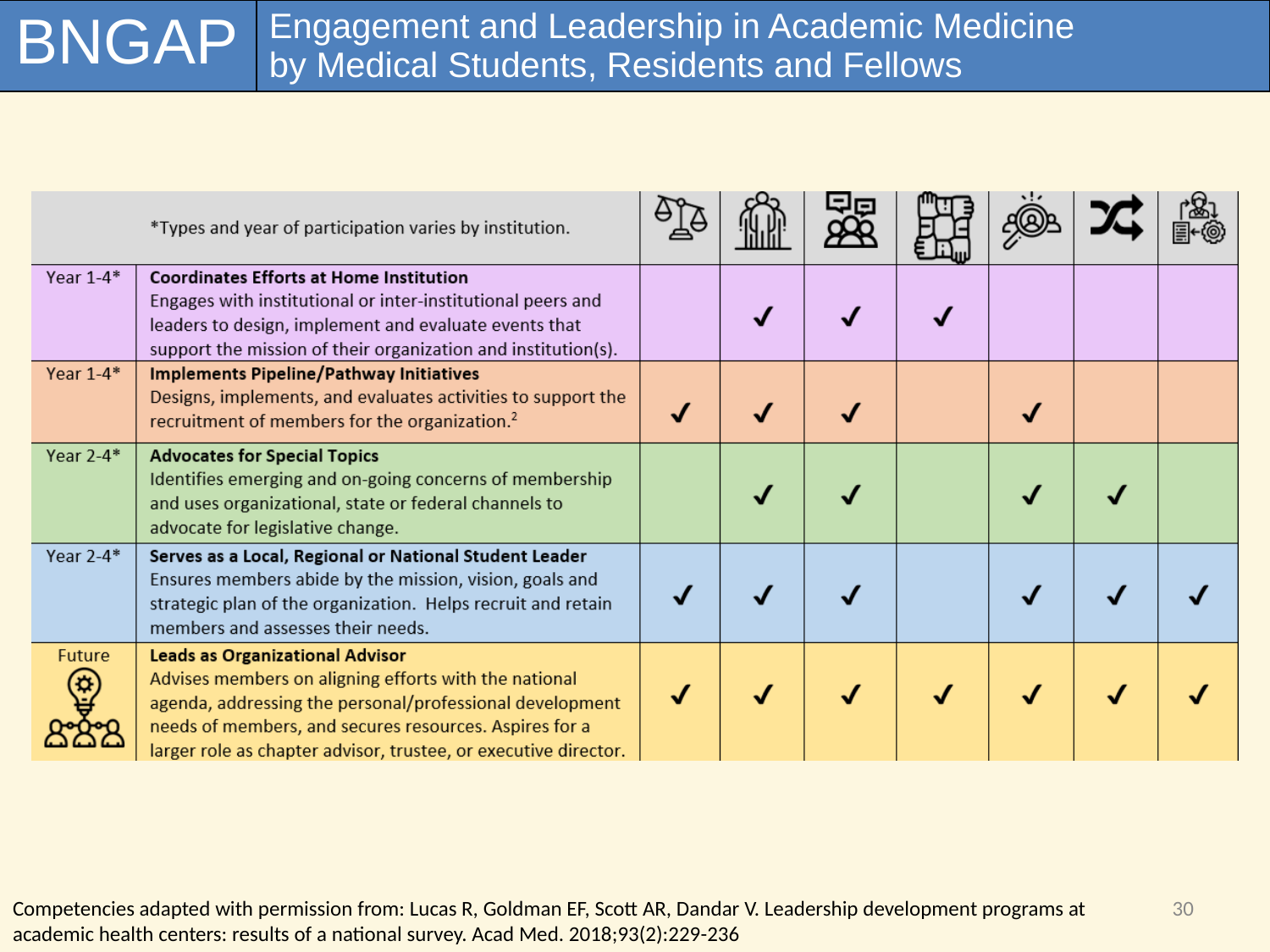

Competencies adapted with permission from: Lucas R, Goldman EF, Scott AR, Dandar V. Leadership development programs at academic health centers: results of a national survey. Acad Med. 2018;93(2):229-236
30

## Slide 31
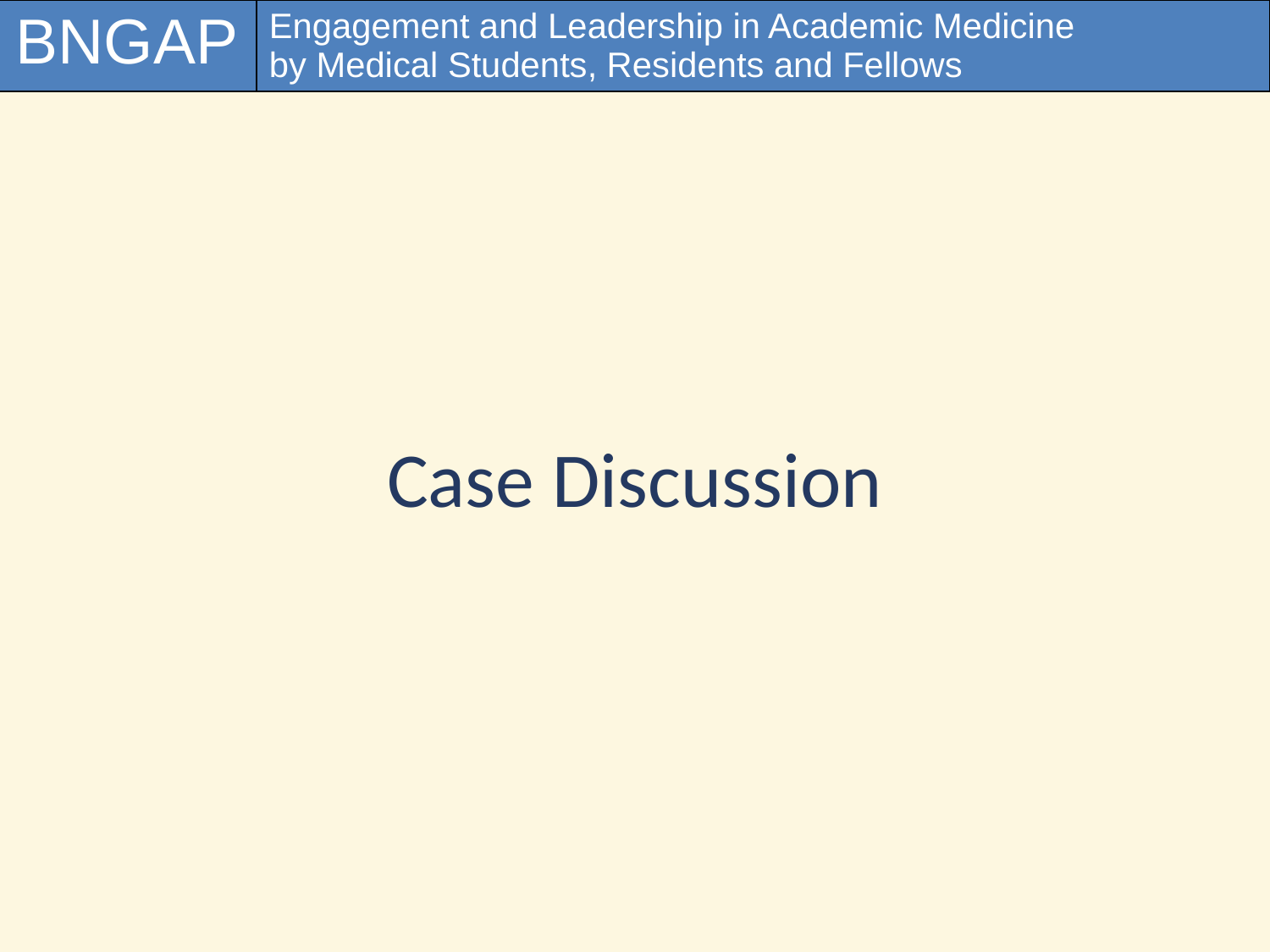

# Case Discussion

## Slide 32
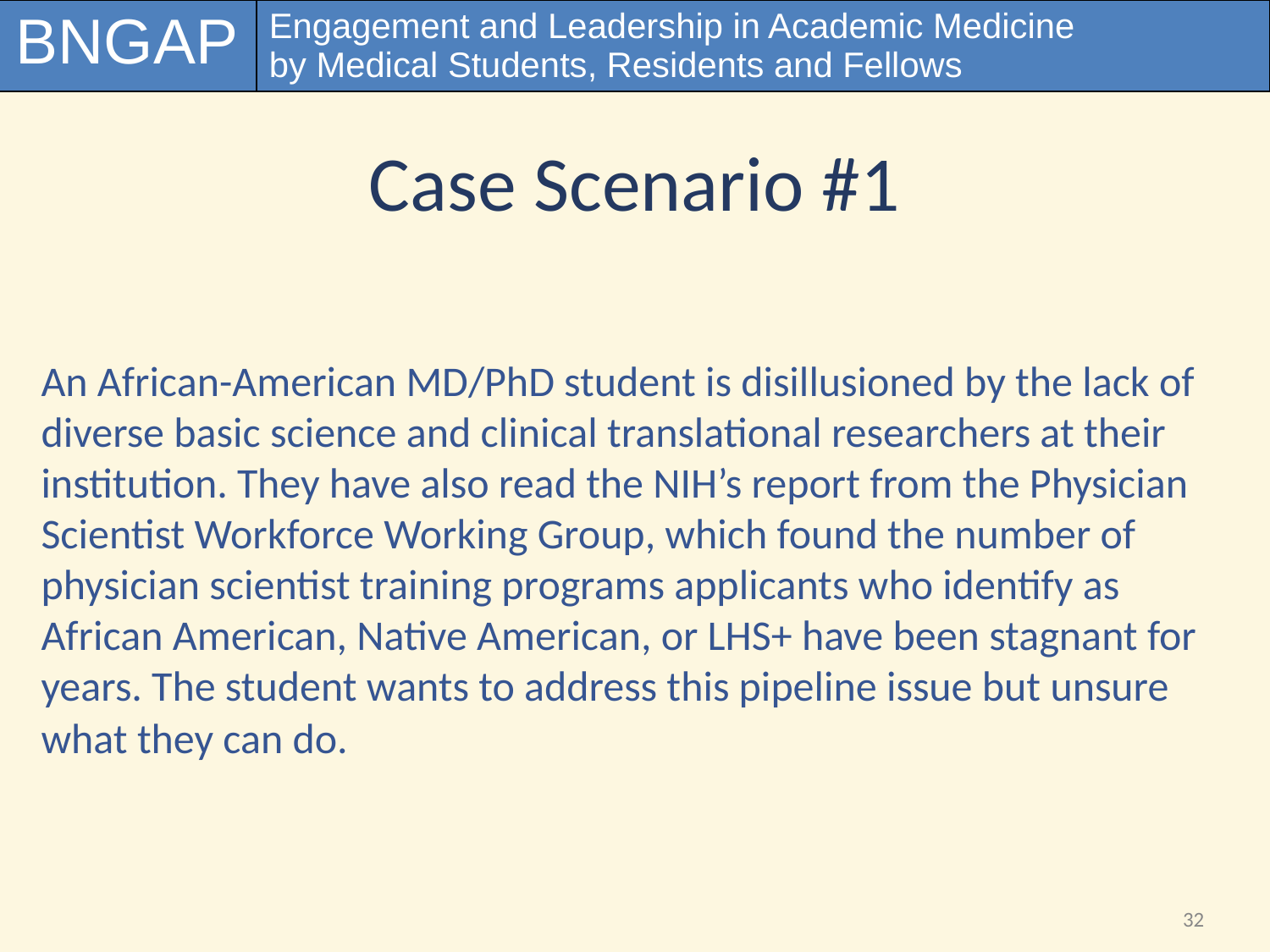

# Case Scenario #1
An African-American MD/PhD student is disillusioned by the lack of diverse basic science and clinical translational researchers at their institution. They have also read the NIH’s report from the Physician Scientist Workforce Working Group, which found the number of physician scientist training programs applicants who identify as African American, Native American, or LHS+ have been stagnant for years. The student wants to address this pipeline issue but unsure what they can do.
32

## Slide 33
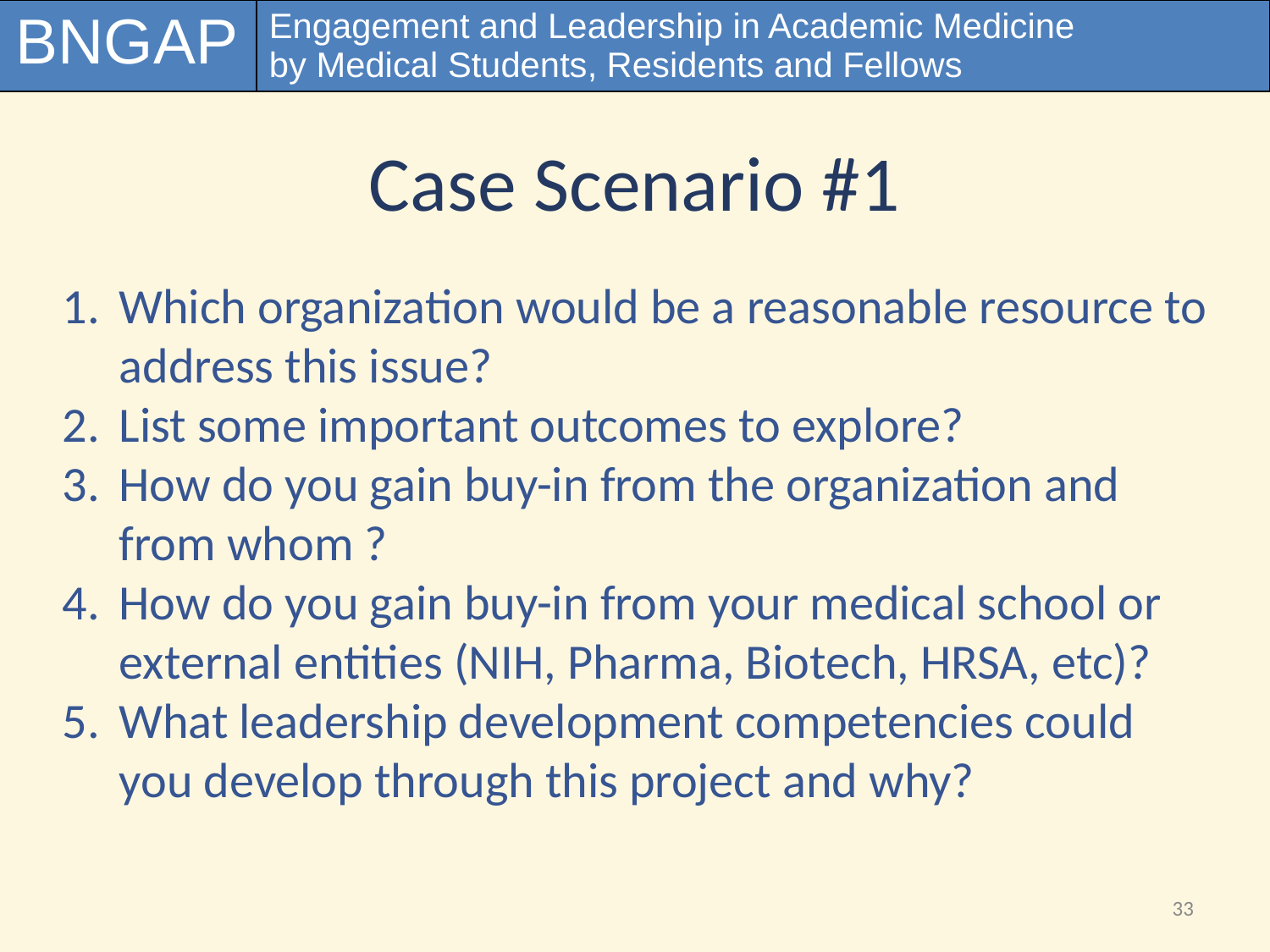

# Case Scenario #1
Which organization would be a reasonable resource to address this issue?
List some important outcomes to explore?
How do you gain buy-in from the organization and from whom ?
How do you gain buy-in from your medical school or external entities (NIH, Pharma, Biotech, HRSA, etc)?
What leadership development competencies could you develop through this project and why?
33

## Slide 34
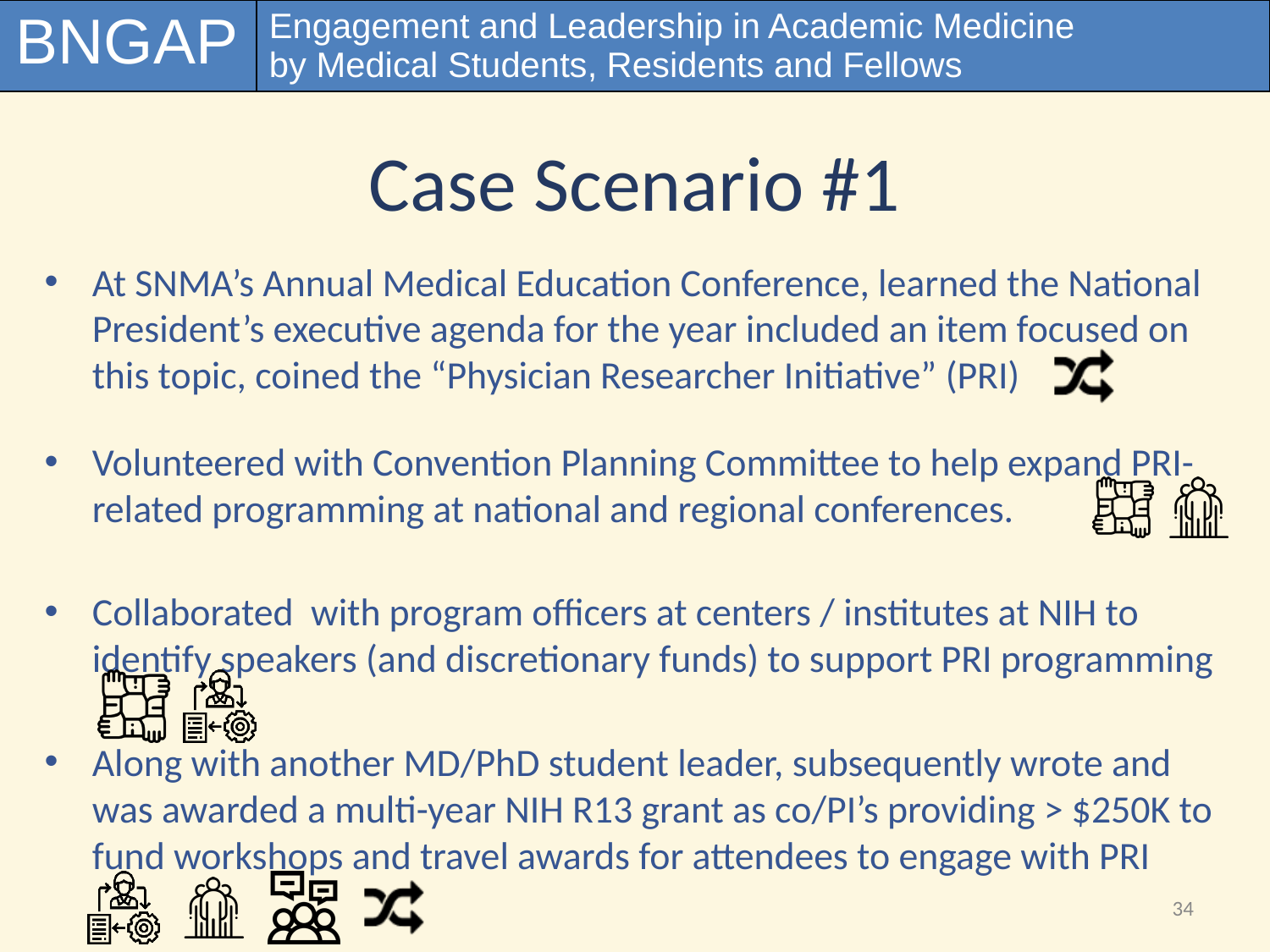

At SNMA’s Annual Medical Education Conference, learned the National President’s executive agenda for the year included an item focused on this topic, coined the “Physician Researcher Initiative” (PRI)
Volunteered with Convention Planning Committee to help expand PRI-related programming at national and regional conferences.
Collaborated with program officers at centers / institutes at NIH to identify speakers (and discretionary funds) to support PRI programming
Along with another MD/PhD student leader, subsequently wrote and was awarded a multi-year NIH R13 grant as co/PI’s providing > $250K to fund workshops and travel awards for attendees to engage with PRI
# Case Scenario #1
34

## Slide 35
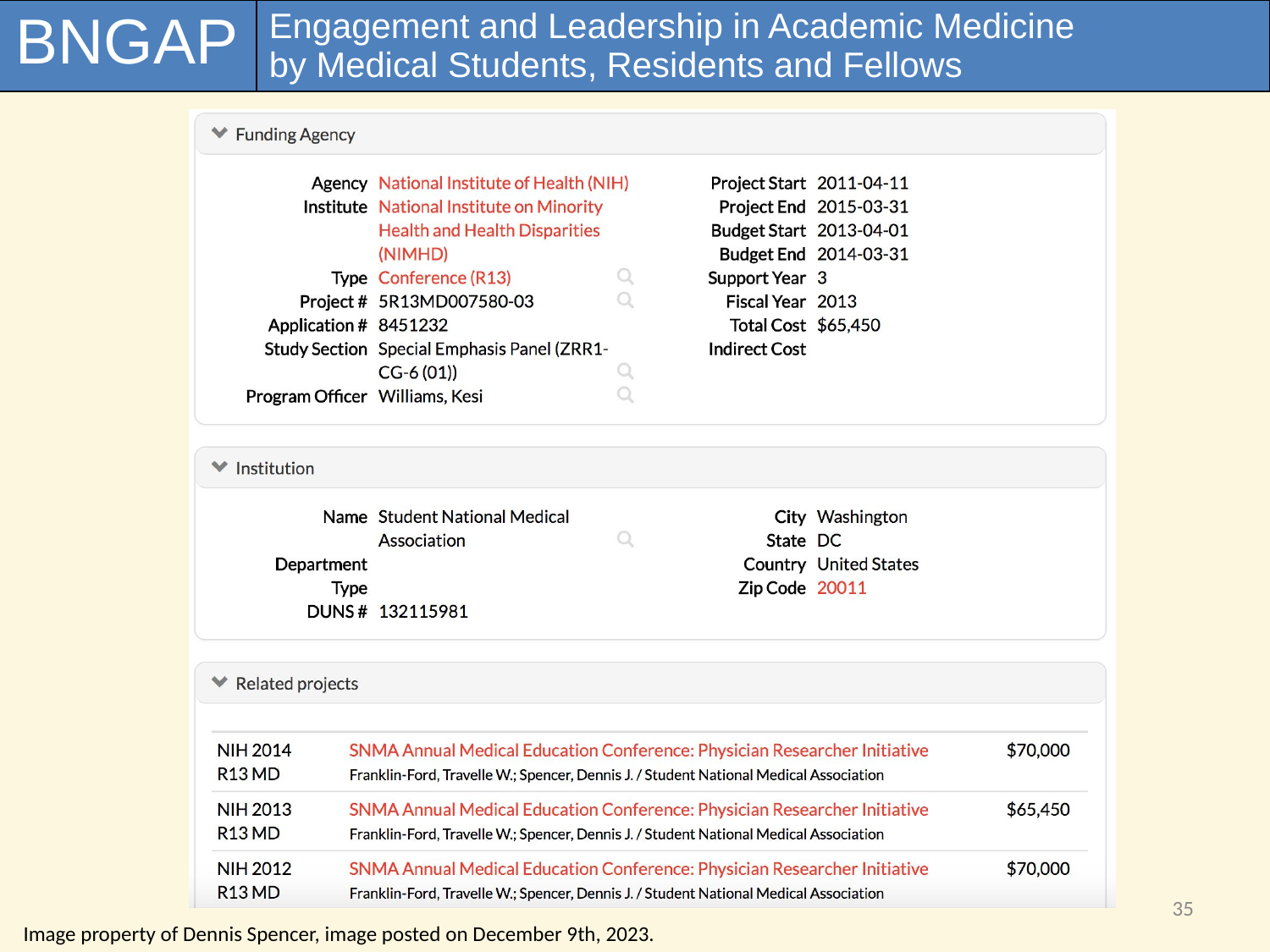

35
Image property of Dennis Spencer, image posted on December 9th, 2023.

## Slide 36
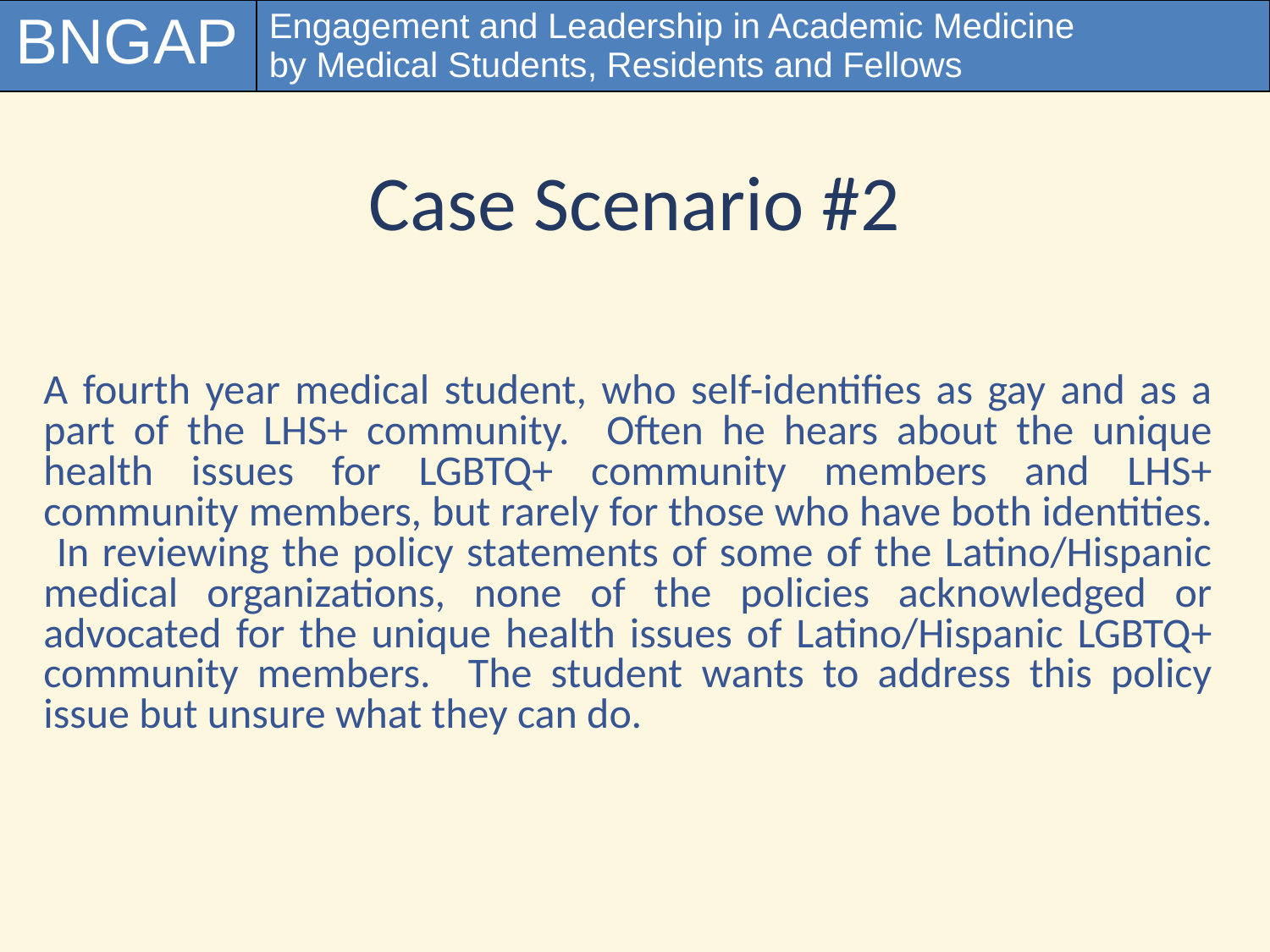

# Case Scenario #2
A fourth year medical student, who self-identifies as gay and as a part of the LHS+ community. Often he hears about the unique health issues for LGBTQ+ community members and LHS+ community members, but rarely for those who have both identities. In reviewing the policy statements of some of the Latino/Hispanic medical organizations, none of the policies acknowledged or advocated for the unique health issues of Latino/Hispanic LGBTQ+ community members. The student wants to address this policy issue but unsure what they can do.

## Slide 37
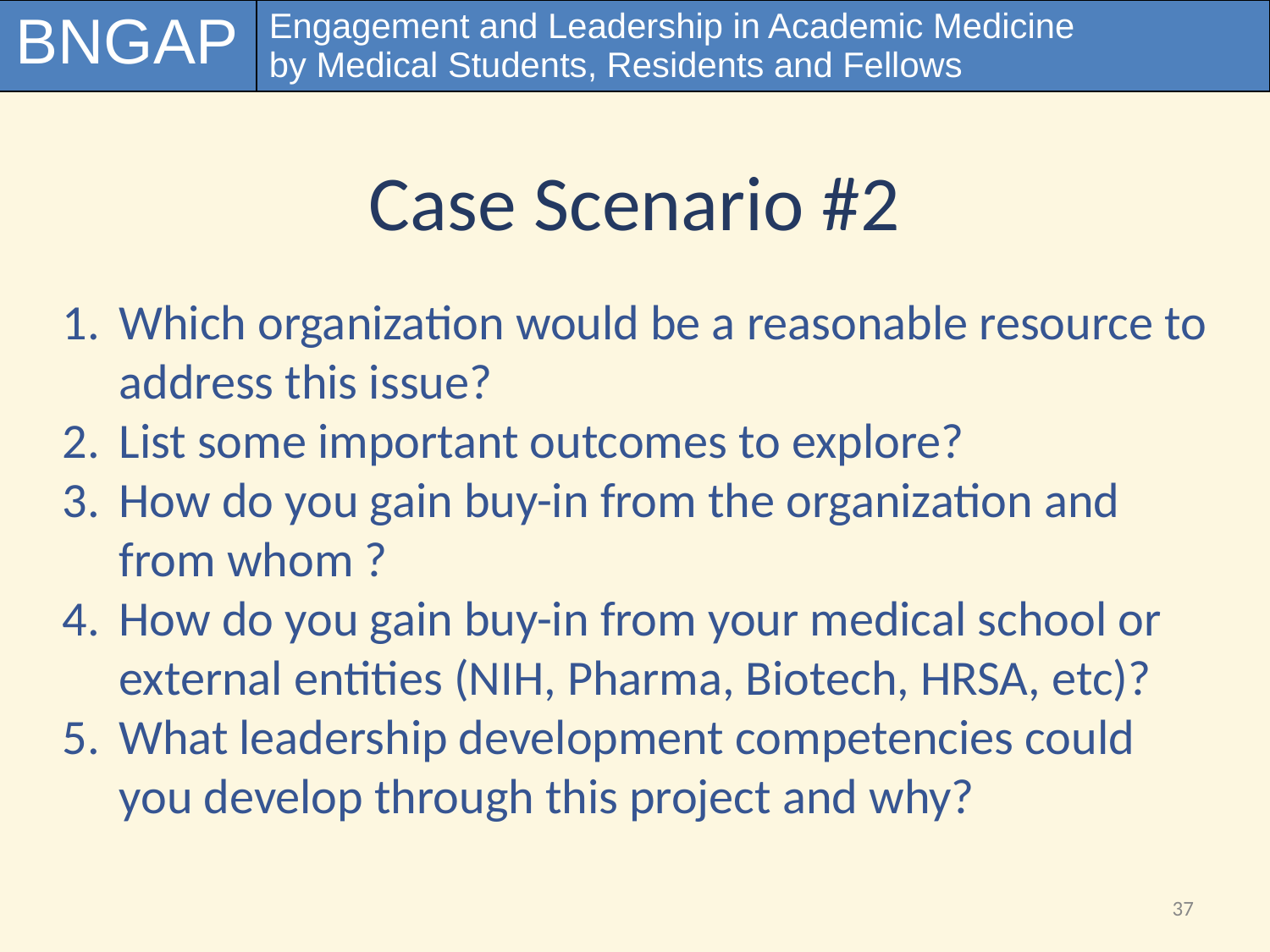

# Case Scenario #2
Which organization would be a reasonable resource to address this issue?
List some important outcomes to explore?
How do you gain buy-in from the organization and from whom ?
How do you gain buy-in from your medical school or external entities (NIH, Pharma, Biotech, HRSA, etc)?
What leadership development competencies could you develop through this project and why?
37

## Slide 38
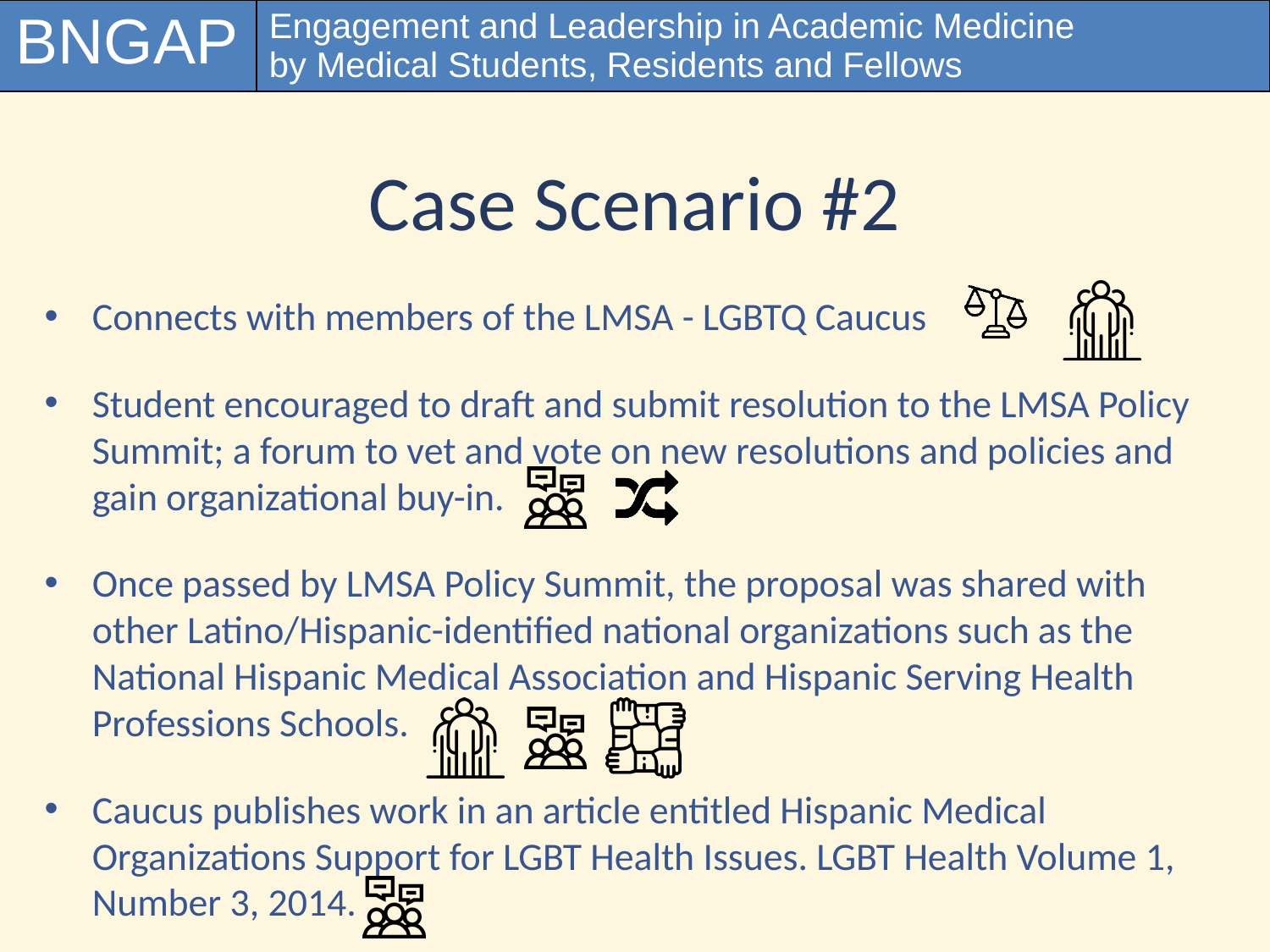

# Case Scenario #2
Connects with members of the LMSA - LGBTQ Caucus
Student encouraged to draft and submit resolution to the LMSA Policy Summit; a forum to vet and vote on new resolutions and policies and gain organizational buy-in.
Once passed by LMSA Policy Summit, the proposal was shared with other Latino/Hispanic-identified national organizations such as the National Hispanic Medical Association and Hispanic Serving Health Professions Schools.
Caucus publishes work in an article entitled Hispanic Medical Organizations Support for LGBT Health Issues. LGBT Health Volume 1, Number 3, 2014.

## Slide 39
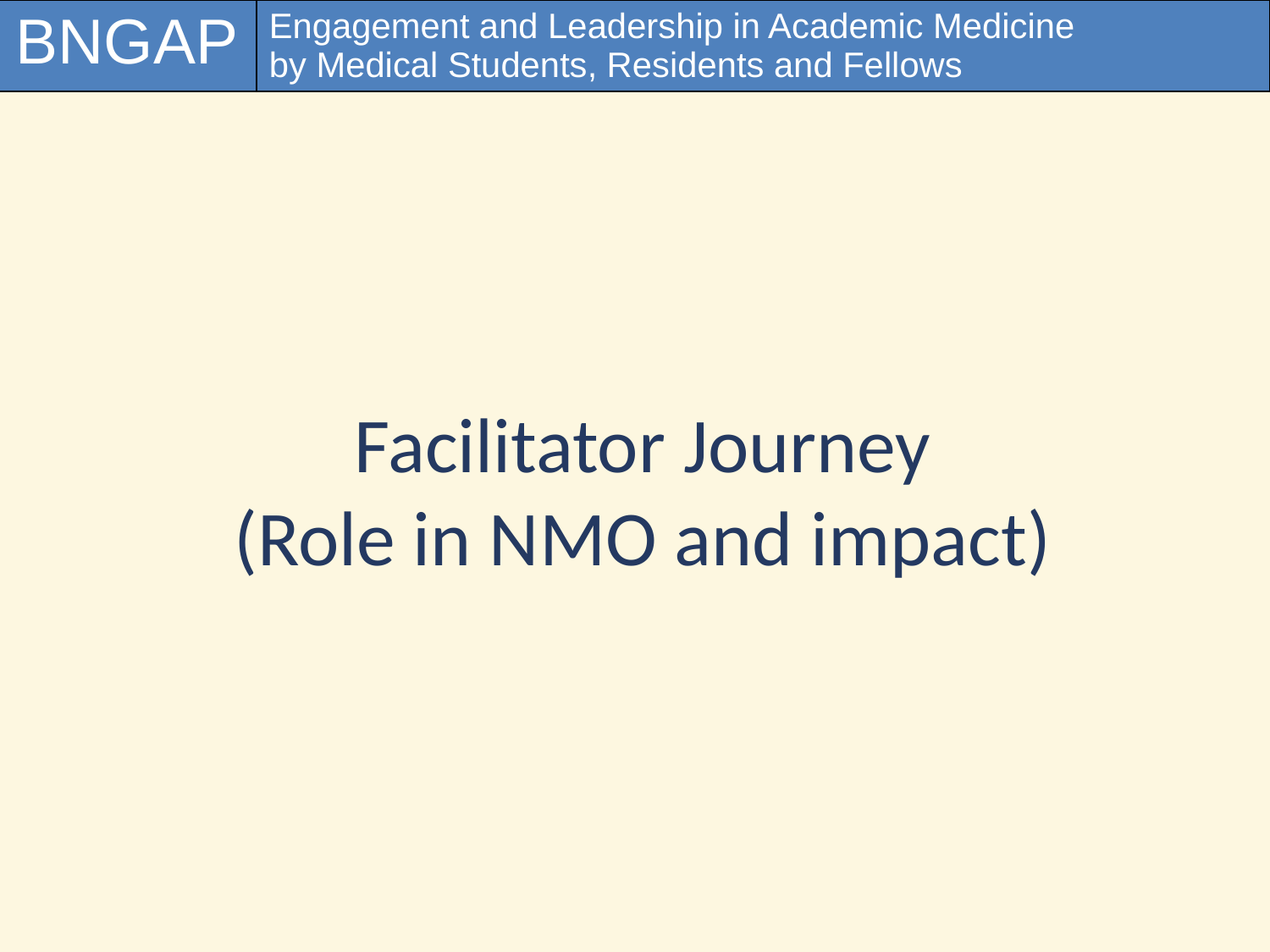

# Facilitator Journey (Role in NMO and impact)

## Slide 40
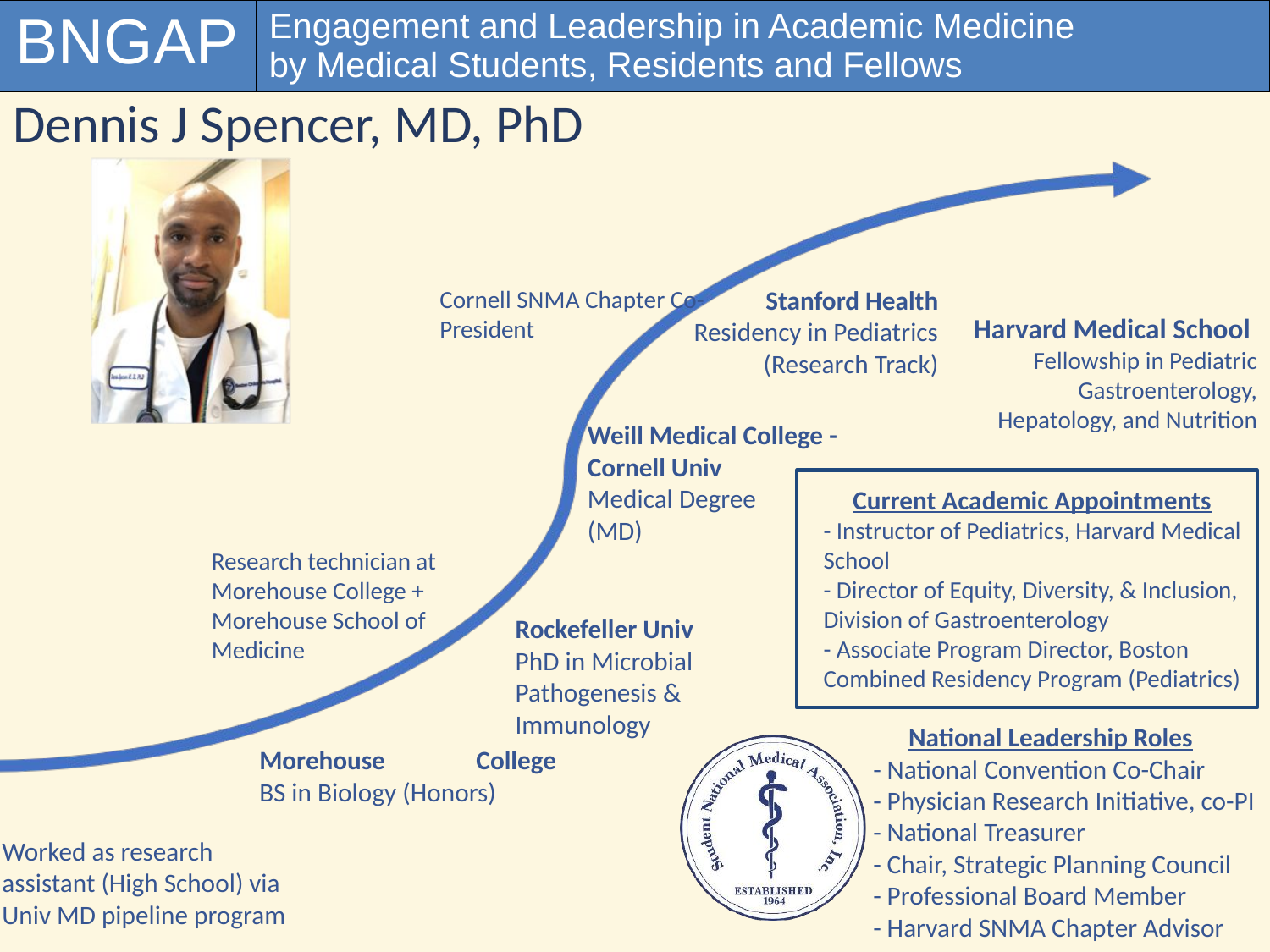

Dennis J Spencer, MD, PhD
Cornell SNMA Chapter Co-President
Stanford Health
Residency in Pediatrics (Research Track)
Harvard Medical School
Fellowship in Pediatric Gastroenterology,
 Hepatology, and Nutrition
Weill Medical College - Cornell Univ
Medical Degree
(MD)
 Current Academic Appointments
- Instructor of Pediatrics, Harvard Medical School
- Director of Equity, Diversity, & Inclusion, Division of Gastroenterology
- Associate Program Director, Boston Combined Residency Program (Pediatrics)
Research technician at Morehouse College + Morehouse School of Medicine
Rockefeller Univ
PhD in Microbial Pathogenesis & Immunology
 National Leadership Roles
- National Convention Co-Chair
- Physician Research Initiative, co-PI
- National Treasurer
- Chair, Strategic Planning Council
- Professional Board Member
- Harvard SNMA Chapter Advisor
Morehouse College
BS in Biology (Honors)
Worked as research assistant (High School) via Univ MD pipeline program

## Slide 41
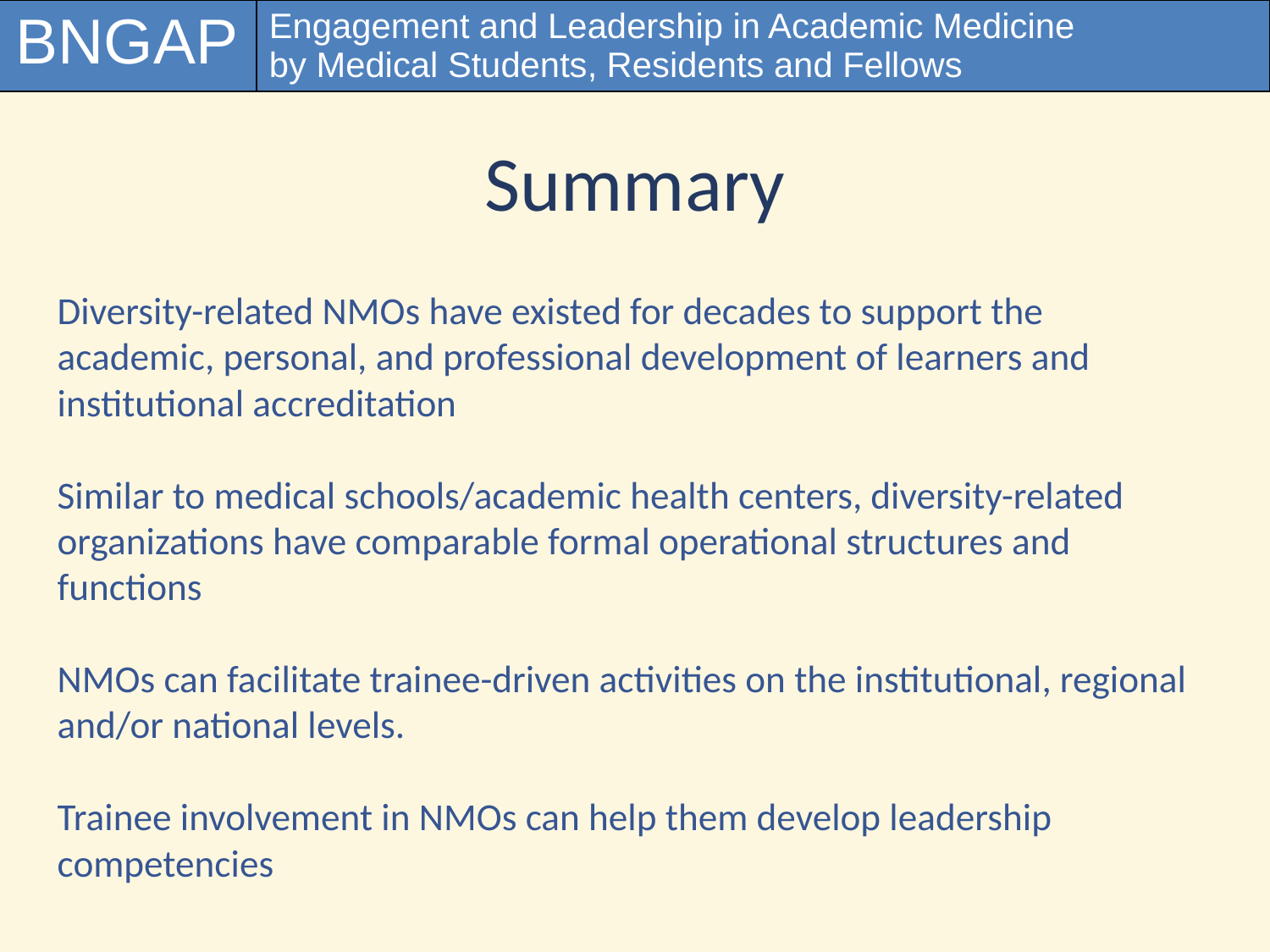

# Summary
Diversity-related NMOs have existed for decades to support the academic, personal, and professional development of learners and institutional accreditation
Similar to medical schools/academic health centers, diversity-related organizations have comparable formal operational structures and functions
NMOs can facilitate trainee-driven activities on the institutional, regional and/or national levels.
Trainee involvement in NMOs can help them develop leadership competencies

## Slide 42
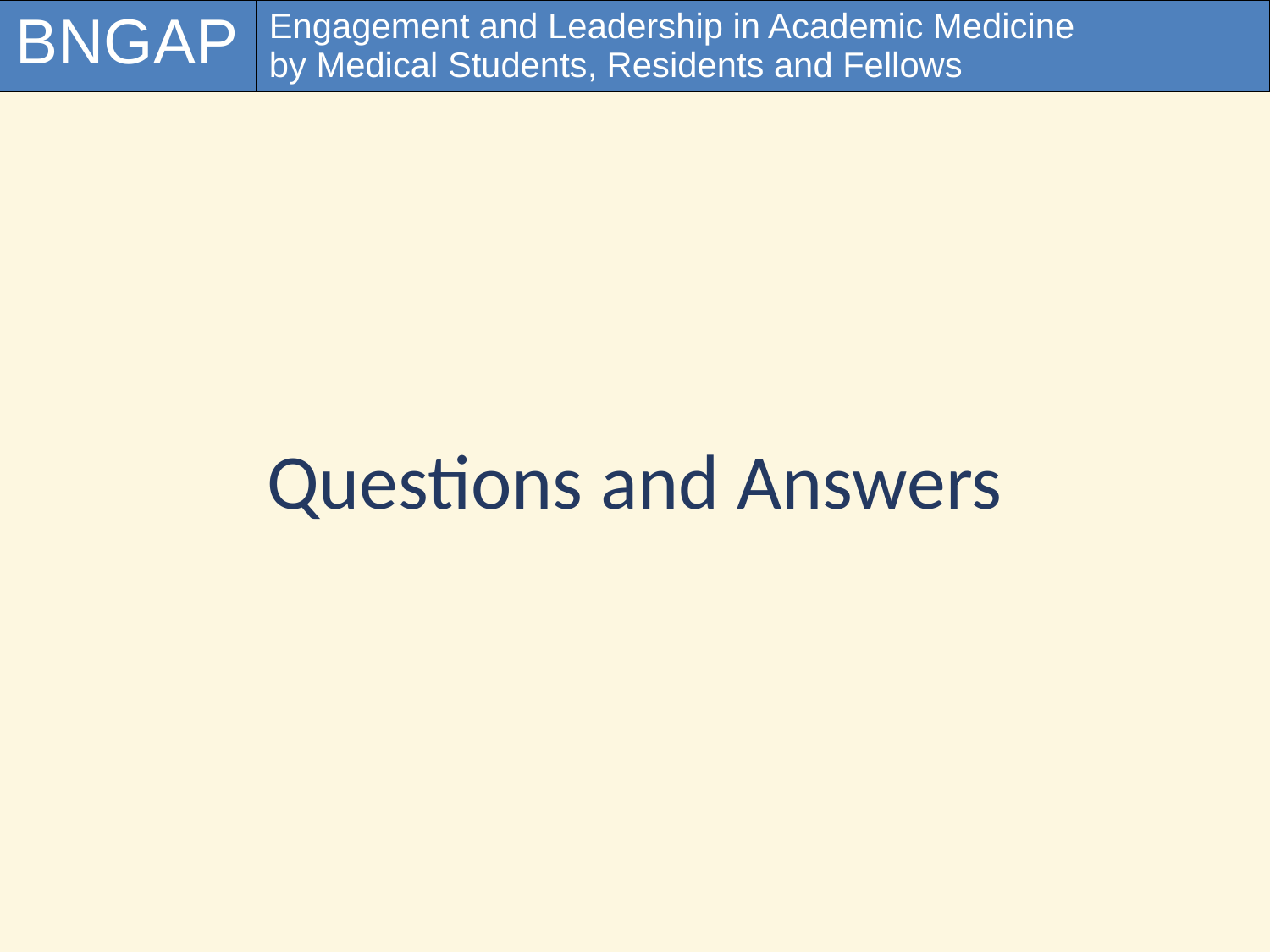

Questions and Answers
