# Supplementary material for: Diversity-Related, Student-Led National Medical Organizations: Leadership Opportunities for Learners
Source: MedEdPORTAL. 2024 Dec 27;20:11477. doi: 10.15766/mep_2374-8265.11477 (PMC11671812; doi:10.15766/mep_2374-8265.11477)
Supplement: Supplementary file 1 — Facilitator Guide.docxPre- and Postworkshop Survey.docxNMOs Presentation.pptxExample SNMA Strategic Plan.docxNMOs Activities Handout.docxDr. Freeman SNMA Testimonial.mp4Fae MSPA Testimonial.mov [file mep_2374-8265.11477-s001.zip › D. Example SNMA Strategic Plan.docx]

*Please disseminate this at the beginning of the session. This strategic plan serves as an example for students.*

*This excerpt of the Student National Medical Association’s 2011 – 2014 Strategic Planning document is meant for archival purposes only. A new strategic plan was created and adopted by the SNMA’s House of Delegates in 2017.*

*Permission for use of this archival document given by Dr. Dennis Spencer, MD, PhD, co-author of module and SNMA Professional Board Member / Past Chair of Strategic Planning Council.*


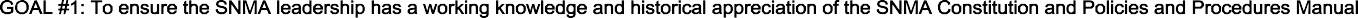


|  |  | |  |  |  |  |  |
| --- | --- | --- | --- | --- | --- | --- | --- |
|  | *Primary* | *Collaboration with* |  |  |  |  |  |
| Prepare RD and national officer candidates to function successfully within the SNMA's corporate structure | RD to EC President-elect | Internal Affairs Comm  Elections Comm | 1. Provide all national officer and RD candidates with a briefing session on planning documents, BOD structure, and corporate functionality prior to regional elections | #1 complete annually at AMEC prior to regional and national elections | Metric: 100% of RD and national candidates will participate in briefing session |  | Discussion on whether session will be mandated |

|  |  | |  |  |  |  |  |
| --- | --- | --- | --- | --- | --- | --- | --- |
|  | *Primary* | *Collaboration with* |  |  |  |  |  |
| Improve BOD members' working knowledge and historical appreciation of the SNMA's governing and planning documents | Internal Affairs Comm | Parliamentarian Speaker of the House  SPC | 1. Use training modules to educate BOD officers on governing and planning documents 2. SPC will conduct Organizational Assessment Survey for the Improvement of SNMA (OASIS) | #1 annually starting  June 2011  #2 annually starting  Dec 2010 | Metric: 100% of officers will have completed modules  Metric: 100% of BOD officers will report use of governing and planning documents during their term during survey  Metric: 100% of BOD officers will answer at least 85% of questions on governing and planning documents in survey | OASIS administered Dec 2010 to BOD and staff | Revise efforts as appropriate |

|  |  | |  |  |  |  |  |
| --- | --- | --- | --- | --- | --- | --- | --- |
|  | *Primary* | *Collaboration with* |  |  |  |  |  |
| Ensure that governing documents and current organizational practices accurately reflect each other | Parliamentarian | Internal Affairs Speaker of the House  RD to EC | 1. Review Policies and Procedures Manual and adjust appropriately 2. Review National Constitution and adjust appropriately 3. Review regional bylaws and adjust appropriately 4. RD to EC will submit most current regional constitutions to National Headquarters | #1 complete annually  starting January 2012, review odd numbered sections in odd years and even numbered sections in even years (i.e. 2011-2012 = even number)  #2 complete at least every 3 years starting 2012  #3 complete annually  starting June 2012, review odd numbered sections in odd years and even numbered sections in even years (i.e. 2011-2012 = even number)  #4 complete annually by June meeting | Deliverable: Report findings of review  Action: Recommend solutions as appropriate  Metric: Organizational practices and governing documents will reflect each other with 100% accuracy in the year of review  Action: Most current national and regional governing documents will be housed at National Headquarters |  |  |


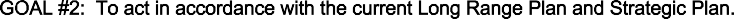


|  |  | |  |  |  |  |  |
| --- | --- | --- | --- | --- | --- | --- | --- |
|  | *Primary* | *Collaboration with* |  |  |  |  |  |

| Advance objectives of the Strategic Plan | Executive Director President-elect CHAIR-ELECT  Treasurer | SPC | 1. Incorporate strategies of the strategic plan into the Annual Operating Plan (a. Executive Agenda,  b. Corporate Agenda, c. Budget) and National HQ Planning Document   1. SPC will provide formal feedback as it pertains to the LRP and SP 2. Report progress towards the goals and objectives of LRP and SP 3. Engage BOD and SPC in interactions with respect to the organization's progress towards defined goals and objectives | EA feedback annually by Jan 15  CA feedback annually by Mar 1 or June 1  Budget feedback annually by May 15  HQ feedback by Sept 1  #3 quarterly BOD  starting June 2011  and annually at AMEC  #4 quarterly starting  Jan 2010 | Deliverable: EA, CA, and Budget, HQ plan will outline strategies directly from Strategic Plan  Metric: 100% of all outlined strategies will be achieved  Action: SPC will present a strategic planning session at each NLI  Deliverable: quarterly report from SPC outlining interactions  Action: review and revise SP as appropriate |  |  |
| --- | --- | --- | --- | --- | --- | --- | --- |


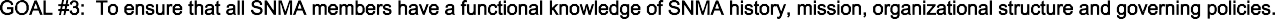


|  |  | |  |  |  |  |  |
| --- | --- | --- | --- | --- | --- | --- | --- |
|  | *Primary* | *Collaboration with* |  |  |  |  |  |
| Utilize SNMA chapters for the standardized and effective education of all SNMA members. | Internal Affairs Comm | RD to EC Membership Comm Designated staff | 1. Engage chapter presidents and treasurers in "train the trainer" leadership modules 2. Create chapter incentives for completion of modules 3. Incorporate questions assessing use of modules in the OASIS survey | #1 annually starting  AMEC 2012  #2 complete by Jan 2012  #3 complete annually  by Dec 2011 | Deliverable: Train the trainer curriculum  Action: Train the trainer sessions will take place  Action: Implement incentives  Metric: 100% of chapters will report using modules to engage members in leadership training | OASIS survey administered by SPC in Dec 2010 to BOD and staff only | Discuss if incentives should be mandates  Expand OASIS to chapter presidents |

|  |  | |  |  |  |  |  |
| --- | --- | --- | --- | --- | --- | --- | --- |
|  | *Primary* | *Collaboration with* |  |  |  |  |  |
| Increase SNMA members' working knowledge of national and regional governing documents | Internal Affairs Comm | RD to EC Parliamentarian SPC  Membership Comm | 1. Make membership training modules available online 2. Create member incentives for completion of modules 3. Incorporate questions assessing knowledge in OASIS survey | #1 pilot by Jan 2012; go live by Apr 2012  #2 complete by 2012  #3 complete by Dec 2011 | Deliverable: Membership training curriculum  Action: Implement incentives  Action: Membership training modules will be available online  Metric: 85% of surveyed members will answer 85% of questions about SNMA governance correctly | OASIS survey administered by SPC in Dec 2010 to BOD and staff only | Expand OASIS to members |


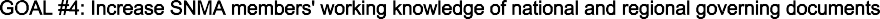


|  |  | |  |  |  |  |  |
| --- | --- | --- | --- | --- | --- | --- | --- |
|  | *Primary* | *Collaboration with* |  |  |  |  |  |

| Improve the preparedness of Chairperson of the BOD and National Treasurer for their offices | Internal Affairs Comm | SPC | 1. Create task force to research options and recommend a formal training period and plan for Chair and Treasurer 2. Implement plan 3. Create a training pre-test and post- test to administer to Chair and Treasurer | #1 complete by April 2011  #2 beginning 2011-  2012 administrative year  #3 pre-test within one week after election; post-test complete by Jan 1 annually | Deliverable: Recommendations for training period and plan  Action: Formal training period and program implemented  Metric: Increased score from pre-test to post-test | Task force convened and recommendations submitted for AMEC 2011 | Assess impact of training on Corporate Agenda and projected budget  Assess impact of training on corporate status |
| --- | --- | --- | --- | --- | --- | --- | --- |

|  |  | |  |  |  |  |  |
| --- | --- | --- | --- | --- | --- | --- | --- |
|  | *Primary* | *Collaboration with* |  |  |  |  |  |
| Engage all BOD officers and staff in annual objectives outlined in the Executive and Corporate Agendas | President Chair | Executive Director | 1. Make agendas readily available to all BOD and staff members 2. Include agendas in staff training 3. Report on progress of agendas 4. Designate Professional Board Members with specific duties | #1 annually no more than one week after approval  #2 annually by June 1  #3 quarterly at BOD mtgs  #4 annually by June mtg | Action: Agendas will be available online  Metric: 100% of staff will  be trained on each agenda  Action: Chair and President will report on progress at each mtg  Action: Each PBM will report progress on a specific assigned task | President currently reports progress on Exec Agenda |  |


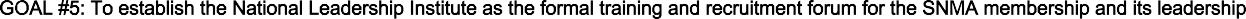


|  |  | |  |  |  |  |  |
| --- | --- | --- | --- | --- | --- | --- | --- |
|  | *Primary* | *Collaboration with* |  |  |  |  |  |
| Standardize the National | Internal Affairs | All BOD officers and | 1. Develop a series of modules that | #1 complete by May | Deliverable: Training | Modules approved in | Revise modules |
| Leadership Institute | Comm | committees | include basic organizational and | 2012 | modules hardcopy or | 2006-2007 | as needed |
| curriculum to include |  |  | administrative information |  | electronic |  |  |
| organizational and |  |  |  | #2 annually starting |  | OASIS survey | Assess impact of |
| corporate leadership |  |  | 2. Create a tentative NLI schedule | May 2012 | Deliverable: Tentative | administered in Dec | curriculum on |
| training |  |  | outlining when the modules will be |  | schedule of modules | 2010 | recruitment of |
|  |  |  | conducted | #3 quarterly starting |  |  | national officer |
|  |  |  |  | June 2012 | Action: Modules to be |  | Candidates |
|  |  |  | 3. Implement curriculum |  | administered to |  |  |
|  |  |  |  | #4 annually complete | participants (officers, |  | Assess |
|  |  |  | 4. Assess impact of modules on BOD | by Dec | members, staff) at |  | Leadership |
|  |  |  | and staff organizational and |  | quarterly NLI |  | training at the |
|  |  |  | administrative knowledge using |  |  |  | regional level |
|  |  |  | OASIS survey |  | Metric: 100% of NLI |  |  |
|  |  |  |  |  | participants will complete |  |  |
|  |  |  |  |  | appropriate modules |  |  |
|  |  |  |  |  | Metric: 100% of BOD |  |  |
|  |  |  |  |  | members and staff will |  |  |
|  |  |  |  |  | correctly answer at least |  |  |
|  |  |  |  |  | 85% of survey questions |  |  |
|  |  |  |  |  | on |  |  |
|  |  |  |  |  | organization/administration |  |  |
|  |  |  |  |  | during OASIS survey |  |  |

|  |  | |  |  |  |  |  |
| --- | --- | --- | --- | --- | --- | --- | --- |
|  | *Primary* | *Collaboration with* |  |  |  |  |  |

| Recruit informed and talented individuals to the Board of Directors and Strategic Planning Council | Elections Committee | Internal Affairs Comm  RD to EC | 1. Create a comprehensive recruitment plan outlining opportunities to target students and professionals to the BOD and SPC 2. Implement plan 3. Include question on how candidate was recruited on election application | #1 complete plan by Sept 2012  #2 starting Sept 2012 | Deliverable: Written recruitment plan  Action: Strategies in plan are implemented  Metric: At least 50% of national and regional officer candidates will have been recruited by the plan  Metric: At least 25% of elected/appointed officers will have been recruited by the plan |  |  |
| --- | --- | --- | --- | --- | --- | --- | --- |
