# Supplementary material for: Diversity-Related, Student-Led National Medical Organizations: Leadership Opportunities for Learners
Source: MedEdPORTAL. 2024 Dec 27;20:11477. doi: 10.15766/mep_2374-8265.11477 (PMC11671812; doi:10.15766/mep_2374-8265.11477)
Supplement: Supplementary file 1 — Facilitator Guide.docxPre- and Postworkshop Survey.docxNMOs Presentation.pptxExample SNMA Strategic Plan.docxNMOs Activities Handout.docxDr. Freeman SNMA Testimonial.mp4Fae MSPA Testimonial.mov [file mep_2374-8265.11477-s001.zip › E. NMOs Activities Handout.docx]

*Please distribute this info graphic at the beginning of the session. This page correlates to slides 28-30.*

| **Diversity-Related, Student-Led National Medical Student Organizations**  **Leadership Development for Learners** |
| --- |

According to the 2018 AAMC Year Two Questionnaire approximately 23% of medical students plan to participate in medical school administration during their career. Diversity-related, student-led national medical organizations serve as one avenue for medical students to become engaged and develop competencies to serve as future administrative leaders.

**Diversity-Related, Student-Led National Medical Student Organizations:**

Organizations dedicated to uniting diverse medical students and/or residents and providing a safe space for open conversations, resources to promote their personal and professional career development, and a structure to champion advocacy issues.

**Relevance of Medical Students Being Engaged in National Medical Student Organizations (Diversity Related):**

Students provide much of the leadership in national medical student organizations, and as such, determine the vision, mission, goals and strategic plan. Trainees can interface with concordant peers and faculty, and serve as role models, mentors, and champions for underclassmen. Engagement in diversity related medical student organizations provides an opportunity to develop communication skills on relevant topics in a supportive environment, share best practices to facilitate diversity change, and develop leadership competencies.

| Leadership and Engagement Competencies^3^ Achievable Through Student Organizations Activities^1^ | | | | | |
| --- | --- | --- | --- | --- | --- |
| 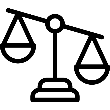  **Self-management:** demonstrates contribution to the development of effective organizational structure and allows members to display talents and responsibilities | | 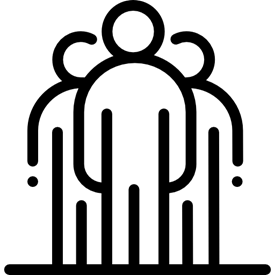  **Working with/developing others:**  mentor peers in leadership skills in serving as officers in the organization | | **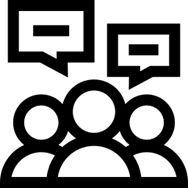**  **Communication skills**: facilitates the transfer of new ideas, potential collaborators, and the expansion of the organization | |
| **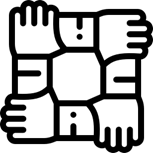**    **Teambuilding**: works with peers to problem solve, build and make decisions on behalf of the organization | **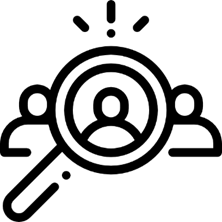**    **Leadership:** understands the structure and culture of the organization and leads the members with a vision, mission, goals and strategic planning | | 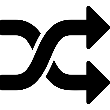    **Leading change:** understands change management through new membership, leadership and revised vision, mission, goals, and strategic plan | | 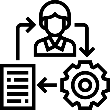  **Business skills:** evaluates and improves the organizations policies and procedures through quality improvement efforts |

|  | *Types and year of participation varies by institution. | 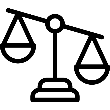 | 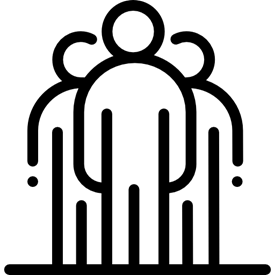 | **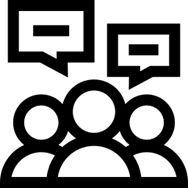** | **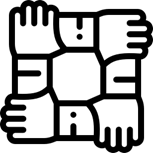** | 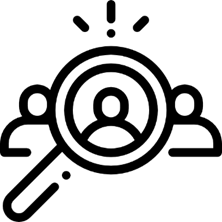 | 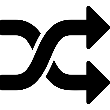 | 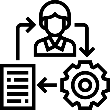 |
| --- | --- | --- | --- | --- | --- | --- | --- | --- |
| Year 1-4* | **Coordinates Efforts at Home Institution**  Engages with institutional or inter-institutional peers and leaders to design, implement and evaluate events that support the mission of their organization and institution(s). |  | **✔** | **✔** | **✔** |  |  |  |
| Year 1-4* | **Implements Pipeline/Pathway Initiatives**  Designs, implements, and evaluates activities to support the recruitment of members for the organization.^2^ | **✔** | **✔** | **✔** |  | **✔** |  |  |
| Year 2-4* | **Advocates for Special Topics**  Identifies emerging and on-going concerns of membership and uses organizational, state or federal channels to advocate for legislative change. |  | **✔** | **✔** |  | **✔** | **✔** |  |
| Year 2-4* | **Serves as a Local, Regional or National Student Leader**  Ensures members abide by the mission, vision, goals and strategic plan of the organization. Helps recruit and retain members and assesses their needs. | **✔** | **✔** | **✔** |  | **✔** | **✔** | **✔** |
| Future  **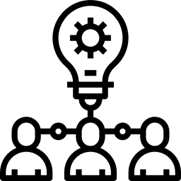** | **Leads as Organizational Advisor**  Advises members on aligning efforts with the national agenda, addressing the personal/professional development needs of members, and secures resources. Aspires for a larger role as chapter advisor, trustee, or executive director. | **✔** | **✔** | **✔** | **✔** | **✔** | **✔** | **✔** |

**Acknowledgements:** We thank FlatIcon for the access and use of their free vector icons **References:**

1. Lucas R, Goldman EF., Scott AR et al. Leadership Development Programs at Academic Health Centers: Results of a National Survey. Academic Medicine. 93(2):229-236, February 2018.

2. Rumala, Bernice B., and Frederick D. Cason Jr. "Recruitment of underrepresented minority students to medical school: minority medical student organizations, an untapped resource." *Journal of the National Medical Association* 99.9 (2007): 1000.

Icon made by Surang from [www.flaticon.com](http://www.flaticon.com) Free for personal and commercial purpose with attribution. <https://www.flaticon.com/free-icon/team_1124812?term=teamwork&page=1&position=26>

Icon made by Freepik from www.flaticon.com Free for personal and commercial purpose with attribution. <https://www.flaticon.com/free-icon/recruitment_942830?term=recruit&page=1&position=51>

Icon made by Kiranshastry from [www.flaticon.com](http://www.flaticon.com) Free for personal and commercial purpose with attribution. <https://www.flaticon.com/free-icon/balance_1153269?term=balance&page=1&position=52>

Icon made by Freepik from www.flaticon.com Free for personal and commercial purpose with attribution. <https://www.flaticon.com/free-icon/teamwork_921332>

Icon made by Freepik from www.flaticon.com Free for personal and commercial purpose with attribution.<https://www.flaticon.com/free-icon/target_1605401?term=support&page=1&position=8>

Icon made by Those Icons from www.flaticon.com Free for personal and commercial purpose with attribution. <https://www.flaticon.com/free-icon/shuffle_2089813?term=change&page=1&position=44>

Icon made by Eucalyp from www.flaticon.com Free for personal and commercial purpose with attribution. <https://www.flaticon.com/free-icon/process_1556324>

Icon made by Freepik from www.flaticon.com Free for personal and commercial purpose with attribution. <https://www.flaticon.com/free-icon/group_909337>

Icon made by Freepik from www.flaticon.com Free for personal and commercial purpose with attribution. <https://www.flaticon.com/free-icon/team_478536>
